# Exploring the relationship between frailty and nonunion fractures in upper extremity injuries: insights from the national inpatient sample

**DOI:** 10.1007/s00590-025-04247-y

**Published:** 2025-03-14

**Authors:** Cyrus Luczkow, Victor Koltenyuk, Ethan Parisier, Audrey Huang, Omri Ayalon

**Affiliations:** 1 New York Medical College, Valhalla, NY USA; 2 New York University Langone Medical Center, New York, NY USA

**Keywords:** Frailty, Nonunion fractures, Upper extremity injuries, Modified frailty index-5, National inpatient sample

## Abstract

**Introduction:**

Frailty, a physiological decline in functional capacity, may influence nonunion risk. This study aimed to investigate the association between frailty, as measured by the modified Frailty Index-5 (mFI-5), and the likelihood of nonunion fractures of the upper extremity.

**Methods:**

This retrospective cohort study utilized the national inpatient sample (NIS) from 2015 to 2019. Patients aged 18 and older with upper extremity fractures, identified by ICD-10-CM codes, were included. Patients were categorized into routine healing and nonunion groups. Frailty was assessed using the mFI-5, classifying patients as robust, prefrail, frail, or severely frail. Multivariate logistic regression, controlling for age, sex, and Injury Severity Score (ISS), was performed to determine the association between frailty and nonunion.

**Results:**

The study included 21,618 patients, with 3782 presenting with nonunion fractures. The median age was 69 years, and 60.5% were female. The most common fracture types in the routine healing group were forearm (40.1%), clavicle (18.4%), and humerus (16.9%), while in the nonunion group, humerus (30.4%) and scapula (32.1%) were most common. Multivariate logistic regression showed that frail and severely frail patients had a decreased risk of nonunion (OR 0.751 and 0.705, respectively, *p* < 0.001). Each unit increase in mFI-5 score was associated with a decreased risk of nonunion (OR 0.868, *p* < 0.001). Sub-analysis revealed a decreased risk of nonunion with increasing frailty for humerus, clavicle, scapula, and phalanx fractures, but no significant association for wrist, forearm, or metacarpal fractures.

**Conclusion:**

Contrary to expectations, increasing frailty, as measured by the mFI-5, was associated with a decreased risk of nonunion fractures in the upper extremity. This paradoxical finding may be due to closer medical supervision and improved treatment compliance in frail patients. Further prospective studies are needed to explore the complex interplay between frailty, treatment adherence, and fracture healing.

**Supplementary Information:**

The online version contains supplementary material available at 10.1007/s00590-025-04247-y.

## Introduction

Nonunion fractures pose a significant burden on patients and healthcare systems, as they may incur higher healthcare costs and increased risk of opioid dependence [1]. Understanding factors that contribute to nonunion is essential to improving patient outcomes and optimizing healthcare resource allocation [2]. It has previously been found that factors such as smoking, diabetes, open fractures, obesity, and infection increase the risk of nonunion fractures [2, 3]. As the United States population ages, the number of elderly patients presenting with fractures will likely increase [4]. Thus, it is prudent to identify patients at elevated risk of developing nonunion. One potential avenue to quantify such risk is through an assessment of frailty.

Frailty is defined as a physiological decrease in functional capacity, leading to less tolerance following insults like surgery [5–7]. Multiple frailty indices, including the modified five (mFI-5) and eleven (mFI-11) factor indices have been demonstrated to predict adverse outcomes following orthopedic surgery [8, 9]. Dent et al. found that frail women were more likely to sustain falls and long term hospitalizations [10]. However, Zura et al. found that increasing age was associated with a decreased risk of one year nonunion [11]. Considering this paradoxical relationship between increasing age and nonunion risk, frailty assessment may play a role in understanding a patient's likelihood for nonunion and provide a path toward quantifying that risk. To date, no studies have explored the association between frailty and likelihood of nonunion. To fill this gap, our study analyzed the national inpatient sample (NIS) to evaluate frailty as rated by the mFI-5 as a predictor of patients being admitted and treated for nonunion fractures of the upper extremity.

## Methods

### Data collection

The NIS is a de-identified database reflecting hospital discharges in the United States. The NIS contains a 20% representative sample and utilizes a weighting variable to extrapolate estimates to the national population. Data collection was accomplished using International Classification of Disease, 10th revision, Clinical Modification (ICD-10-CM) diagnosis and procedure codes. As the NIS is a publicly available database, institutional review board approval was not required. All ICD-10 codes used for analysis were obtained using the 2023 Healthcare Cost and Utilization Project ICD code directory and can be found in the supplemental file or upon request.

### Inclusion criteria and outcomes

The NIS database was queried for patients presenting with upper extremity fractures from 2015 to 2019. Upper extremity fractures were chosen in order to control weight bearing differences in robust versus frail patients. Patients were identified using ‘subsequent’ encounter ICD-10-CM codes for both routine healing and nonunion fractures of the humerus, forearm (radius and/or ulna), clavicle, metacarpal, phalanx, wrist, or scapula. Exclusion criteria included patients younger than 18, and those presenting with malunion fractures (Supplemental 1). The mFI-5 was chosen because it is a well-established frailty score in orthopedics and previously validated using the NIS database [8, 12–16]. The variables included in the mFI-5 were collected using ICD-10 diagnosis codes (congestive heart failure, diabetes mellitus, chronic obstructive pulmonary disease, dependent functional status, and hypertension) (Supplemental 2). For each patient, one point was allocated for each diagnosis, and a cumulative raw score was calculated. Patients were then classified as not frail (score = 0), prefrail (score = 1), frail (score = 2), or severely frail (score ≥ 3). An injury severity score was determined by computing the total number of ICD codes consistent with traumatic injuries for each patient. Additionally, baseline demographic data were collected, including age, sex, and race.

### Statistical analysis

Patients were stratified according to frailty status and baseline demographics as well as number of routine healing and nonunion fractures were determined. Categorical variables were assessed using Chi-square test and presented as counts with percentages. Continuous variables were assessed using Kruskal–Wallis test for continuous variables and presented as medians with 25th–75th interquartile ranges. Multivariate logistic regression controlling for age, sex, and injury severity was performed to determine the likelihood of nonunion. A subanalysis for each fracture type was performed using the same covariates to account for differences in fracture type. Backwards stepwise p-value removal was used to build the initial model. Only variables significant at 0.05 were retained in the final model. Statistical analysis was performed using Statistical Package for the Social Sciences (SPSS, Version 29; IBM, Inc., Armonk, NY).

## Results

### Baseline characteristics

Our study included 21,618 patients aged 18 and older presenting for follow-up for fractures of the upper extremity as determined by ‘subsequent encounter’ ICD codes. They were classified as routine healing (n = 17,836) or nonunion (n = 3782). The median age was 69 (55–81), of which 60.5% were female, 75.4% were white, 7.8% were black, and 8.3% were Hispanic. The most common comorbidity included hypertension (57.3%), followed by diabetes mellitus (24.7%), and COPD (21.8%). After determining each patient's mFI-5 score, the median score for the cohort was 1 (1–2) (Table 1).

**Table 1:** Baseline patient demographics as a percentage of the total number of routine healing and nonunion fractures

Variable	Total (*n* = 21,618)
Age	69 (55–81)
Female	13,088 (60.5%)
Race/ethnicity	
White	16,317 (75.4%)
Black	1706 (7.8%)
Hispanic	1815 (8.3%)
Comorbidities	
Hypertension	12,935 (57.3%)
Diabetes mellitus	5340 (24.7%)
Congestive heart failure	3097 (14.3%)
Chronic obstructive pulmonary disease	4719 (21.8%)
Functionally dependent	3486 (16.0%)
mFI-5 median score	1 (1–2)

### Fracture type distribution

For the routine healing group, the most common fracture type overall was forearm (40.1%), followed by clavicle (18.4%), and humerus (16.9%). For the robust group, the most common was forearm (43.9%) followed by metacarpal (16.2%) and humerus (16.8%). In the prefrail, frail, and severe frail groups, the most common fractures were forearm (41.5%, 38.4%, and 34.2%) followed by clavicle (17.6%, 21.9%, 26.5%) and humerus (17.1%, 16.8%, 17.1%). In the nonunion group, the most common overall fracture type was humerus (30.4%) followed by scapula (32.1%). In the robust, prefrail, frail, and severe frail patients, the most frequent fractures were humerus (28.8%, 30.8%, 31.9%, 29.5%), scapula (28.6%, 33.2%, 32.3%, 35.4%), and clavicle (14.6%, 22.3%, 24.6%, 25.3%), respectively (Table 2).

**Table 2:** Distribution of frailty and fracture type by frailty tiers, including routine healing and nonunion fractures

Routine healing	Overall (n = 17,836)	Robust (n = 4512)	Prefrail (n = 5696)	Frail (n = 4718)	Severe frail (n = 2910)
Humerus	3020 (16.9%)	760 (16.8%)	972 (17.1%)	791 (16.8%)	497 (17.1%)
Clavicle	3279 (18.4%)	471 (10.4%)	1003 (17.6%)	1034 (21.9%)	771 (26.5%)
Forearm	7151 (40.1%)	1979 (43.9%)	2364 (41.5%)	1812 (38.4%)	996 (34.2%)
Wrist	2486 (13.9%)	659 (14.6%)	771 (13.5%)	639 (13.5%)	417 (14.3%)
Metacarpal	1724 (9.7%)	731 (16.2%)	526 (9.2%)	321 (6.8%)	146 (5.0%)
Phalanx	1340 (7.5%)	445 (9.9%)	448 (7.9%)	295 (6.3%)	152 (5.2%)
Scapula	261 (1.5%)	46 (1.0%)	76 (1.3%)	76 (1.6%)	53 (1.8%)

Nonunion	Overall (n = 3782)	Robust (n = 1009)	Prefrail (n = 1210)	Frail (n = 1018)	Severe Frail (n = 545)
Humerus	1150 (30.4%)	291 (28.8%)	373 (30.8%)	325 (31.9%)	161 (29.5%)
Clavicle	805 (21.30%)	147 (14.60%)	270 (22.3%)	250 (24.6%)	138 (25.30%)
Forearm	446 (11.8%)	171 (16.9%)	138 (11.4%)	91 (8.9%)	46 (8.4%)
Metacarpal	42 (1.10%)	28 (2.8%)	8 (0.70%)	4 (0.40%)	2 (0.4%)
Phalanx	58 (1.5%)	32 (3.2%)	15 (1.20%)	8 (0.80%)	3 (0.60%)
Wrist	123 (3.30%)	66 (6.5%)	29 (2.40%)	21 (2.10%)	7 (1.30%)
Scapula	1213 (32.1%)	289 (28.6%)	402 (33.2%)	329 (32.30%)	193 (35.40%)

### Logistic regression

A multivariate regression controlling for age, female sex, and Injury Severity Score (ISS) was performed to examine the impact of frailty on nonunion. Frail and severe frail patients were at decreased risk of nonunion (OR: 0.751 95% 0.675–0.835, *p* < 0.001 and OR: 0.705 95% 0.609–0.816, *p* < 0.001, respectively). Analysis of the raw mFI-5 score demonstrated a decrease in risk of nonunion for each additional unit of frailty (OR: 0.868, 95% CI 0.834–0.903, *p* < 0.001). A subanalysis was conducted for each fracture to investigate the influence of fracture type. The mFI-5 was significantly associated with a decreased risk of nonunion fractures of the humerus (OR: 0.880 95% 0.809–0.958, *p* = 0.003), clavicle (OR: 0.790 95% 0.721–0.866, *p* < 0.001), scapula (OR: 0.774 95% 0.660–0.909, *p* = 0.002), and phalanx (OR: 0.626 95% 0.414–0.944, *p* = 0.026). There was no significant association between frailty and nonunion fractures of the wrist, forearm, or metacarpal (Tables 3, 4, 5).

**Table 3:** Multivariate regression for outcome of nonunion fracture controlling for ISS, age, and female sex. Frailty stratified by robust (mFI-5 = 0), prefrail (mFI-5 = 1), frail (mFI-5 = 2), and severe frail (mFI-5 > 2). Odds ratios presented with 95% confidence intervals. Significance considered p < 0.05

Variable	Odds ratio (95% C.I)	*p*-value
mFI-5		
Robust	1	1
Prefrail	1.060 (0.975–1.153)	0.173
Frail	0.751 (0.675–0.835)	< 0.001
Severe frail	0.705 (0.609–0.816)	< 0.001
ISS	1.135 (1.107–1.163)	< 0.001
Age	0.992 (0.990–0.994)	< 0.001
Female	1.301 (1.213–1.395)	< 0.001

**Table 4:** Multivariate regression for outcome of nonunion fracture controlling for ISS, age, and female sex. The continuous, raw mFI-5 variable used as a predictor. Odds ratios presented with 95% confidence intervals. Significance considered *p* < 0.05

Variable	Odds ratio (95% C.I)	*p*-value
mFI-5 raw score	0.868 (0.834–0.903)	< 0.001
ISS	1.134 (1.106–1.162)	< 0.001
Age	0.993 (0.991–0.995)	< 0.001
Female	1.300 (1.212–1.394)	< 0.001

**Table 5:** Multivariate regression for nonunion for each fracture type. Fracture types were analyzed individually. Each model controlled for age, sex, and injury severity score. Continuous mFI-5 score was the predictor used. Odds ratios presented with 95% confidence intervals. Significance considered *p* < 0.05

Nonunion type	Odds ratio (95% CI)	*p*-value
Humerus	0.880 (0.809–0.958)	**0.003**
Clavicle	0.790 (0.721–0.866)	** < 0.001**
Wrist	0.914 (0.689–1.213)	0.535
Scapula	0.774 (0.660–0.909)	**0.002**
Forearm	0.913 (0.803–1.039)	0.166
Metacarpal	0.752 (0.447–1.265)	0.282
Phalanx	0.626 (0.414–0.944)	**0.026**

Bold values are statistically significant (*P* < 0.05)

## Discussion

Fracture nonunion is a feared complication that can carry with it a significant economic and medical burden for patients including chronic pain and need for reoperation [17–20]. As the United States population ages, it is critical to identify patients at risk of nonunion. Several scores have been developed to predict nonunion risk, however frailty assessment is yet to be examined [21, 22]. Our analysis of 21,618 patients presenting with routine healing or nonunion fractures found that frailty as measured by the mFI-5 is associated with decreased risk of nonunion.

The mFI-5 has previously been demonstrated to be a robust predictor of adverse outcomes following a range of orthopedic surgeries such as total joint arthroplasty, spinal fusion, and fracture fixation [8, 9, 12, 15, 16, 23]. Frailty has also been associated with an increased risk for falls and fracture related hospitalization among elderly women [10]. Previous analyses have shown an increased risk of nonunion among patients with diabetes, opioid use disorder and alcohol abuse [1, 3]. However, our study is the first to explore the influence of frailty on likelihood of presentation with nonunion fractures. Interestingly, we found that increasing frailty was associated with a decreased risk of nonunion. These findings are in line with those from Zura et al. who demonstrated that increasing age was associated with decreased risk of delayed bone healing [11]. This paradoxical relationship may be because older and frailer patients are often subject to closer medical supervision and may adhere more rigorously to post-injury care protocols, including fracture immobilization and rehabilitation regimens [17, 24]. Moreover, it may also be because a subset of this population dies, and is lost to follow-up prior to presenting with a nonunion injury. Enhanced compliance may contribute to improved healing outcomes in this cohort. They may also have other lifestyle factors, such as vitamin supplements or medications, that influence fracture outcomes, although there is limited evidence that vitamin supplementation alone influences nonunion risk [25]. The NIS database does not capture data on patient adherence to prescribed treatments, so this hypothesis remains speculative, indicating an avenue for further research.

Another element that is critical to consider is the impact of fracture severity on nonunion. Specifically, the potential difference in injury severity between frail and non-frail patients. Prior studies have established that high-energy fractures, particularly those with open wounds or severe soft-tissue injury, are more prone to complications such as nonunion [26]. Our study found that for every unit increase in injury severity score, the likelihood of nonunion increased by 1.34 times. Furthermore, even after controlling for ISS, frailty was associated with a decreased risk of nonunion.

The mFI-5 has shown potential to be a valuable preoperative assessment tool, but its use in the prediction of nonunion is limited, as it fails to capture components predictive of delayed bone healing, such as elevated BMI, NSAID use, and vascular insufficiency [17, 19, 20]. In fact, after examining the impact of frailty on nonunion of individual fracture types, we showed that the mFI-5 was not significantly associated with wrist, forearm, and metacarpal nonunions. Our findings suggest that frailty is likely not helpful on its own when identifying patients at risk of nonunion—and that other patient characteristics are influencing this risk. Future prospective studies are needed to collect detailed information on treatment compliance and assess its potential role in fracture healing, particularly in vulnerable populations, like the frail elderly. However, it is important to note that the conclusions of our study are limited to frailty as its defined by the mFI-5. Moreover, other frailty indices such as the Risk Analysis Index should be evaluated in their predictive abilities, as each score captures different elements of a patient's medical history [27]. Specifically, our supplemental analysis showed that diabetes and CHF were associated with elevated nonunion risk, thus predictive scores that incorporate these comorbidities may be of value.

Our study has several limitations that must be addressed. The reliance on ICD-10 coding for data extraction introduces the possibility of misclassification and miscoding. Treatment protocols differ at different institutions; thus our patients were not standardized in terms of management for their initial fracture. Moreover, we lacked information on operative vs. nonoperative treatment of their initial injuries, as well as fixation type employed. Additionally, frail patients, given their multiple comorbidities and advanced age, may be more prone to mortality or loss to follow-up before a nonunion diagnosis can be established. Since the NIS dataset lacks the capacity to track patients longitudinally, the early death of frail patients or insufficient follow-up duration may have contributed to an underrepresentation of nonunion cases in this group. This limitation underscores the need for future research utilizing prospective, longitudinal study designs to ensure complete follow-up and accurate assessment of nonunion rates among frail patients. These limitations highlight the challenges of relying on administrative datasets and reinforce the need for detailed clinical documentation in future studies to ensure more accurate patient selection. Despite these limitations, the vast sample size of the database does help to account for these errors, as well as our inclusion of a broad number of ICD-10 codes to capture as near to the entirety of the patient population as possible.

In conclusion, while frailty is commonly associated with increased surgical risk and poorer outcomes, this study suggests that frailer patients may paradoxically have a lower likelihood of developing nonunion fractures. These findings challenge conventional wisdom and highlight the complexity of the interplay between frailty and fracture healing. Clinicians should continue to incorporate frailty assessments into preoperative planning and use this research as a starting point for future investigations. Future research should focus on expanding beyond what was accounted for here and begin to develop prospective studies that account for patient compliance, nutritional status, and lifestyle choices, to provide a more comprehensive understanding of nonunion fracture risk in frail populations.

## Supplementary Information

Below is the link to the electronic supplementary material.

Supplementary file1 (PDF 316 kb)

Supplementary file2 (PDF 311 kb)

## Data Availability

No datasets were generated or analyzed during the current study.
