# Supplementary material for: Exploring the relationship between frailty and nonunion fractures in upper extremity injuries: insights from the national inpatient sample
Source: Eur J Orthop Surg Traumatol. 2025 Mar 14;35(1):115. doi: 10.1007/s00590-025-04247-y (PMC11909083; doi:10.1007/s00590-025-04247-y)
Supplement: Supplementary file 1 — Supplementary file1 (PDF 316 kb) [file 590_2025_4247_MOESM1_ESM.pdf]

**Appendix 1**

Routine Healing ICD-10 Codes

| ICD-10-CM Code | ICD-10-CM Code Description |
|---|---|
| S42001D | Fracture of unspecified part of right clavicle, subsequent encounter for fracture with routine healing |
| S42002D | Fracture of unspecified part of left clavicle, subsequent encounter for fracture with routine healing |
| S42009D | Fracture of unspecified part of unspecified clavicle, subsequent encounter for fracture with routine healing |
| S42011D | Anterior displaced fracture of sternal end of right clavicle, subsequent encounter for fracture with routine healing |
| S42012D | Anterior displaced fracture of sternal end of left clavicle, subsequent encounter for fracture with routine healing |
| S42013D | Anterior displaced fracture of sternal end of unspecified clavicle, subsequent encounter for fracture with routine healing |
| S42014D | Posterior displaced fracture of sternal end of right clavicle, subsequent encounter for fracture with routine healing |
| S42015D | Posterior displaced fracture of sternal end of left clavicle, subsequent encounter for fracture with routine healing |
| S42016D | Posterior displaced fracture of sternal end of unspecified clavicle, subsequent encounter for fracture with routine healing |
| S42017D | Nondisplaced fracture of sternal end of right clavicle, subsequent encounter for fracture with routine healing |
| S42018D | Nondisplaced fracture of sternal end of left clavicle, subsequent encounter for fracture with routine healing |
| S42019D | Nondisplaced fracture of sternal end of unspecified clavicle, subsequent encounter for fracture with routine healing |
| S42021D | Displaced fracture of shaft of right clavicle, subsequent encounter for fracture with routine healing |
| S42022D | Displaced fracture of shaft of left clavicle, subsequent encounter for fracture with routine healing |

| | |
|---|---|
| S42023D | Displaced fracture of shaft of unspecified clavicle, subsequent encounter for fracture with routine healing |
| S42024D | Nondisplaced fracture of shaft of right clavicle, subsequent encounter for fracture with routine healing |
| S42025D | Nondisplaced fracture of shaft of left clavicle, subsequent encounter for fracture with routine healing |
| S42026D | Nondisplaced fracture of shaft of unspecified clavicle, subsequent encounter for fracture with routine healing |
| S42031D | Displaced fracture of lateral end of right clavicle, subsequent encounter for fracture with routine healing |
| S42032D | Displaced fracture of lateral end of left clavicle, subsequent encounter for fracture with routine healing |
| S42033D | Displaced fracture of lateral end of unspecified clavicle, subsequent encounter for fracture with routine healing |
| S42034D | Nondisplaced fracture of lateral end of right clavicle, subsequent encounter for fracture with routine healing |
| S42035D | Nondisplaced fracture of lateral end of left clavicle, subsequent encounter for fracture with routine healing |
| S42036D | Nondisplaced fracture of lateral end of unspecified clavicle, subsequent encounter for fracture with routine healing |
| S42101D | Fracture of unspecified part of scapula, right shoulder, subsequent encounter for fracture with routine healing |
| S42102D | Fracture of unspecified part of scapula, left shoulder, subsequent encounter for fracture with routine healing |
| S42109D | Fracture of unspecified part of scapula, unspecified shoulder, subsequent encounter for fracture with routine healing |
| S42111D | Displaced fracture of body of scapula, right shoulder, subsequent encounter for fracture with routine healing |
| S42112D | Displaced fracture of body of scapula, left shoulder, subsequent encounter for fracture with routine healing |
| S42113D | Displaced fracture of body of scapula, unspecified shoulder, subsequent encounter for fracture with routine healing |

| | |
|---|---|
| S42114D | Nondisplaced fracture of body of scapula, right shoulder, subsequent encounter for fracture with routine healing |
| S42115D | Nondisplaced fracture of body of scapula, left shoulder, subsequent encounter for fracture with routine healing |
| S42116D | Nondisplaced fracture of body of scapula, unspecified shoulder, subsequent encounter for fracture with routine healing |
| S42121D | Displaced fracture of acromial process, right shoulder, subsequent encounter for fracture with routine healing |
| S42122D | Displaced fracture of acromial process, left shoulder, subsequent encounter for fracture with routine healing |
| S42123D | Displaced fracture of acromial process, unspecified shoulder, subsequent encounter for fracture with routine healing |
| S42124D | Nondisplaced fracture of acromial process, right shoulder, subsequent encounter for fracture with routine healing |
| S42125D | Nondisplaced fracture of acromial process, left shoulder, subsequent encounter for fracture with routine healing |
| S42126D | Nondisplaced fracture of acromial process, unspecified shoulder, subsequent encounter for fracture with routine healing |
| S42131D | Displaced fracture of coracoid process, right shoulder, subsequent encounter for fracture with routine healing |
| S42132D | Displaced fracture of coracoid process, left shoulder, subsequent encounter for fracture with routine healing |
| S42133D | Displaced fracture of coracoid process, unspecified shoulder, subsequent encounter for fracture with routine healing |
| S42134D | Nondisplaced fracture of coracoid process, right shoulder, subsequent encounter for fracture with routine healing |
| S42135D | Nondisplaced fracture of coracoid process, left shoulder, subsequent encounter for fracture with routine healing |
| S42136D | Nondisplaced fracture of coracoid process, unspecified shoulder, subsequent encounter for fracture with routine healing |
| S42141D | Displaced fracture of glenoid cavity of scapula, right shoulder, subsequent encounter for fracture with routine healing |

| | |
|---|---|
| S42142D | Displaced fracture of glenoid cavity of scapula, left shoulder, subsequent encounter for fracture with routine healing |
| S42143D | Displaced fracture of glenoid cavity of scapula, unspecified shoulder, subsequent encounter for fracture with routine healing |
| S42144D | Nondisplaced fracture of glenoid cavity of scapula, right shoulder, subsequent encounter for fracture with routine healing |
| S42145D | Nondisplaced fracture of glenoid cavity of scapula, left shoulder, subsequent encounter for fracture with routine healing |
| S42146D | Nondisplaced fracture of glenoid cavity of scapula, unspecified shoulder, subsequent encounter for fracture with routine healing |
| S42151D | Displaced fracture of neck of scapula, right shoulder, subsequent encounter for fracture with routine healing |
| S42152D | Displaced fracture of neck of scapula, left shoulder, subsequent encounter for fracture with routine healing |
| S42153D | Displaced fracture of neck of scapula, unspecified shoulder, subsequent encounter for fracture with routine healing |
| S42154D | Nondisplaced fracture of neck of scapula, right shoulder, subsequent encounter for fracture with routine healing |
| S42155D | Nondisplaced fracture of neck of scapula, left shoulder, subsequent encounter for fracture with routine healing |
| S42156D | Nondisplaced fracture of neck of scapula, unspecified shoulder, subsequent encounter for fracture with routine healing |
| S42191D | Fracture of other part of scapula, right shoulder, subsequent encounter for fracture with routine healing |
| S42192D | Fracture of other part of scapula, left shoulder, subsequent encounter for fracture with routine healing |
| S42199D | Fracture of other part of scapula, unspecified shoulder, subsequent encounter for fracture with routine healing |
| S42201D | Unspecified fracture of upper end of right humerus, subsequent encounter for fracture with routine healing |
| S42202D | Unspecified fracture of upper end of left humerus, subsequent encounter for fracture with routine healing |

| | |
|---|---|
| S42209D | Unspecified fracture of upper end of unspecified humerus, subsequent encounter for fracture with routine healing |
| S42211D | Unspecified displaced fracture of surgical neck of right humerus, subsequent encounter for fracture with routine healing |
| S42212D | Unspecified displaced fracture of surgical neck of left humerus, subsequent encounter for fracture with routine healing |
| S42213D | Unspecified displaced fracture of surgical neck of unspecified humerus, subsequent encounter for fracture with routine healing |
| S42214D | Unspecified nondisplaced fracture of surgical neck of right humerus, subsequent encounter for fracture with routine healing |
| S42215D | Unspecified nondisplaced fracture of surgical neck of left humerus, subsequent encounter for fracture with routine healing |
| S42216D | Unspecified nondisplaced fracture of surgical neck of unspecified humerus, subsequent encounter for fracture with routine healing |
| S42221D | 2-part displaced fracture of surgical neck of right humerus, subsequent encounter for fracture with routine healing |
| S42222D | 2-part displaced fracture of surgical neck of left humerus, subsequent encounter for fracture with routine healing |
| S42223D | 2-part displaced fracture of surgical neck of unspecified humerus, subsequent encounter for fracture with routine healing |
| S42224D | 2-part nondisplaced fracture of surgical neck of right humerus, subsequent encounter for fracture with routine healing |
| S42225D | 2-part nondisplaced fracture of surgical neck of left humerus, subsequent encounter for fracture with routine healing |
| S42226D | 2-part nondisplaced fracture of surgical neck of unspecified humerus, subsequent encounter for fracture with routine healing |
| S42231D | 3-part fracture of surgical neck of right humerus, subsequent encounter for fracture with routine healing |
| S42232D | 3-part fracture of surgical neck of left humerus, subsequent encounter for fracture with routine healing |
| S42239D | 3-part fracture of surgical neck of unspecified humerus, subsequent encounter for fracture with routine healing |

| | |
|---|---|
| S42241D | 4-part fracture of surgical neck of right humerus, subsequent encounter for fracture with routine healing |
| S42242D | 4-part fracture of surgical neck of left humerus, subsequent encounter for fracture with routine healing |
| S42249D | 4-part fracture of surgical neck of unspecified humerus, subsequent encounter for fracture with routine healing |
| S42251D | Displaced fracture of greater tuberosity of right humerus, subsequent encounter for fracture with routine healing |
| S42252D | Displaced fracture of greater tuberosity of left humerus, subsequent encounter for fracture with routine healing |
| S42253D | Displaced fracture of greater tuberosity of unspecified humerus, subsequent encounter for fracture with routine healing |
| S42254D | Nondisplaced fracture of greater tuberosity of right humerus, subsequent encounter for fracture with routine healing |
| S42255D | Nondisplaced fracture of greater tuberosity of left humerus, subsequent encounter for fracture with routine healing |
| S42256D | Nondisplaced fracture of greater tuberosity of unspecified humerus, subsequent encounter for fracture with routine healing |
| S42261D | Displaced fracture of lesser tuberosity of right humerus, subsequent encounter for fracture with routine healing |
| S42262D | Displaced fracture of lesser tuberosity of left humerus, subsequent encounter for fracture with routine healing |
| S42263D | Displaced fracture of lesser tuberosity of unspecified humerus, subsequent encounter for fracture with routine healing |
| S42264D | Nondisplaced fracture of lesser tuberosity of right humerus, subsequent encounter for fracture with routine healing |
| S42265D | Nondisplaced fracture of lesser tuberosity of left humerus, subsequent encounter for fracture with routine healing |
| S42266D | Nondisplaced fracture of lesser tuberosity of unspecified humerus, subsequent encounter for fracture with routine healing |
| S42271D | Torus fracture of upper end of right humerus, subsequent encounter for fracture with routine healing |

| | |
|---|---|
| S42272D | Torus fracture of upper end of left humerus, subsequent encounter for fracture with routine healing |
| S42279D | Torus fracture of upper end of unspecified humerus, subsequent encounter for fracture with routine healing |
| S42291D | Other displaced fracture of upper end of right humerus, subsequent encounter for fracture with routine healing |
| S42292D | Other displaced fracture of upper end of left humerus, subsequent encounter for fracture with routine healing |
| S42293D | Other displaced fracture of upper end of unspecified humerus, subsequent encounter for fracture with routine healing |
| S42294D | Other nondisplaced fracture of upper end of right humerus, subsequent encounter for fracture with routine healing |
| S42295D | Other nondisplaced fracture of upper end of left humerus, subsequent encounter for fracture with routine healing |
| S42296D | Other nondisplaced fracture of upper end of unspecified humerus, subsequent encounter for fracture with routine healing |
| S42301D | Unspecified fracture of shaft of humerus, right arm, subsequent encounter for fracture with routine healing |
| S42302D | Unspecified fracture of shaft of humerus, left arm, subsequent encounter for fracture with routine healing |
| S42309D | Unspecified fracture of shaft of humerus, unspecified arm, subsequent encounter for fracture with routine healing |
| S42311D | Greenstick fracture of shaft of humerus, right arm, subsequent encounter for fracture with routine healing |
| S42312D | Greenstick fracture of shaft of humerus, left arm, subsequent encounter for fracture with routine healing |
| S42319D | Greenstick fracture of shaft of humerus, unspecified arm, subsequent encounter for fracture with routine healing |
| S42321D | Displaced transverse fracture of shaft of humerus, right arm, subsequent encounter for fracture with routine healing |
| S42322D | Displaced transverse fracture of shaft of humerus, left arm, subsequent encounter for fracture with routine healing |

| | |
|---|---|
| S42323D | Displaced transverse fracture of shaft of humerus, unspecified arm, subsequent encounter for fracture with routine healing |
| S42324D | Nondisplaced transverse fracture of shaft of humerus, right arm, subsequent encounter for fracture with routine healing |
| S42325D | Nondisplaced transverse fracture of shaft of humerus, left arm, subsequent encounter for fracture with routine healing |
| S42326D | Nondisplaced transverse fracture of shaft of humerus, unspecified arm, subsequent encounter for fracture with routine healing |
| S42331D | Displaced oblique fracture of shaft of humerus, right arm, subsequent encounter for fracture with routine healing |
| S42332D | Displaced oblique fracture of shaft of humerus, left arm, subsequent encounter for fracture with routine healing |
| S42333D | Displaced oblique fracture of shaft of humerus, unspecified arm, subsequent encounter for fracture with routine healing |
| S42334D | Nondisplaced oblique fracture of shaft of humerus, right arm, subsequent encounter for fracture with routine healing |
| S42335D | Nondisplaced oblique fracture of shaft of humerus, left arm, subsequent encounter for fracture with routine healing |
| S42336D | Nondisplaced oblique fracture of shaft of humerus, unspecified arm, subsequent encounter for fracture with routine healing |
| S42341D | Displaced spiral fracture of shaft of humerus, right arm, subsequent encounter for fracture with routine healing |
| S42342D | Displaced spiral fracture of shaft of humerus, left arm, subsequent encounter for fracture with routine healing |
| S42343D | Displaced spiral fracture of shaft of humerus, unspecified arm, subsequent encounter for fracture with routine healing |
| S42344D | Nondisplaced spiral fracture of shaft of humerus, right arm, subsequent encounter for fracture with routine healing |
| S42345D | Nondisplaced spiral fracture of shaft of humerus, left arm, subsequent encounter for fracture with routine healing |
| S42346D | Nondisplaced spiral fracture of shaft of humerus, unspecified arm, subsequent encounter for fracture with routine healing |

| | |
|---|---|
| S42351D | Displaced comminuted fracture of shaft of humerus, right arm, subsequent encounter for fracture with routine healing |
| S42352D | Displaced comminuted fracture of shaft of humerus, left arm, subsequent encounter for fracture with routine healing |
| S42353D | Displaced comminuted fracture of shaft of humerus, unspecified arm, subsequent encounter for fracture with routine healing |
| S42354D | Nondisplaced comminuted fracture of shaft of humerus, right arm, subsequent encounter for fracture with routine healing |
| S42355D | Nondisplaced comminuted fracture of shaft of humerus, left arm, subsequent encounter for fracture with routine healing |
| S42356D | Nondisplaced comminuted fracture of shaft of humerus, unspecified arm, subsequent encounter for fracture with routine healing |
| S42361D | Displaced segmental fracture of shaft of humerus, right arm, subsequent encounter for fracture with routine healing |
| S42362D | Displaced segmental fracture of shaft of humerus, left arm, subsequent encounter for fracture with routine healing |
| S42363D | Displaced segmental fracture of shaft of humerus, unspecified arm, subsequent encounter for fracture with routine healing |
| S42364D | Nondisplaced segmental fracture of shaft of humerus, right arm, subsequent encounter for fracture with routine healing |
| S42365D | Nondisplaced segmental fracture of shaft of humerus, left arm, subsequent encounter for fracture with routine healing |
| S42366D | Nondisplaced segmental fracture of shaft of humerus, unspecified arm, subsequent encounter for fracture with routine healing |
| S42391D | Other fracture of shaft of right humerus, subsequent encounter for fracture with routine healing |
| S42392D | Other fracture of shaft of left humerus, subsequent encounter for fracture with routine healing |
| S42399D | Other fracture of shaft of unspecified humerus, subsequent encounter for fracture with routine healing |
| S42401D | Unspecified fracture of lower end of right humerus, subsequent encounter for fracture with routine healing |

| | |
|---|---|
| S42402D | Unspecified fracture of lower end of left humerus, subsequent encounter for fracture with routine healing |
| S42409D | Unspecified fracture of lower end of unspecified humerus, subsequent encounter for fracture with routine healing |
| S42411D | Displaced simple supracondylar fracture without intercondylar fracture of right humerus, subsequent encounter for fracture with routine healing |
| S42412D | Displaced simple supracondylar fracture without intercondylar fracture of left humerus, subsequent encounter for fracture with routine healing |
| S42413D | Displaced simple supracondylar fracture without intercondylar fracture of unspecified humerus, subsequent encounter for fracture with routine healing |
| S42414D | Nondisplaced simple supracondylar fracture without intercondylar fracture of right humerus, subsequent encounter for fracture with routine healing |
| S42415D | Nondisplaced simple supracondylar fracture without intercondylar fracture of left humerus, subsequent encounter for fracture with routine healing |
| S42416D | Nondisplaced simple supracondylar fracture without intercondylar fracture of unspecified humerus, subsequent encounter for fracture with routine healing |
| S42421D | Displaced comminuted supracondylar fracture without intercondylar fracture of right humerus, subsequent encounter for fracture with routine healing |
| S42422D | Displaced comminuted supracondylar fracture without intercondylar fracture of left humerus, subsequent encounter for fracture with routine healing |
| S42423D | Displaced comminuted supracondylar fracture without intercondylar fracture of unspecified humerus, subsequent encounter for fracture with routine healing |
| S42424D | Nondisplaced comminuted supracondylar fracture without intercondylar fracture of right humerus, subsequent encounter for fracture with routine healing |
| S42425D | Nondisplaced comminuted supracondylar fracture without intercondylar fracture of left humerus, subsequent encounter for fracture with routine healing |
| S42426D | Nondisplaced comminuted supracondylar fracture without intercondylar fracture of unspecified humerus, subsequent encounter for fracture with routine healing |
| S42431D | Displaced fracture (avulsion) of lateral epicondyle of right humerus, subsequent encounter for fracture with routine healing |
| S42432D | Displaced fracture (avulsion) of lateral epicondyle of left humerus, subsequent encounter for fracture with routine healing |

| | |
|---|---|
| S42433D | Displaced fracture (avulsion) of lateral epicondyle of unspecified humerus, subsequent encounter for fracture with routine healing |
| S42434D | Nondisplaced fracture (avulsion) of lateral epicondyle of right humerus, subsequent encounter for fracture with routine healing |
| S42435D | Nondisplaced fracture (avulsion) of lateral epicondyle of left humerus, subsequent encounter for fracture with routine healing |
| S42436D | Nondisplaced fracture (avulsion) of lateral epicondyle of unspecified humerus, subsequent encounter for fracture with routine healing |
| S42441D | Displaced fracture (avulsion) of medial epicondyle of right humerus, subsequent encounter for fracture with routine healing |
| S42442D | Displaced fracture (avulsion) of medial epicondyle of left humerus, subsequent encounter for fracture with routine healing |
| S42443D | Displaced fracture (avulsion) of medial epicondyle of unspecified humerus, subsequent encounter for fracture with routine healing |
| S42444D | Nondisplaced fracture (avulsion) of medial epicondyle of right humerus, subsequent encounter for fracture with routine healing |
| S42445D | Nondisplaced fracture (avulsion) of medial epicondyle of left humerus, subsequent encounter for fracture with routine healing |
| S42446D | Nondisplaced fracture (avulsion) of medial epicondyle of unspecified humerus, subsequent encounter for fracture with routine healing |
| S42447D | Incarcerated fracture (avulsion) of medial epicondyle of right humerus, subsequent encounter for fracture with routine healing |
| S42448D | Incarcerated fracture (avulsion) of medial epicondyle of left humerus, subsequent encounter for fracture with routine healing |
| S42449D | Incarcerated fracture (avulsion) of medial epicondyle of unspecified humerus, subsequent encounter for fracture with routine healing |
| S42451D | Displaced fracture of lateral condyle of right humerus, subsequent encounter for fracture with routine healing |
| S42452D | Displaced fracture of lateral condyle of left humerus, subsequent encounter for fracture with routine healing |
| S42453D | Displaced fracture of lateral condyle of unspecified humerus, subsequent encounter for fracture with routine healing |

| | |
|---|---|
| S42454D | Nondisplaced fracture of lateral condyle of right humerus, subsequent encounter for fracture with routine healing |
| S42455D | Nondisplaced fracture of lateral condyle of left humerus, subsequent encounter for fracture with routine healing |
| S42456D | Nondisplaced fracture of lateral condyle of unspecified humerus, subsequent encounter for fracture with routine healing |
| S42461D | Displaced fracture of medial condyle of right humerus, subsequent encounter for fracture with routine healing |
| S42462D | Displaced fracture of medial condyle of left humerus, subsequent encounter for fracture with routine healing |
| S42463D | Displaced fracture of medial condyle of unspecified humerus, subsequent encounter for fracture with routine healing |
| S42464D | Nondisplaced fracture of medial condyle of right humerus, subsequent encounter for fracture with routine healing |
| S42465D | Nondisplaced fracture of medial condyle of left humerus, subsequent encounter for fracture with routine healing |
| S42466D | Nondisplaced fracture of medial condyle of unspecified humerus, subsequent encounter for fracture with routine healing |
| S42471D | Displaced transcondylar fracture of right humerus, subsequent encounter for fracture with routine healing |
| S42472D | Displaced transcondylar fracture of left humerus, subsequent encounter for fracture with routine healing |
| S42473D | Displaced transcondylar fracture of unspecified humerus, subsequent encounter for fracture with routine healing |
| S42474D | Nondisplaced transcondylar fracture of right humerus, subsequent encounter for fracture with routine healing |
| S42475D | Nondisplaced transcondylar fracture of left humerus, subsequent encounter for fracture with routine healing |
| S42476D | Nondisplaced transcondylar fracture of unspecified humerus, subsequent encounter for fracture with routine healing |
| S42481D | Torus fracture of lower end of right humerus, subsequent encounter for fracture with routine healing |

| | |
|---|---|
| S42482D | Torus fracture of lower end of left humerus, subsequent encounter for fracture with routine healing |
| S42489D | Torus fracture of lower end of unspecified humerus, subsequent encounter for fracture with routine healing |
| S42491D | Other displaced fracture of lower end of right humerus, subsequent encounter for fracture with routine healing |
| S42492D | Other displaced fracture of lower end of left humerus, subsequent encounter for fracture with routine healing |
| S42493D | Other displaced fracture of lower end of unspecified humerus, subsequent encounter for fracture with routine healing |
| S42494D | Other nondisplaced fracture of lower end of right humerus, subsequent encounter for fracture with routine healing |
| S42495D | Other nondisplaced fracture of lower end of left humerus, subsequent encounter for fracture with routine healing |
| S42496D | Other nondisplaced fracture of lower end of unspecified humerus, subsequent encounter for fracture with routine healing |
| S4290XD | Fracture of unspecified shoulder girdle, part unspecified, subsequent encounter for fracture with routine healing |
| S4291XD | Fracture of right shoulder girdle, part unspecified, subsequent encounter for fracture with routine healing |
| S4292XD | Fracture of left shoulder girdle, part unspecified, subsequent encounter for fracture with routine healing |
| S49001D | Unspecified physeal fracture of upper end of humerus, right arm, subsequent encounter for fracture with routine healing |
| S49002D | Unspecified physeal fracture of upper end of humerus, left arm, subsequent encounter for fracture with routine healing |
| S49009D | Unspecified physeal fracture of upper end of humerus, unspecified arm, subsequent encounter for fracture with routine healing |
| S49011D | Salter-Harris Type I physeal fracture of upper end of humerus, right arm, subsequent encounter for fracture with routine healing |
| S49012D | Salter-Harris Type I physeal fracture of upper end of humerus, left arm, subsequent encounter for fracture with routine healing |

| | |
|---|---|
| S49019D | Salter-Harris Type I physeal fracture of upper end of humerus, unspecified arm, subsequent encounter for fracture with routine healing |
| S49021D | Salter-Harris Type II physeal fracture of upper end of humerus, right arm, subsequent encounter for fracture with routine healing |
| S49022D | Salter-Harris Type II physeal fracture of upper end of humerus, left arm, subsequent encounter for fracture with routine healing |
| S49029D | Salter-Harris Type II physeal fracture of upper end of humerus, unspecified arm, subsequent encounter for fracture with routine healing |
| S49031D | Salter-Harris Type III physeal fracture of upper end of humerus, right arm, subsequent encounter for fracture with routine healing |
| S49032D | Salter-Harris Type III physeal fracture of upper end of humerus, left arm, subsequent encounter for fracture with routine healing |
| S49039D | Salter-Harris Type III physeal fracture of upper end of humerus, unspecified arm, subsequent encounter for fracture with routine healing |
| S49041D | Salter-Harris Type IV physeal fracture of upper end of humerus, right arm, subsequent encounter for fracture with routine healing |
| S49042D | Salter-Harris Type IV physeal fracture of upper end of humerus, left arm, subsequent encounter for fracture with routine healing |
| S49049D | Salter-Harris Type IV physeal fracture of upper end of humerus, unspecified arm, subsequent encounter for fracture with routine healing |
| S49091D | Other physeal fracture of upper end of humerus, right arm, subsequent encounter for fracture with routine healing |
| S49092D | Other physeal fracture of upper end of humerus, left arm, subsequent encounter for fracture with routine healing |
| S49099D | Other physeal fracture of upper end of humerus, unspecified arm, subsequent encounter for fracture with routine healing |
| S49101D | Unspecified physeal fracture of lower end of humerus, right arm, subsequent encounter for fracture with routine healing |
| S49102D | Unspecified physeal fracture of lower end of humerus, left arm, subsequent encounter for fracture with routine healing |
| S49109D | Unspecified physeal fracture of lower end of humerus, unspecified arm, subsequent encounter for fracture with routine healing |

| | |
|---|---|
| S49111D | Salter-Harris Type I physeal fracture of lower end of humerus, right arm, subsequent encounter for fracture with routine healing |
| S49112D | Salter-Harris Type I physeal fracture of lower end of humerus, left arm, subsequent encounter for fracture with routine healing |
| S49119D | Salter-Harris Type I physeal fracture of lower end of humerus, unspecified arm, subsequent encounter for fracture with routine healing |
| S49121D | Salter-Harris Type II physeal fracture of lower end of humerus, right arm, subsequent encounter for fracture with routine healing |
| S49122D | Salter-Harris Type II physeal fracture of lower end of humerus, left arm, subsequent encounter for fracture with routine healing |
| S49129D | Salter-Harris Type II physeal fracture of lower end of humerus, unspecified arm, subsequent encounter for fracture with routine healing |
| S49131D | Salter-Harris Type III physeal fracture of lower end of humerus, right arm, subsequent encounter for fracture with routine healing |
| S49132D | Salter-Harris Type III physeal fracture of lower end of humerus, left arm, subsequent encounter for fracture with routine healing |
| S49139D | Salter-Harris Type III physeal fracture of lower end of humerus, unspecified arm, subsequent encounter for fracture with routine healing |
| S49141D | Salter-Harris Type IV physeal fracture of lower end of humerus, right arm, subsequent encounter for fracture with routine healing |
| S49142D | Salter-Harris Type IV physeal fracture of lower end of humerus, left arm, subsequent encounter for fracture with routine healing |
| S49149D | Salter-Harris Type IV physeal fracture of lower end of humerus, unspecified arm, subsequent encounter for fracture with routine healing |
| S49191D | Other physeal fracture of lower end of humerus, right arm, subsequent encounter for fracture with routine healing |
| S49192D | Other physeal fracture of lower end of humerus, left arm, subsequent encounter for fracture with routine healing |
| S49199D | Other physeal fracture of lower end of humerus, unspecified arm, subsequent encounter for fracture with routine healing |
| S52001D | Unspecified fracture of upper end of right ulna, subsequent encounter for closed fracture with routine healing |

| | |
|---|---|
| S52001E | Unspecified fracture of upper end of right ulna, subsequent encounter for open fracture type I or II with routine healing |
| S52001F | Unspecified fracture of upper end of right ulna, subsequent encounter for open fracture type IIIA, IIIB, or IIIC with routine healing |
| S52002D | Unspecified fracture of upper end of left ulna, subsequent encounter for closed fracture with routine healing |
| S52002E | Unspecified fracture of upper end of left ulna, subsequent encounter for open fracture type I or II with routine healing |
| S52002F | Unspecified fracture of upper end of left ulna, subsequent encounter for open fracture type IIIA, IIIB, or IIIC with routine healing |
| S52009D | Unspecified fracture of upper end of unspecified ulna, subsequent encounter for closed fracture with routine healing |
| S52009E | Unspecified fracture of upper end of unspecified ulna, subsequent encounter for open fracture type I or II with routine healing |
| S52009F | Unspecified fracture of upper end of unspecified ulna, subsequent encounter for open fracture type IIIA, IIIB, or IIIC with routine healing |
| S52011D | Torus fracture of upper end of right ulna, subsequent encounter for fracture with routine healing |
| S52012D | Torus fracture of upper end of left ulna, subsequent encounter for fracture with routine healing |
| S52019D | Torus fracture of upper end of unspecified ulna, subsequent encounter for fracture with routine healing |
| S52021D | Displaced fracture of olecranon process without intraarticular extension of right ulna, subsequent encounter for closed fracture with routine healing |
| S52021E | Displaced fracture of olecranon process without intraarticular extension of right ulna, subsequent encounter for open fracture type I or II with routine healing |
| S52021F | Displaced fracture of olecranon process without intraarticular extension of right ulna, subsequent encounter for open fracture type IIIA, IIIB, or IIIC with routine healing |
| S52022D | Displaced fracture of olecranon process without intraarticular extension of left ulna, subsequent encounter for closed fracture with routine healing |
| S52022E | Displaced fracture of olecranon process without intraarticular extension of left ulna, subsequent encounter for open fracture type I or II with routine healing |

| | |
|---|---|
| S52022F | Displaced fracture of olecranon process without intraarticular extension of left ulna, subsequent encounter for open fracture type IIIA, IIIB, or IIIC with routine healing |
| S52023D | Displaced fracture of olecranon process without intraarticular extension of unspecified ulna, subsequent encounter for closed fracture with routine healing |
| S52023E | Displaced fracture of olecranon process without intraarticular extension of unspecified ulna, subsequent encounter for open fracture type I or II with routine healing |
| S52023F | Displaced fracture of olecranon process without intraarticular extension of unspecified ulna, subsequent encounter for open fracture type IIIA, IIIB, or IIIC with routine healing |
| S52024D | Nondisplaced fracture of olecranon process without intraarticular extension of right ulna, subsequent encounter for closed fracture with routine healing |
| S52024E | Nondisplaced fracture of olecranon process without intraarticular extension of right ulna, subsequent encounter for open fracture type I or II with routine healing |
| S52024F | Nondisplaced fracture of olecranon process without intraarticular extension of right ulna, subsequent encounter for open fracture type IIIA, IIIB, or IIIC with routine healing |
| S52025D | Nondisplaced fracture of olecranon process without intraarticular extension of left ulna, subsequent encounter for closed fracture with routine healing |
| S52025E | Nondisplaced fracture of olecranon process without intraarticular extension of left ulna, subsequent encounter for open fracture type I or II with routine healing |
| S52025F | Nondisplaced fracture of olecranon process without intraarticular extension of left ulna, subsequent encounter for open fracture type IIIA, IIIB, or IIIC with routine healing |
| S52026D | Nondisplaced fracture of olecranon process without intraarticular extension of unspecified ulna, subsequent encounter for closed fracture with routine healing |
| S52026E | Nondisplaced fracture of olecranon process without intraarticular extension of unspecified ulna, subsequent encounter for open fracture type I or II with routine healing |
| S52026F | Nondisplaced fracture of olecranon process without intraarticular extension of unspecified ulna, subsequent encounter for open fracture type IIIA, IIIB, or IIIC with routine healing |

| | |
|---|---|
| S52031D | Displaced fracture of olecranon process with intraarticular extension of right ulna, subsequent encounter for closed fracture with routine healing |
| S52031E | Displaced fracture of olecranon process with intraarticular extension of right ulna, subsequent encounter for open fracture type I or II with routine healing |
| S52031F | Displaced fracture of olecranon process with intraarticular extension of right ulna, subsequent encounter for open fracture type IIIA, IIIB, or IIIC with routine healing |
| S52032D | Displaced fracture of olecranon process with intraarticular extension of left ulna, subsequent encounter for closed fracture with routine healing |
| S52032E | Displaced fracture of olecranon process with intraarticular extension of left ulna, subsequent encounter for open fracture type I or II with routine healing |
| S52032F | Displaced fracture of olecranon process with intraarticular extension of left ulna, subsequent encounter for open fracture type IIIA, IIIB, or IIIC with routine healing |
| S52033D | Displaced fracture of olecranon process with intraarticular extension of unspecified ulna, subsequent encounter for closed fracture with routine healing |
| S52033E | Displaced fracture of olecranon process with intraarticular extension of unspecified ulna, subsequent encounter for open fracture type I or II with routine healing |
| S52033F | Displaced fracture of olecranon process with intraarticular extension of unspecified ulna, subsequent encounter for open fracture type IIIA, IIIB, or IIIC with routine healing |
| S52034D | Nondisplaced fracture of olecranon process with intraarticular extension of right ulna, subsequent encounter for closed fracture with routine healing |
| S52034E | Nondisplaced fracture of olecranon process with intraarticular extension of right ulna, subsequent encounter for open fracture type I or II with routine healing |
| S52034F | Nondisplaced fracture of olecranon process with intraarticular extension of right ulna, subsequent encounter for open fracture type IIIA, IIIB, or IIIC with routine healing |
| S52035D | Nondisplaced fracture of olecranon process with intraarticular extension of left ulna, subsequent encounter for closed fracture with routine healing |
| S52035E | Nondisplaced fracture of olecranon process with intraarticular extension of left ulna, subsequent encounter for open fracture type I or II with routine healing |

| | |
|---|---|
| S52035F | Nondisplaced fracture of olecranon process with intraarticular extension of left ulna, subsequent encounter for open fracture type IIIA, IIIB, or IIIC with routine healing |
| S52036D | Nondisplaced fracture of olecranon process with intraarticular extension of unspecified ulna, subsequent encounter for closed fracture with routine healing |
| S52036E | Nondisplaced fracture of olecranon process with intraarticular extension of unspecified ulna, subsequent encounter for open fracture type I or II with routine healing |
| S52036F | Nondisplaced fracture of olecranon process with intraarticular extension of unspecified ulna, subsequent encounter for open fracture type IIIA, IIIB, or IIIC with routine healing |
| S52041D | Displaced fracture of coronoid process of right ulna, subsequent encounter for closed fracture with routine healing |
| S52041E | Displaced fracture of coronoid process of right ulna, subsequent encounter for open fracture type I or II with routine healing |
| S52041F | Displaced fracture of coronoid process of right ulna, subsequent encounter for open fracture type IIIA, IIIB, or IIIC with routine healing |
| S52042D | Displaced fracture of coronoid process of left ulna, subsequent encounter for closed fracture with routine healing |
| S52042E | Displaced fracture of coronoid process of left ulna, subsequent encounter for open fracture type I or II with routine healing |
| S52042F | Displaced fracture of coronoid process of left ulna, subsequent encounter for open fracture type IIIA, IIIB, or IIIC with routine healing |
| S52043D | Displaced fracture of coronoid process of unspecified ulna, subsequent encounter for closed fracture with routine healing |
| S52043E | Displaced fracture of coronoid process of unspecified ulna, subsequent encounter for open fracture type I or II with routine healing |
| S52043F | Displaced fracture of coronoid process of unspecified ulna, subsequent encounter for open fracture type IIIA, IIIB, or IIIC with routine healing |
| S52044D | Nondisplaced fracture of coronoid process of right ulna, subsequent encounter for closed fracture with routine healing |
| S52044E | Nondisplaced fracture of coronoid process of right ulna, subsequent encounter for open fracture type I or II with routine healing |

| | |
|---|---|
| S52044F | Nondisplaced fracture of coronoid process of right ulna, subsequent encounter for open fracture type IIIA, IIIB, or IIIC with routine healing |
| S52045D | Nondisplaced fracture of coronoid process of left ulna, subsequent encounter for closed fracture with routine healing |
| S52045E | Nondisplaced fracture of coronoid process of left ulna, subsequent encounter for open fracture type I or II with routine healing |
| S52045F | Nondisplaced fracture of coronoid process of left ulna, subsequent encounter for open fracture type IIIA, IIIB, or IIIC with routine healing |
| S52046D | Nondisplaced fracture of coronoid process of unspecified ulna, subsequent encounter for closed fracture with routine healing |
| S52046E | Nondisplaced fracture of coronoid process of unspecified ulna, subsequent encounter for open fracture type I or II with routine healing |
| S52046F | Nondisplaced fracture of coronoid process of unspecified ulna, subsequent encounter for open fracture type IIIA, IIIB, or IIIC with routine healing |
| S52091D | Other fracture of upper end of right ulna, subsequent encounter for closed fracture with routine healing |
| S52091E | Other fracture of upper end of right ulna, subsequent encounter for open fracture type I or II with routine healing |
| S52091F | Other fracture of upper end of right ulna, subsequent encounter for open fracture type IIIA, IIIB, or IIIC with routine healing |
| S52092D | Other fracture of upper end of left ulna, subsequent encounter for closed fracture with routine healing |
| S52092E | Other fracture of upper end of left ulna, subsequent encounter for open fracture type I or II with routine healing |
| S52092F | Other fracture of upper end of left ulna, subsequent encounter for open fracture type IIIA, IIIB, or IIIC with routine healing |
| S52099D | Other fracture of upper end of unspecified ulna, subsequent encounter for closed fracture with routine healing |
| S52099E | Other fracture of upper end of unspecified ulna, subsequent encounter for open fracture type I or II with routine healing |
| S52099F | Other fracture of upper end of unspecified ulna, subsequent encounter for open fracture type IIIA, IIIB, or IIIC with routine healing |

| | |
|---|---|
| S52101D | Unspecified fracture of upper end of right radius, subsequent encounter for closed fracture with routine healing |
| S52101E | Unspecified fracture of upper end of right radius, subsequent encounter for open fracture type I or II with routine healing |
| S52101F | Unspecified fracture of upper end of right radius, subsequent encounter for open fracture type IIIA, IIIB, or IIIC with routine healing |
| S52102D | Unspecified fracture of upper end of left radius, subsequent encounter for closed fracture with routine healing |
| S52102E | Unspecified fracture of upper end of left radius, subsequent encounter for open fracture type I or II with routine healing |
| S52102F | Unspecified fracture of upper end of left radius, subsequent encounter for open fracture type IIIA, IIIB, or IIIC with routine healing |
| S52109D | Unspecified fracture of upper end of unspecified radius, subsequent encounter for closed fracture with routine healing |
| S52109E | Unspecified fracture of upper end of unspecified radius, subsequent encounter for open fracture type I or II with routine healing |
| S52109F | Unspecified fracture of upper end of unspecified radius, subsequent encounter for open fracture type IIIA, IIIB, or IIIC with routine healing |
| S52111D | Torus fracture of upper end of right radius, subsequent encounter for fracture with routine healing |
| S52112D | Torus fracture of upper end of left radius, subsequent encounter for fracture with routine healing |
| S52119D | Torus fracture of upper end of unspecified radius, subsequent encounter for fracture with routine healing |
| S52121D | Displaced fracture of head of right radius, subsequent encounter for closed fracture with routine healing |
| S52121E | Displaced fracture of head of right radius, subsequent encounter for open fracture type I or II with routine healing |
| S52121F | Displaced fracture of head of right radius, subsequent encounter for open fracture type IIIA, IIIB, or IIIC with routine healing |
| S52122D | Displaced fracture of head of left radius, subsequent encounter for closed fracture with routine healing |

| | |
|---|---|
| S52122E | Displaced fracture of head of left radius, subsequent encounter for open fracture type I or II with routine healing |
| S52122F | Displaced fracture of head of left radius, subsequent encounter for open fracture type IIIA, IIIB, or IIIC with routine healing |
| S52123D | Displaced fracture of head of unspecified radius, subsequent encounter for closed fracture with routine healing |
| S52123E | Displaced fracture of head of unspecified radius, subsequent encounter for open fracture type I or II with routine healing |
| S52123F | Displaced fracture of head of unspecified radius, subsequent encounter for open fracture type IIIA, IIIB, or IIIC with routine healing |
| S52124D | Nondisplaced fracture of head of right radius, subsequent encounter for closed fracture with routine healing |
| S52124E | Nondisplaced fracture of head of right radius, subsequent encounter for open fracture type I or II with routine healing |
| S52124F | Nondisplaced fracture of head of right radius, subsequent encounter for open fracture type IIIA, IIIB, or IIIC with routine healing |
| S52125D | Nondisplaced fracture of head of left radius, subsequent encounter for closed fracture with routine healing |
| S52125E | Nondisplaced fracture of head of left radius, subsequent encounter for open fracture type I or II with routine healing |
| S52125F | Nondisplaced fracture of head of left radius, subsequent encounter for open fracture type IIIA, IIIB, or IIIC with routine healing |
| S52126D | Nondisplaced fracture of head of unspecified radius, subsequent encounter for closed fracture with routine healing |
| S52126E | Nondisplaced fracture of head of unspecified radius, subsequent encounter for open fracture type I or II with routine healing |
| S52126F | Nondisplaced fracture of head of unspecified radius, subsequent encounter for open fracture type IIIA, IIIB, or IIIC with routine healing |
| S52131D | Displaced fracture of neck of right radius, subsequent encounter for closed fracture with routine healing |
| S52131E | Displaced fracture of neck of right radius, subsequent encounter for open fracture type I or II with routine healing |

| | |
|---|---|
| S52131F | Displaced fracture of neck of right radius, subsequent encounter for open fracture type IIIA, IIIB, or IIIC with routine healing |
| S52132D | Displaced fracture of neck of left radius, subsequent encounter for closed fracture with routine healing |
| S52132E | Displaced fracture of neck of left radius, subsequent encounter for open fracture type I or II with routine healing |
| S52132F | Displaced fracture of neck of left radius, subsequent encounter for open fracture type IIIA, IIIB, or IIIC with routine healing |
| S52133D | Displaced fracture of neck of unspecified radius, subsequent encounter for closed fracture with routine healing |
| S52133E | Displaced fracture of neck of unspecified radius, subsequent encounter for open fracture type I or II with routine healing |
| S52133F | Displaced fracture of neck of unspecified radius, subsequent encounter for open fracture type IIIA, IIIB, or IIIC with routine healing |
| S52134D | Nondisplaced fracture of neck of right radius, subsequent encounter for closed fracture with routine healing |
| S52134E | Nondisplaced fracture of neck of right radius, subsequent encounter for open fracture type I or II with routine healing |
| S52134F | Nondisplaced fracture of neck of right radius, subsequent encounter for open fracture type IIIA, IIIB, or IIIC with routine healing |
| S52135D | Nondisplaced fracture of neck of left radius, subsequent encounter for closed fracture with routine healing |
| S52135E | Nondisplaced fracture of neck of left radius, subsequent encounter for open fracture type I or II with routine healing |
| S52135F | Nondisplaced fracture of neck of left radius, subsequent encounter for open fracture type IIIA, IIIB, or IIIC with routine healing |
| S52136D | Nondisplaced fracture of neck of unspecified radius, subsequent encounter for closed fracture with routine healing |
| S52136E | Nondisplaced fracture of neck of unspecified radius, subsequent encounter for open fracture type I or II with routine healing |
| S52136F | Nondisplaced fracture of neck of unspecified radius, subsequent encounter for open fracture type IIIA, IIIB, or IIIC with routine healing |

| | |
|---|---|
| S52181D | Other fracture of upper end of right radius, subsequent encounter for closed fracture with routine healing |
| S52181E | Other fracture of upper end of right radius, subsequent encounter for open fracture type I or II with routine healing |
| S52181F | Other fracture of upper end of right radius, subsequent encounter for open fracture type IIIA, IIIB, or IIIC with routine healing |
| S52182D | Other fracture of upper end of left radius, subsequent encounter for closed fracture with routine healing |
| S52182E | Other fracture of upper end of left radius, subsequent encounter for open fracture type I or II with routine healing |
| S52182F | Other fracture of upper end of left radius, subsequent encounter for open fracture type IIIA, IIIB, or IIIC with routine healing |
| S52189D | Other fracture of upper end of unspecified radius, subsequent encounter for closed fracture with routine healing |
| S52189E | Other fracture of upper end of unspecified radius, subsequent encounter for open fracture type I or II with routine healing |
| S52189F | Other fracture of upper end of unspecified radius, subsequent encounter for open fracture type IIIA, IIIB, or IIIC with routine healing |
| S52201D | Unspecified fracture of shaft of right ulna, subsequent encounter for closed fracture with routine healing |
| S52201E | Unspecified fracture of shaft of right ulna, subsequent encounter for open fracture type I or II with routine healing |
| S52201F | Unspecified fracture of shaft of right ulna, subsequent encounter for open fracture type IIIA, IIIB, or IIIC with routine healing |
| S52202D | Unspecified fracture of shaft of left ulna, subsequent encounter for closed fracture with routine healing |
| S52202E | Unspecified fracture of shaft of left ulna, subsequent encounter for open fracture type I or II with routine healing |
| S52202F | Unspecified fracture of shaft of left ulna, subsequent encounter for open fracture type IIIA, IIIB, or IIIC with routine healing |
| S52209D | Unspecified fracture of shaft of unspecified ulna, subsequent encounter for closed fracture with routine healing |

| | |
|---|---|
| S52209E | Unspecified fracture of shaft of unspecified ulna, subsequent encounter for open fracture type I or II with routine healing |
| S52209F | Unspecified fracture of shaft of unspecified ulna, subsequent encounter for open fracture type IIIA, IIIB, or IIIC with routine healing |
| S52211D | Greenstick fracture of shaft of right ulna, subsequent encounter for fracture with routine healing |
| S52212D | Greenstick fracture of shaft of left ulna, subsequent encounter for fracture with routine healing |
| S52219D | Greenstick fracture of shaft of unspecified ulna, subsequent encounter for fracture with routine healing |
| S52221D | Displaced transverse fracture of shaft of right ulna, subsequent encounter for closed fracture with routine healing |
| S52221E | Displaced transverse fracture of shaft of right ulna, subsequent encounter for open fracture type I or II with routine healing |
| S52221F | Displaced transverse fracture of shaft of right ulna, subsequent encounter for open fracture type IIIA, IIIB, or IIIC with routine healing |
| S52222D | Displaced transverse fracture of shaft of left ulna, subsequent encounter for closed fracture with routine healing |
| S52222E | Displaced transverse fracture of shaft of left ulna, subsequent encounter for open fracture type I or II with routine healing |
| S52222F | Displaced transverse fracture of shaft of left ulna, subsequent encounter for open fracture type IIIA, IIIB, or IIIC with routine healing |
| S52223D | Displaced transverse fracture of shaft of unspecified ulna, subsequent encounter for closed fracture with routine healing |
| S52223E | Displaced transverse fracture of shaft of unspecified ulna, subsequent encounter for open fracture type I or II with routine healing |
| S52223F | Displaced transverse fracture of shaft of unspecified ulna, subsequent encounter for open fracture type IIIA, IIIB, or IIIC with routine healing |
| S52224D | Nondisplaced transverse fracture of shaft of right ulna, subsequent encounter for closed fracture with routine healing |
| S52224E | Nondisplaced transverse fracture of shaft of right ulna, subsequent encounter for open fracture type I or II with routine healing |

| | |
|---|---|
| S52224F | Nondisplaced transverse fracture of shaft of right ulna, subsequent encounter for open fracture type IIIA, IIIB, or IIIC with routine healing |
| S52225D | Nondisplaced transverse fracture of shaft of left ulna, subsequent encounter for closed fracture with routine healing |
| S52225E | Nondisplaced transverse fracture of shaft of left ulna, subsequent encounter for open fracture type I or II with routine healing |
| S52225F | Nondisplaced transverse fracture of shaft of left ulna, subsequent encounter for open fracture type IIIA, IIIB, or IIIC with routine healing |
| S52226D | Nondisplaced transverse fracture of shaft of unspecified ulna, subsequent encounter for closed fracture with routine healing |
| S52226E | Nondisplaced transverse fracture of shaft of unspecified ulna, subsequent encounter for open fracture type I or II with routine healing |
| S52226F | Nondisplaced transverse fracture of shaft of unspecified ulna, subsequent encounter for open fracture type IIIA, IIIB, or IIIC with routine healing |
| S52231D | Displaced oblique fracture of shaft of right ulna, subsequent encounter for closed fracture with routine healing |
| S52231E | Displaced oblique fracture of shaft of right ulna, subsequent encounter for open fracture type I or II with routine healing |
| S52231F | Displaced oblique fracture of shaft of right ulna, subsequent encounter for open fracture type IIIA, IIIB, or IIIC with routine healing |
| S52232D | Displaced oblique fracture of shaft of left ulna, subsequent encounter for closed fracture with routine healing |
| S52232E | Displaced oblique fracture of shaft of left ulna, subsequent encounter for open fracture type I or II with routine healing |
| S52232F | Displaced oblique fracture of shaft of left ulna, subsequent encounter for open fracture type IIIA, IIIB, or IIIC with routine healing |
| S52233D | Displaced oblique fracture of shaft of unspecified ulna, subsequent encounter for closed fracture with routine healing |
| S52233E | Displaced oblique fracture of shaft of unspecified ulna, subsequent encounter for open fracture type I or II with routine healing |
| S52233F | Displaced oblique fracture of shaft of unspecified ulna, subsequent encounter for open fracture type IIIA, IIIB, or IIIC with routine healing |

| | |
|---|---|
| S52234D | Nondisplaced oblique fracture of shaft of right ulna, subsequent encounter for closed fracture with routine healing |
| S52234E | Nondisplaced oblique fracture of shaft of right ulna, subsequent encounter for open fracture type I or II with routine healing |
| S52234F | Nondisplaced oblique fracture of shaft of right ulna, subsequent encounter for open fracture type IIIA, IIIB, or IIIC with routine healing |
| S52235D | Nondisplaced oblique fracture of shaft of left ulna, subsequent encounter for closed fracture with routine healing |
| S52235E | Nondisplaced oblique fracture of shaft of left ulna, subsequent encounter for open fracture type I or II with routine healing |
| S52235F | Nondisplaced oblique fracture of shaft of left ulna, subsequent encounter for open fracture type IIIA, IIIB, or IIIC with routine healing |
| S52236D | Nondisplaced oblique fracture of shaft of unspecified ulna, subsequent encounter for closed fracture with routine healing |
| S52236E | Nondisplaced oblique fracture of shaft of unspecified ulna, subsequent encounter for open fracture type I or II with routine healing |
| S52236F | Nondisplaced oblique fracture of shaft of unspecified ulna, subsequent encounter for open fracture type IIIA, IIIB, or IIIC with routine healing |
| S52241D | Displaced spiral fracture of shaft of ulna, right arm, subsequent encounter for closed fracture with routine healing |
| S52241E | Displaced spiral fracture of shaft of ulna, right arm, subsequent encounter for open fracture type I or II with routine healing |
| S52241F | Displaced spiral fracture of shaft of ulna, right arm, subsequent encounter for open fracture type IIIA, IIIB, or IIIC with routine healing |
| S52242D | Displaced spiral fracture of shaft of ulna, left arm, subsequent encounter for closed fracture with routine healing |
| S52242E | Displaced spiral fracture of shaft of ulna, left arm, subsequent encounter for open fracture type I or II with routine healing |
| S52242F | Displaced spiral fracture of shaft of ulna, left arm, subsequent encounter for open fracture type IIIA, IIIB, or IIIC with routine healing |
| S52243D | Displaced spiral fracture of shaft of ulna, unspecified arm, subsequent encounter for closed fracture with routine healing |

| | |
|---|---|
| S52243E | Displaced spiral fracture of shaft of ulna, unspecified arm, subsequent encounter for open fracture type I or II with routine healing |
| S52243F | Displaced spiral fracture of shaft of ulna, unspecified arm, subsequent encounter for open fracture type IIIA, IIIB, or IIIC with routine healing |
| S52244D | Nondisplaced spiral fracture of shaft of ulna, right arm, subsequent encounter for closed fracture with routine healing |
| S52244E | Nondisplaced spiral fracture of shaft of ulna, right arm, subsequent encounter for open fracture type I or II with routine healing |
| S52244F | Nondisplaced spiral fracture of shaft of ulna, right arm, subsequent encounter for open fracture type IIIA, IIIB, or IIIC with routine healing |
| S52245D | Nondisplaced spiral fracture of shaft of ulna, left arm, subsequent encounter for closed fracture with routine healing |
| S52245E | Nondisplaced spiral fracture of shaft of ulna, left arm, subsequent encounter for open fracture type I or II with routine healing |
| S52245F | Nondisplaced spiral fracture of shaft of ulna, left arm, subsequent encounter for open fracture type IIIA, IIIB, or IIIC with routine healing |
| S52246D | Nondisplaced spiral fracture of shaft of ulna, unspecified arm, subsequent encounter for closed fracture with routine healing |
| S52246E | Nondisplaced spiral fracture of shaft of ulna, unspecified arm, subsequent encounter for open fracture type I or II with routine healing |
| S52246F | Nondisplaced spiral fracture of shaft of ulna, unspecified arm, subsequent encounter for open fracture type IIIA, IIIB, or IIIC with routine healing |
| S52251D | Displaced comminuted fracture of shaft of ulna, right arm, subsequent encounter for closed fracture with routine healing |
| S52251E | Displaced comminuted fracture of shaft of ulna, right arm, subsequent encounter for open fracture type I or II with routine healing |
| S52251F | Displaced comminuted fracture of shaft of ulna, right arm, subsequent encounter for open fracture type IIIA, IIIB, or IIIC with routine healing |
| S52252D | Displaced comminuted fracture of shaft of ulna, left arm, subsequent encounter for closed fracture with routine healing |
| S52252E | Displaced comminuted fracture of shaft of ulna, left arm, subsequent encounter for open fracture type I or II with routine healing |

| | |
|---|---|
| S52252F | Displaced comminuted fracture of shaft of ulna, left arm, subsequent encounter for open fracture type IIIA, IIIB, or IIIC with routine healing |
| S52253D | Displaced comminuted fracture of shaft of ulna, unspecified arm, subsequent encounter for closed fracture with routine healing |
| S52253E | Displaced comminuted fracture of shaft of ulna, unspecified arm, subsequent encounter for open fracture type I or II with routine healing |
| S52253F | Displaced comminuted fracture of shaft of ulna, unspecified arm, subsequent encounter for open fracture type IIIA, IIIB, or IIIC with routine healing |
| S52254D | Nondisplaced comminuted fracture of shaft of ulna, right arm, subsequent encounter for closed fracture with routine healing |
| S52254E | Nondisplaced comminuted fracture of shaft of ulna, right arm, subsequent encounter for open fracture type I or II with routine healing |
| S52254F | Nondisplaced comminuted fracture of shaft of ulna, right arm, subsequent encounter for open fracture type IIIA, IIIB, or IIIC with routine healing |
| S52255D | Nondisplaced comminuted fracture of shaft of ulna, left arm, subsequent encounter for closed fracture with routine healing |
| S52255E | Nondisplaced comminuted fracture of shaft of ulna, left arm, subsequent encounter for open fracture type I or II with routine healing |
| S52255F | Nondisplaced comminuted fracture of shaft of ulna, left arm, subsequent encounter for open fracture type IIIA, IIIB, or IIIC with routine healing |
| S52256D | Nondisplaced comminuted fracture of shaft of ulna, unspecified arm, subsequent encounter for closed fracture with routine healing |
| S52256E | Nondisplaced comminuted fracture of shaft of ulna, unspecified arm, subsequent encounter for open fracture type I or II with routine healing |
| S52256F | Nondisplaced comminuted fracture of shaft of ulna, unspecified arm, subsequent encounter for open fracture type IIIA, IIIB, or IIIC with routine healing |
| S52261D | Displaced segmental fracture of shaft of ulna, right arm, subsequent encounter for closed fracture with routine healing |
| S52261E | Displaced segmental fracture of shaft of ulna, right arm, subsequent encounter for open fracture type I or II with routine healing |
| S52261F | Displaced segmental fracture of shaft of ulna, right arm, subsequent encounter for open fracture type IIIA, IIIB, or IIIC with routine healing |

| | |
|---|---|
| S52262D | Displaced segmental fracture of shaft of ulna, left arm, subsequent encounter for closed fracture with routine healing |
| S52262E | Displaced segmental fracture of shaft of ulna, left arm, subsequent encounter for open fracture type I or II with routine healing |
| S52262F | Displaced segmental fracture of shaft of ulna, left arm, subsequent encounter for open fracture type IIIA, IIIB, or IIIC with routine healing |
| S52263D | Displaced segmental fracture of shaft of ulna, unspecified arm, subsequent encounter for closed fracture with routine healing |
| S52263E | Displaced segmental fracture of shaft of ulna, unspecified arm, subsequent encounter for open fracture type I or II with routine healing |
| S52263F | Displaced segmental fracture of shaft of ulna, unspecified arm, subsequent encounter for open fracture type IIIA, IIIB, or IIIC with routine healing |
| S52264D | Nondisplaced segmental fracture of shaft of ulna, right arm, subsequent encounter for closed fracture with routine healing |
| S52264E | Nondisplaced segmental fracture of shaft of ulna, right arm, subsequent encounter for open fracture type I or II with routine healing |
| S52264F | Nondisplaced segmental fracture of shaft of ulna, right arm, subsequent encounter for open fracture type IIIA, IIIB, or IIIC with routine healing |
| S52265D | Nondisplaced segmental fracture of shaft of ulna, left arm, subsequent encounter for closed fracture with routine healing |
| S52265E | Nondisplaced segmental fracture of shaft of ulna, left arm, subsequent encounter for open fracture type I or II with routine healing |
| S52265F | Nondisplaced segmental fracture of shaft of ulna, left arm, subsequent encounter for open fracture type IIIA, IIIB, or IIIC with routine healing |
| S52266D | Nondisplaced segmental fracture of shaft of ulna, unspecified arm, subsequent encounter for closed fracture with routine healing |
| S52266E | Nondisplaced segmental fracture of shaft of ulna, unspecified arm, subsequent encounter for open fracture type I or II with routine healing |
| S52266F | Nondisplaced segmental fracture of shaft of ulna, unspecified arm, subsequent encounter for open fracture type IIIA, IIIB, or IIIC with routine healing |
| S52271D | Monteggias fracture of right ulna, subsequent encounter for closed fracture with routine healing |

| | |
|---|---|
| S52271E | Monteggias fracture of right ulna, subsequent encounter for open fracture type I or II with routine healing |
| S52271F | Monteggias fracture of right ulna, subsequent encounter for open fracture type IIIA, IIIB, or IIIC with routine healing |
| S52272D | Monteggias fracture of left ulna, subsequent encounter for closed fracture with routine healing |
| S52272E | Monteggias fracture of left ulna, subsequent encounter for open fracture type I or II with routine healing |
| S52272F | Monteggias fracture of left ulna, subsequent encounter for open fracture type IIIA, IIIB, or IIIC with routine healing |
| S52279D | Monteggias fracture of unspecified ulna, subsequent encounter for closed fracture with routine healing |
| S52279E | Monteggias fracture of unspecified ulna, subsequent encounter for open fracture type I or II with routine healing |
| S52279F | Monteggias fracture of unspecified ulna, subsequent encounter for open fracture type IIIA, IIIB, or IIIC with routine healing |
| S52281D | Bent bone of right ulna, subsequent encounter for closed fracture with routine healing |
| S52281E | Bent bone of right ulna, subsequent encounter for open fracture type I or II with routine healing |
| S52281F | Bent bone of right ulna, subsequent encounter for open fracture type IIIA, IIIB, or IIIC with routine healing |
| S52282D | Bent bone of left ulna, subsequent encounter for closed fracture with routine healing |
| S52282E | Bent bone of left ulna, subsequent encounter for open fracture type I or II with routine healing |
| S52282F | Bent bone of left ulna, subsequent encounter for open fracture type IIIA, IIIB, or IIIC with routine healing |
| S52283D | Bent bone of unspecified ulna, subsequent encounter for closed fracture with routine healing |
| S52283E | Bent bone of unspecified ulna, subsequent encounter for open fracture type I or II with routine healing |

| | |
|---|---|
| S52283F | Bent bone of unspecified ulna, subsequent encounter for open fracture type IIIA, IIIB, or IIIC with routine healing |
| S52291D | Other fracture of shaft of right ulna, subsequent encounter for closed fracture with routine healing |
| S52291E | Other fracture of shaft of right ulna, subsequent encounter for open fracture type I or II with routine healing |
| S52291F | Other fracture of shaft of right ulna, subsequent encounter for open fracture type IIIA, IIIB, or IIIC with routine healing |
| S52292D | Other fracture of shaft of left ulna, subsequent encounter for closed fracture with routine healing |
| S52292E | Other fracture of shaft of left ulna, subsequent encounter for open fracture type I or II with routine healing |
| S52292F | Other fracture of shaft of left ulna, subsequent encounter for open fracture type IIIA, IIIB, or IIIC with routine healing |
| S52299D | Other fracture of shaft of unspecified ulna, subsequent encounter for closed fracture with routine healing |
| S52299E | Other fracture of shaft of unspecified ulna, subsequent encounter for open fracture type I or II with routine healing |
| S52299F | Other fracture of shaft of unspecified ulna, subsequent encounter for open fracture type IIIA, IIIB, or IIIC with routine healing |
| S52301D | Unspecified fracture of shaft of right radius, subsequent encounter for closed fracture with routine healing |
| S52301E | Unspecified fracture of shaft of right radius, subsequent encounter for open fracture type I or II with routine healing |
| S52301F | Unspecified fracture of shaft of right radius, subsequent encounter for open fracture type IIIA, IIIB, or IIIC with routine healing |
| S52302D | Unspecified fracture of shaft of left radius, subsequent encounter for closed fracture with routine healing |
| S52302E | Unspecified fracture of shaft of left radius, subsequent encounter for open fracture type I or II with routine healing |
| S52302F | Unspecified fracture of shaft of left radius, subsequent encounter for open fracture type IIIA, IIIB, or IIIC with routine healing |

| | |
|---|---|
| S52309D | Unspecified fracture of shaft of unspecified radius, subsequent encounter for closed fracture with routine healing |
| S52309E | Unspecified fracture of shaft of unspecified radius, subsequent encounter for open fracture type I or II with routine healing |
| S52309F | Unspecified fracture of shaft of unspecified radius, subsequent encounter for open fracture type IIIA, IIIB, or IIIC with routine healing |
| S52311D | Greenstick fracture of shaft of radius, right arm, subsequent encounter for fracture with routine healing |
| S52312D | Greenstick fracture of shaft of radius, left arm, subsequent encounter for fracture with routine healing |
| S52319D | Greenstick fracture of shaft of radius, unspecified arm, subsequent encounter for fracture with routine healing |
| S52321D | Displaced transverse fracture of shaft of right radius, subsequent encounter for closed fracture with routine healing |
| S52321E | Displaced transverse fracture of shaft of right radius, subsequent encounter for open fracture type I or II with routine healing |
| S52321F | Displaced transverse fracture of shaft of right radius, subsequent encounter for open fracture type IIIA, IIIB, or IIIC with routine healing |
| S52322D | Displaced transverse fracture of shaft of left radius, subsequent encounter for closed fracture with routine healing |
| S52322E | Displaced transverse fracture of shaft of left radius, subsequent encounter for open fracture type I or II with routine healing |
| S52322F | Displaced transverse fracture of shaft of left radius, subsequent encounter for open fracture type IIIA, IIIB, or IIIC with routine healing |
| S52323D | Displaced transverse fracture of shaft of unspecified radius, subsequent encounter for closed fracture with routine healing |
| S52323E | Displaced transverse fracture of shaft of unspecified radius, subsequent encounter for open fracture type I or II with routine healing |
| S52323F | Displaced transverse fracture of shaft of unspecified radius, subsequent encounter for open fracture type IIIA, IIIB, or IIIC with routine healing |
| S52324D | Nondisplaced transverse fracture of shaft of right radius, subsequent encounter for closed fracture with routine healing |

| | |
|---|---|
| S52324E | Nondisplaced transverse fracture of shaft of right radius, subsequent encounter for open fracture type I or II with routine healing |
| S52324F | Nondisplaced transverse fracture of shaft of right radius, subsequent encounter for open fracture type IIIA, IIIB, or IIIC with routine healing |
| S52325D | Nondisplaced transverse fracture of shaft of left radius, subsequent encounter for closed fracture with routine healing |
| S52325E | Nondisplaced transverse fracture of shaft of left radius, subsequent encounter for open fracture type I or II with routine healing |
| S52325F | Nondisplaced transverse fracture of shaft of left radius, subsequent encounter for open fracture type IIIA, IIIB, or IIIC with routine healing |
| S52326D | Nondisplaced transverse fracture of shaft of unspecified radius, subsequent encounter for closed fracture with routine healing |
| S52326E | Nondisplaced transverse fracture of shaft of unspecified radius, subsequent encounter for open fracture type I or II with routine healing |
| S52326F | Nondisplaced transverse fracture of shaft of unspecified radius, subsequent encounter for open fracture type IIIA, IIIB, or IIIC with routine healing |
| S52331D | Displaced oblique fracture of shaft of right radius, subsequent encounter for closed fracture with routine healing |
| S52331E | Displaced oblique fracture of shaft of right radius, subsequent encounter for open fracture type I or II with routine healing |
| S52331F | Displaced oblique fracture of shaft of right radius, subsequent encounter for open fracture type IIIA, IIIB, or IIIC with routine healing |
| S52332D | Displaced oblique fracture of shaft of left radius, subsequent encounter for closed fracture with routine healing |
| S52332E | Displaced oblique fracture of shaft of left radius, subsequent encounter for open fracture type I or II with routine healing |
| S52332F | Displaced oblique fracture of shaft of left radius, subsequent encounter for open fracture type IIIA, IIIB, or IIIC with routine healing |
| S52333D | Displaced oblique fracture of shaft of unspecified radius, subsequent encounter for closed fracture with routine healing |
| S52333E | Displaced oblique fracture of shaft of unspecified radius, subsequent encounter for open fracture type I or II with routine healing |

| | |
|---|---|
| S52333F | Displaced oblique fracture of shaft of unspecified radius, subsequent encounter for open fracture type IIIA, IIIB, or IIIC with routine healing |
| S52334D | Nondisplaced oblique fracture of shaft of right radius, subsequent encounter for closed fracture with routine healing |
| S52334E | Nondisplaced oblique fracture of shaft of right radius, subsequent encounter for open fracture type I or II with routine healing |
| S52334F | Nondisplaced oblique fracture of shaft of right radius, subsequent encounter for open fracture type IIIA, IIIB, or IIIC with routine healing |
| S52335D | Nondisplaced oblique fracture of shaft of left radius, subsequent encounter for closed fracture with routine healing |
| S52335E | Nondisplaced oblique fracture of shaft of left radius, subsequent encounter for open fracture type I or II with routine healing |
| S52335F | Nondisplaced oblique fracture of shaft of left radius, subsequent encounter for open fracture type IIIA, IIIB, or IIIC with routine healing |
| S52336D | Nondisplaced oblique fracture of shaft of unspecified radius, subsequent encounter for closed fracture with routine healing |
| S52336E | Nondisplaced oblique fracture of shaft of unspecified radius, subsequent encounter for open fracture type I or II with routine healing |
| S52336F | Nondisplaced oblique fracture of shaft of unspecified radius, subsequent encounter for open fracture type IIIA, IIIB, or IIIC with routine healing |
| S52341D | Displaced spiral fracture of shaft of radius, right arm, subsequent encounter for closed fracture with routine healing |
| S52341E | Displaced spiral fracture of shaft of radius, right arm, subsequent encounter for open fracture type I or II with routine healing |
| S52341F | Displaced spiral fracture of shaft of radius, right arm, subsequent encounter for open fracture type IIIA, IIIB, or IIIC with routine healing |
| S52342D | Displaced spiral fracture of shaft of radius, left arm, subsequent encounter for closed fracture with routine healing |
| S52342E | Displaced spiral fracture of shaft of radius, left arm, subsequent encounter for open fracture type I or II with routine healing |
| S52342F | Displaced spiral fracture of shaft of radius, left arm, subsequent encounter for open fracture type IIIA, IIIB, or IIIC with routine healing |

| | |
|---|---|
| S52343D | Displaced spiral fracture of shaft of radius, unspecified arm, subsequent encounter for closed fracture with routine healing |
| S52343E | Displaced spiral fracture of shaft of radius, unspecified arm, subsequent encounter for open fracture type I or II with routine healing |
| S52343F | Displaced spiral fracture of shaft of radius, unspecified arm, subsequent encounter for open fracture type IIIA, IIIB, or IIIC with routine healing |
| S52344D | Nondisplaced spiral fracture of shaft of radius, right arm, subsequent encounter for closed fracture with routine healing |
| S52344E | Nondisplaced spiral fracture of shaft of radius, right arm, subsequent encounter for open fracture type I or II with routine healing |
| S52344F | Nondisplaced spiral fracture of shaft of radius, right arm, subsequent encounter for open fracture type IIIA, IIIB, or IIIC with routine healing |
| S52345D | Nondisplaced spiral fracture of shaft of radius, left arm, subsequent encounter for closed fracture with routine healing |
| S52345E | Nondisplaced spiral fracture of shaft of radius, left arm, subsequent encounter for open fracture type I or II with routine healing |
| S52345F | Nondisplaced spiral fracture of shaft of radius, left arm, subsequent encounter for open fracture type IIIA, IIIB, or IIIC with routine healing |
| S52346D | Nondisplaced spiral fracture of shaft of radius, unspecified arm, subsequent encounter for closed fracture with routine healing |
| S52346E | Nondisplaced spiral fracture of shaft of radius, unspecified arm, subsequent encounter for open fracture type I or II with routine healing |
| S52346F | Nondisplaced spiral fracture of shaft of radius, unspecified arm, subsequent encounter for open fracture type IIIA, IIIB, or IIIC with routine healing |
| S52351D | Displaced comminuted fracture of shaft of radius, right arm, subsequent encounter for closed fracture with routine healing |
| S52351E | Displaced comminuted fracture of shaft of radius, right arm, subsequent encounter for open fracture type I or II with routine healing |
| S52351F | Displaced comminuted fracture of shaft of radius, right arm, subsequent encounter for open fracture type IIIA, IIIB, or IIIC with routine healing |
| S52352D | Displaced comminuted fracture of shaft of radius, left arm, subsequent encounter for closed fracture with routine healing |

| | |
|---|---|
| S52352E | Displaced comminuted fracture of shaft of radius, left arm, subsequent encounter for open fracture type I or II with routine healing |
| S52352F | Displaced comminuted fracture of shaft of radius, left arm, subsequent encounter for open fracture type IIIA, IIIB, or IIIC with routine healing |
| S52353D | Displaced comminuted fracture of shaft of radius, unspecified arm, subsequent encounter for closed fracture with routine healing |
| S52353E | Displaced comminuted fracture of shaft of radius, unspecified arm, subsequent encounter for open fracture type I or II with routine healing |
| S52353F | Displaced comminuted fracture of shaft of radius, unspecified arm, subsequent encounter for open fracture type IIIA, IIIB, or IIIC with routine healing |
| S52354D | Nondisplaced comminuted fracture of shaft of radius, right arm, subsequent encounter for closed fracture with routine healing |
| S52354E | Nondisplaced comminuted fracture of shaft of radius, right arm, subsequent encounter for open fracture type I or II with routine healing |
| S52354F | Nondisplaced comminuted fracture of shaft of radius, right arm, subsequent encounter for open fracture type IIIA, IIIB, or IIIC with routine healing |
| S52355D | Nondisplaced comminuted fracture of shaft of radius, left arm, subsequent encounter for closed fracture with routine healing |
| S52355E | Nondisplaced comminuted fracture of shaft of radius, left arm, subsequent encounter for open fracture type I or II with routine healing |
| S52355F | Nondisplaced comminuted fracture of shaft of radius, left arm, subsequent encounter for open fracture type IIIA, IIIB, or IIIC with routine healing |
| S52356D | Nondisplaced comminuted fracture of shaft of radius, unspecified arm, subsequent encounter for closed fracture with routine healing |
| S52356E | Nondisplaced comminuted fracture of shaft of radius, unspecified arm, subsequent encounter for open fracture type I or II with routine healing |
| S52356F | Nondisplaced comminuted fracture of shaft of radius, unspecified arm, subsequent encounter for open fracture type IIIA, IIIB, or IIIC with routine healing |
| S52361D | Displaced segmental fracture of shaft of radius, right arm, subsequent encounter for closed fracture with routine healing |
| S52361E | Displaced segmental fracture of shaft of radius, right arm, subsequent encounter for open fracture type I or II with routine healing |

| | |
|---|---|
| S52361F | Displaced segmental fracture of shaft of radius, right arm, subsequent encounter for open fracture type IIIA, IIIB, or IIIC with routine healing |
| S52362D | Displaced segmental fracture of shaft of radius, left arm, subsequent encounter for closed fracture with routine healing |
| S52362E | Displaced segmental fracture of shaft of radius, left arm, subsequent encounter for open fracture type I or II with routine healing |
| S52362F | Displaced segmental fracture of shaft of radius, left arm, subsequent encounter for open fracture type IIIA, IIIB, or IIIC with routine healing |
| S52363D | Displaced segmental fracture of shaft of radius, unspecified arm, subsequent encounter for closed fracture with routine healing |
| S52363E | Displaced segmental fracture of shaft of radius, unspecified arm, subsequent encounter for open fracture type I or II with routine healing |
| S52363F | Displaced segmental fracture of shaft of radius, unspecified arm, subsequent encounter for open fracture type IIIA, IIIB, or IIIC with routine healing |
| S52364D | Nondisplaced segmental fracture of shaft of radius, right arm, subsequent encounter for closed fracture with routine healing |
| S52364E | Nondisplaced segmental fracture of shaft of radius, right arm, subsequent encounter for open fracture type I or II with routine healing |
| S52364F | Nondisplaced segmental fracture of shaft of radius, right arm, subsequent encounter for open fracture type IIIA, IIIB, or IIIC with routine healing |
| S52365D | Nondisplaced segmental fracture of shaft of radius, left arm, subsequent encounter for closed fracture with routine healing |
| S52365E | Nondisplaced segmental fracture of shaft of radius, left arm, subsequent encounter for open fracture type I or II with routine healing |
| S52365F | Nondisplaced segmental fracture of shaft of radius, left arm, subsequent encounter for open fracture type IIIA, IIIB, or IIIC with routine healing |
| S52366D | Nondisplaced segmental fracture of shaft of radius, unspecified arm, subsequent encounter for closed fracture with routine healing |
| S52366E | Nondisplaced segmental fracture of shaft of radius, unspecified arm, subsequent encounter for open fracture type I or II with routine healing |
| S52366F | Nondisplaced segmental fracture of shaft of radius, unspecified arm, subsequent encounter for open fracture type IIIA, IIIB, or IIIC with routine healing |

| | |
|---|---|
| S52371D | Galeazzis fracture of right radius, subsequent encounter for closed fracture with routine healing |
| S52371E | Galeazzis fracture of right radius, subsequent encounter for open fracture type I or II with routine healing |
| S52371F | Galeazzis fracture of right radius, subsequent encounter for open fracture type IIIA, IIIB, or IIIC with routine healing |
| S52372D | Galeazzis fracture of left radius, subsequent encounter for closed fracture with routine healing |
| S52372E | Galeazzis fracture of left radius, subsequent encounter for open fracture type I or II with routine healing |
| S52372F | Galeazzis fracture of left radius, subsequent encounter for open fracture type IIIA, IIIB, or IIIC with routine healing |
| S52379D | Galeazzis fracture of unspecified radius, subsequent encounter for closed fracture with routine healing |
| S52379E | Galeazzis fracture of unspecified radius, subsequent encounter for open fracture type I or II with routine healing |
| S52379F | Galeazzis fracture of unspecified radius, subsequent encounter for open fracture type IIIA, IIIB, or IIIC with routine healing |
| S52381D | Bent bone of right radius, subsequent encounter for closed fracture with routine healing |
| S52381E | Bent bone of right radius, subsequent encounter for open fracture type I or II with routine healing |
| S52381F | Bent bone of right radius, subsequent encounter for open fracture type IIIA, IIIB, or IIIC with routine healing |
| S52382D | Bent bone of left radius, subsequent encounter for closed fracture with routine healing |
| S52382E | Bent bone of left radius, subsequent encounter for open fracture type I or II with routine healing |
| S52382F | Bent bone of left radius, subsequent encounter for open fracture type IIIA, IIIB, or IIIC with routine healing |
| S52389D | Bent bone of unspecified radius, subsequent encounter for closed fracture with routine healing |

| | |
|---|---|
| S52389E | Bent bone of unspecified radius, subsequent encounter for open fracture type I or II with routine healing |
| S52389F | Bent bone of unspecified radius, subsequent encounter for open fracture type IIIA, IIIB, or IIIC with routine healing |
| S52391D | Other fracture of shaft of radius, right arm, subsequent encounter for closed fracture with routine healing |
| S52391E | Other fracture of shaft of radius, right arm, subsequent encounter for open fracture type I or II with routine healing |
| S52391F | Other fracture of shaft of radius, right arm, subsequent encounter for open fracture type IIIA, IIIB, or IIIC with routine healing |
| S52392D | Other fracture of shaft of radius, left arm, subsequent encounter for closed fracture with routine healing |
| S52392E | Other fracture of shaft of radius, left arm, subsequent encounter for open fracture type I or II with routine healing |
| S52392F | Other fracture of shaft of radius, left arm, subsequent encounter for open fracture type IIIA, IIIB, or IIIC with routine healing |
| S52399D | Other fracture of shaft of radius, unspecified arm, subsequent encounter for closed fracture with routine healing |
| S52399E | Other fracture of shaft of radius, unspecified arm, subsequent encounter for open fracture type I or II with routine healing |
| S52399F | Other fracture of shaft of radius, unspecified arm, subsequent encounter for open fracture type IIIA, IIIB, or IIIC with routine healing |
| S52501D | Unspecified fracture of the lower end of right radius, subsequent encounter for closed fracture with routine healing |
| S52501E | Unspecified fracture of the lower end of right radius, subsequent encounter for open fracture type I or II with routine healing |
| S52501F | Unspecified fracture of the lower end of right radius, subsequent encounter for open fracture type IIIA, IIIB, or IIIC with routine healing |
| S52502D | Unspecified fracture of the lower end of left radius, subsequent encounter for closed fracture with routine healing |
| S52502E | Unspecified fracture of the lower end of left radius, subsequent encounter for open fracture type I or II with routine healing |

| | |
|---|---|
| S52502F | Unspecified fracture of the lower end of left radius, subsequent encounter for open fracture type IIIA, IIIB, or IIIC with routine healing |
| S52509D | Unspecified fracture of the lower end of unspecified radius, subsequent encounter for closed fracture with routine healing |
| S52509E | Unspecified fracture of the lower end of unspecified radius, subsequent encounter for open fracture type I or II with routine healing |
| S52509F | Unspecified fracture of the lower end of unspecified radius, subsequent encounter for open fracture type IIIA, IIIB, or IIIC with routine healing |
| S52511D | Displaced fracture of right radial styloid process, subsequent encounter for closed fracture with routine healing |
| S52511E | Displaced fracture of right radial styloid process, subsequent encounter for open fracture type I or II with routine healing |
| S52511F | Displaced fracture of right radial styloid process, subsequent encounter for open fracture type IIIA, IIIB, or IIIC with routine healing |
| S52512D | Displaced fracture of left radial styloid process, subsequent encounter for closed fracture with routine healing |
| S52512E | Displaced fracture of left radial styloid process, subsequent encounter for open fracture type I or II with routine healing |
| S52512F | Displaced fracture of left radial styloid process, subsequent encounter for open fracture type IIIA, IIIB, or IIIC with routine healing |
| S52513D | Displaced fracture of unspecified radial styloid process, subsequent encounter for closed fracture with routine healing |
| S52513E | Displaced fracture of unspecified radial styloid process, subsequent encounter for open fracture type I or II with routine healing |
| S52513F | Displaced fracture of unspecified radial styloid process, subsequent encounter for open fracture type IIIA, IIIB, or IIIC with routine healing |
| S52514D | Nondisplaced fracture of right radial styloid process, subsequent encounter for closed fracture with routine healing |
| S52514E | Nondisplaced fracture of right radial styloid process, subsequent encounter for open fracture type I or II with routine healing |
| S52514F | Nondisplaced fracture of right radial styloid process, subsequent encounter for open fracture type IIIA, IIIB, or IIIC with routine healing |

| | |
|---|---|
| S52515D | Nondisplaced fracture of left radial styloid process, subsequent encounter for closed fracture with routine healing |
| S52515E | Nondisplaced fracture of left radial styloid process, subsequent encounter for open fracture type I or II with routine healing |
| S52515F | Nondisplaced fracture of left radial styloid process, subsequent encounter for open fracture type IIIA, IIIB, or IIIC with routine healing |
| S52516D | Nondisplaced fracture of unspecified radial styloid process, subsequent encounter for closed fracture with routine healing |
| S52516E | Nondisplaced fracture of unspecified radial styloid process, subsequent encounter for open fracture type I or II with routine healing |
| S52516F | Nondisplaced fracture of unspecified radial styloid process, subsequent encounter for open fracture type IIIA, IIIB, or IIIC with routine healing |
| S52521D | Torus fracture of lower end of right radius, subsequent encounter for fracture with routine healing |
| S52522D | Torus fracture of lower end of left radius, subsequent encounter for fracture with routine healing |
| S52529D | Torus fracture of lower end of unspecified radius, subsequent encounter for fracture with routine healing |
| S52531D | Colles fracture of right radius, subsequent encounter for closed fracture with routine healing |
| S52531E | Colles fracture of right radius, subsequent encounter for open fracture type I or II with routine healing |
| S52531F | Colles fracture of right radius, subsequent encounter for open fracture type IIIA, IIIB, or IIIC with routine healing |
| S52532D | Colles fracture of left radius, subsequent encounter for closed fracture with routine healing |
| S52532E | Colles fracture of left radius, subsequent encounter for open fracture type I or II with routine healing |
| S52532F | Colles fracture of left radius, subsequent encounter for open fracture type IIIA, IIIB, or IIIC with routine healing |
| S52539D | Colles fracture of unspecified radius, subsequent encounter for closed fracture with routine healing |

| | |
|---|---|
| S52539E | Colles fracture of unspecified radius, subsequent encounter for open fracture type I or II with routine healing |
| S52539F | Colles fracture of unspecified radius, subsequent encounter for open fracture type IIIA, IIIB, or IIIC with routine healing |
| S52541D | Smiths fracture of right radius, subsequent encounter for closed fracture with routine healing |
| S52541E | Smiths fracture of right radius, subsequent encounter for open fracture type I or II with routine healing |
| S52541F | Smiths fracture of right radius, subsequent encounter for open fracture type IIIA, IIIB, or IIIC with routine healing |
| S52542D | Smiths fracture of left radius, subsequent encounter for closed fracture with routine healing |
| S52542E | Smiths fracture of left radius, subsequent encounter for open fracture type I or II with routine healing |
| S52542F | Smiths fracture of left radius, subsequent encounter for open fracture type IIIA, IIIB, or IIIC with routine healing |
| S52549D | Smiths fracture of unspecified radius, subsequent encounter for closed fracture with routine healing |
| S52549E | Smiths fracture of unspecified radius, subsequent encounter for open fracture type I or II with routine healing |
| S52549F | Smiths fracture of unspecified radius, subsequent encounter for open fracture type IIIA, IIIB, or IIIC with routine healing |
| S52551D | Other extraarticular fracture of lower end of right radius, subsequent encounter for closed fracture with routine healing |
| S52551E | Other extraarticular fracture of lower end of right radius, subsequent encounter for open fracture type I or II with routine healing |
| S52551F | Other extraarticular fracture of lower end of right radius, subsequent encounter for open fracture type IIIA, IIIB, or IIIC with routine healing |
| S52552D | Other extraarticular fracture of lower end of left radius, subsequent encounter for closed fracture with routine healing |
| S52552E | Other extraarticular fracture of lower end of left radius, subsequent encounter for open fracture type I or II with routine healing |

| | |
|---|---|
| S52552F | Other extraarticular fracture of lower end of left radius, subsequent encounter for open fracture type IIIA, IIIB, or IIIC with routine healing |
| S52559D | Other extraarticular fracture of lower end of unspecified radius, subsequent encounter for closed fracture with routine healing |
| S52559E | Other extraarticular fracture of lower end of unspecified radius, subsequent encounter for open fracture type I or II with routine healing |
| S52559F | Other extraarticular fracture of lower end of unspecified radius, subsequent encounter for open fracture type IIIA, IIIB, or IIIC with routine healing |
| S52561D | Bartons fracture of right radius, subsequent encounter for closed fracture with routine healing |
| S52561E | Bartons fracture of right radius, subsequent encounter for open fracture type I or II with routine healing |
| S52561F | Bartons fracture of right radius, subsequent encounter for open fracture type IIIA, IIIB, or IIIC with routine healing |
| S52562D | Bartons fracture of left radius, subsequent encounter for closed fracture with routine healing |
| S52562E | Bartons fracture of left radius, subsequent encounter for open fracture type I or II with routine healing |
| S52562F | Bartons fracture of left radius, subsequent encounter for open fracture type IIIA, IIIB, or IIIC with routine healing |
| S52569D | Bartons fracture of unspecified radius, subsequent encounter for closed fracture with routine healing |
| S52569E | Bartons fracture of unspecified radius, subsequent encounter for open fracture type I or II with routine healing |
| S52569F | Bartons fracture of unspecified radius, subsequent encounter for open fracture type IIIA, IIIB, or IIIC with routine healing |
| S52571D | Other intraarticular fracture of lower end of right radius, subsequent encounter for closed fracture with routine healing |
| S52571E | Other intraarticular fracture of lower end of right radius, subsequent encounter for open fracture type I or II with routine healing |
| S52571F | Other intraarticular fracture of lower end of right radius, subsequent encounter for open fracture type IIIA, IIIB, or IIIC with routine healing |

| | |
|---|---|
| S52572D | Other intraarticular fracture of lower end of left radius, subsequent encounter for closed fracture with routine healing |
| S52572E | Other intraarticular fracture of lower end of left radius, subsequent encounter for open fracture type I or II with routine healing |
| S52572F | Other intraarticular fracture of lower end of left radius, subsequent encounter for open fracture type IIIA, IIIB, or IIIC with routine healing |
| S52579D | Other intraarticular fracture of lower end of unspecified radius, subsequent encounter for closed fracture with routine healing |
| S52579E | Other intraarticular fracture of lower end of unspecified radius, subsequent encounter for open fracture type I or II with routine healing |
| S52579F | Other intraarticular fracture of lower end of unspecified radius, subsequent encounter for open fracture type IIIA, IIIB, or IIIC with routine healing |
| S52591D | Other fractures of lower end of right radius, subsequent encounter for closed fracture with routine healing |
| S52591E | Other fractures of lower end of right radius, subsequent encounter for open fracture type I or II with routine healing |
| S52591F | Other fractures of lower end of right radius, subsequent encounter for open fracture type IIIA, IIIB, or IIIC with routine healing |
| S52592D | Other fractures of lower end of left radius, subsequent encounter for closed fracture with routine healing |
| S52592E | Other fractures of lower end of left radius, subsequent encounter for open fracture type I or II with routine healing |
| S52592F | Other fractures of lower end of left radius, subsequent encounter for open fracture type IIIA, IIIB, or IIIC with routine healing |
| S52599D | Other fractures of lower end of unspecified radius, subsequent encounter for closed fracture with routine healing |
| S52599E | Other fractures of lower end of unspecified radius, subsequent encounter for open fracture type I or II with routine healing |
| S52599F | Other fractures of lower end of unspecified radius, subsequent encounter for open fracture type IIIA, IIIB, or IIIC with routine healing |
| S52601D | Unspecified fracture of lower end of right ulna, subsequent encounter for closed fracture with routine healing |

| | |
|---|---|
| S52601E | Unspecified fracture of lower end of right ulna, subsequent encounter for open fracture type I or II with routine healing |
| S52601F | Unspecified fracture of lower end of right ulna, subsequent encounter for open fracture type IIIA, IIIB, or IIIC with routine healing |
| S52602D | Unspecified fracture of lower end of left ulna, subsequent encounter for closed fracture with routine healing |
| S52602E | Unspecified fracture of lower end of left ulna, subsequent encounter for open fracture type I or II with routine healing |
| S52602F | Unspecified fracture of lower end of left ulna, subsequent encounter for open fracture type IIIA, IIIB, or IIIC with routine healing |
| S52609D | Unspecified fracture of lower end of unspecified ulna, subsequent encounter for closed fracture with routine healing |
| S52609E | Unspecified fracture of lower end of unspecified ulna, subsequent encounter for open fracture type I or II with routine healing |
| S52609F | Unspecified fracture of lower end of unspecified ulna, subsequent encounter for open fracture type IIIA, IIIB, or IIIC with routine healing |
| S52611D | Displaced fracture of right ulna styloid process, subsequent encounter for closed fracture with routine healing |
| S52611E | Displaced fracture of right ulna styloid process, subsequent encounter for open fracture type I or II with routine healing |
| S52611F | Displaced fracture of right ulna styloid process, subsequent encounter for open fracture type IIIA, IIIB, or IIIC with routine healing |
| S52612D | Displaced fracture of left ulna styloid process, subsequent encounter for closed fracture with routine healing |
| S52612E | Displaced fracture of left ulna styloid process, subsequent encounter for open fracture type I or II with routine healing |
| S52612F | Displaced fracture of left ulna styloid process, subsequent encounter for open fracture type IIIA, IIIB, or IIIC with routine healing |
| S52613D | Displaced fracture of unspecified ulna styloid process, subsequent encounter for closed fracture with routine healing |
| S52613E | Displaced fracture of unspecified ulna styloid process, subsequent encounter for open fracture type I or II with routine healing |

| | |
|---|---|
| S52613F | Displaced fracture of unspecified ulna styloid process, subsequent encounter for open fracture type IIIA, IIIB, or IIIC with routine healing |
| S52614D | Nondisplaced fracture of right ulna styloid process, subsequent encounter for closed fracture with routine healing |
| S52614E | Nondisplaced fracture of right ulna styloid process, subsequent encounter for open fracture type I or II with routine healing |
| S52614F | Nondisplaced fracture of right ulna styloid process, subsequent encounter for open fracture type IIIA, IIIB, or IIIC with routine healing |
| S52615D | Nondisplaced fracture of left ulna styloid process, subsequent encounter for closed fracture with routine healing |
| S52615E | Nondisplaced fracture of left ulna styloid process, subsequent encounter for open fracture type I or II with routine healing |
| S52615F | Nondisplaced fracture of left ulna styloid process, subsequent encounter for open fracture type IIIA, IIIB, or IIIC with routine healing |
| S52616D | Nondisplaced fracture of unspecified ulna styloid process, subsequent encounter for closed fracture with routine healing |
| S52616E | Nondisplaced fracture of unspecified ulna styloid process, subsequent encounter for open fracture type I or II with routine healing |
| S52616F | Nondisplaced fracture of unspecified ulna styloid process, subsequent encounter for open fracture type IIIA, IIIB, or IIIC with routine healing |
| S52621D | Torus fracture of lower end of right ulna, subsequent encounter for fracture with routine healing |
| S52622D | Torus fracture of lower end of left ulna, subsequent encounter for fracture with routine healing |
| S52629D | Torus fracture of lower end of unspecified ulna, subsequent encounter for fracture with routine healing |
| S52691D | Other fracture of lower end of right ulna, subsequent encounter for closed fracture with routine healing |
| S52691E | Other fracture of lower end of right ulna, subsequent encounter for open fracture type I or II with routine healing |
| S52691F | Other fracture of lower end of right ulna, subsequent encounter for open fracture type IIIA, IIIB, or IIIC with routine healing |

| | |
|---|---|
| S52692D | Other fracture of lower end of left ulna, subsequent encounter for closed fracture with routine healing |
| S52692E | Other fracture of lower end of left ulna, subsequent encounter for open fracture type I or II with routine healing |
| S52692F | Other fracture of lower end of left ulna, subsequent encounter for open fracture type IIIA, IIIB, or IIIC with routine healing |
| S52699D | Other fracture of lower end of unspecified ulna, subsequent encounter for closed fracture with routine healing |
| S52699E | Other fracture of lower end of unspecified ulna, subsequent encounter for open fracture type I or II with routine healing |
| S52699F | Other fracture of lower end of unspecified ulna, subsequent encounter for open fracture type IIIA, IIIB, or IIIC with routine healing |
| S5290XD | Unspecified fracture of unspecified forearm, subsequent encounter for closed fracture with routine healing |
| S5290XE | Unspecified fracture of unspecified forearm, subsequent encounter for open fracture type I or II with routine healing |
| S5290XF | Unspecified fracture of unspecified forearm, subsequent encounter for open fracture type IIIA, IIIB, or IIIC with routine healing |
| S5291XD | Unspecified fracture of right forearm, subsequent encounter for closed fracture with routine healing |
| S5291XE | Unspecified fracture of right forearm, subsequent encounter for open fracture type I or II with routine healing |
| S5291XF | Unspecified fracture of right forearm, subsequent encounter for open fracture type IIIA, IIIB, or IIIC with routine healing |
| S5292XD | Unspecified fracture of left forearm, subsequent encounter for closed fracture with routine healing |
| S5292XE | Unspecified fracture of left forearm, subsequent encounter for open fracture type I or II with routine healing |
| S5292XF | Unspecified fracture of left forearm, subsequent encounter for open fracture type IIIA, IIIB, or IIIC with routine healing |
| S59001D | Unspecified physeal fracture of lower end of ulna, right arm, subsequent encounter for fracture with routine healing |

| | |
|---|---|
| S59002D | Unspecified physeal fracture of lower end of ulna, left arm, subsequent encounter for fracture with routine healing |
| S59009D | Unspecified physeal fracture of lower end of ulna, unspecified arm, subsequent encounter for fracture with routine healing |
| S59011D | Salter-Harris Type I physeal fracture of lower end of ulna, right arm, subsequent encounter for fracture with routine healing |
| S59012D | Salter-Harris Type I physeal fracture of lower end of ulna, left arm, subsequent encounter for fracture with routine healing |
| S59019D | Salter-Harris Type I physeal fracture of lower end of ulna, unspecified arm, subsequent encounter for fracture with routine healing |
| S59021D | Salter-Harris Type II physeal fracture of lower end of ulna, right arm, subsequent encounter for fracture with routine healing |
| S59022D | Salter-Harris Type II physeal fracture of lower end of ulna, left arm, subsequent encounter for fracture with routine healing |
| S59029D | Salter-Harris Type II physeal fracture of lower end of ulna, unspecified arm, subsequent encounter for fracture with routine healing |
| S59031D | Salter-Harris Type III physeal fracture of lower end of ulna, right arm, subsequent encounter for fracture with routine healing |
| S59032D | Salter-Harris Type III physeal fracture of lower end of ulna, left arm, subsequent encounter for fracture with routine healing |
| S59039D | Salter-Harris Type III physeal fracture of lower end of ulna, unspecified arm, subsequent encounter for fracture with routine healing |
| S59041D | Salter-Harris Type IV physeal fracture of lower end of ulna, right arm, subsequent encounter for fracture with routine healing |
| S59042D | Salter-Harris Type IV physeal fracture of lower end of ulna, left arm, subsequent encounter for fracture with routine healing |
| S59049D | Salter-Harris Type IV physeal fracture of lower end of ulna, unspecified arm, subsequent encounter for fracture with routine healing |
| S59091D | Other physeal fracture of lower end of ulna, right arm, subsequent encounter for fracture with routine healing |
| S59092D | Other physeal fracture of lower end of ulna, left arm, subsequent encounter for fracture with routine healing |

| | |
|---|---|
| S59099D | Other physeal fracture of lower end of ulna, unspecified arm, subsequent encounter for fracture with routine healing |
| S59101D | Unspecified physeal fracture of upper end of radius, right arm, subsequent encounter for fracture with routine healing |
| S59102D | Unspecified physeal fracture of upper end of radius, left arm, subsequent encounter for fracture with routine healing |
| S59109D | Unspecified physeal fracture of upper end of radius, unspecified arm, subsequent encounter for fracture with routine healing |
| S59111D | Salter-Harris Type I physeal fracture of upper end of radius, right arm, subsequent encounter for fracture with routine healing |
| S59112D | Salter-Harris Type I physeal fracture of upper end of radius, left arm, subsequent encounter for fracture with routine healing |
| S59119D | Salter-Harris Type I physeal fracture of upper end of radius, unspecified arm, subsequent encounter for fracture with routine healing |
| S59121D | Salter-Harris Type II physeal fracture of upper end of radius, right arm, subsequent encounter for fracture with routine healing |
| S59122D | Salter-Harris Type II physeal fracture of upper end of radius, left arm, subsequent encounter for fracture with routine healing |
| S59129D | Salter-Harris Type II physeal fracture of upper end of radius, unspecified arm, subsequent encounter for fracture with routine healing |
| S59131D | Salter-Harris Type III physeal fracture of upper end of radius, right arm, subsequent encounter for fracture with routine healing |
| S59132D | Salter-Harris Type III physeal fracture of upper end of radius, left arm, subsequent encounter for fracture with routine healing |
| S59139D | Salter-Harris Type III physeal fracture of upper end of radius, unspecified arm, subsequent encounter for fracture with routine healing |
| S59141D | Salter-Harris Type IV physeal fracture of upper end of radius, right arm, subsequent encounter for fracture with routine healing |
| S59142D | Salter-Harris Type IV physeal fracture of upper end of radius, left arm, subsequent encounter for fracture with routine healing |
| S59149D | Salter-Harris Type IV physeal fracture of upper end of radius, unspecified arm, subsequent encounter for fracture with routine healing |

| | |
|---|---|
| S59191D | Other physeal fracture of upper end of radius, right arm, subsequent encounter for fracture with routine healing |
| S59192D | Other physeal fracture of upper end of radius, left arm, subsequent encounter for fracture with routine healing |
| S59199D | Other physeal fracture of upper end of radius, unspecified arm, subsequent encounter for fracture with routine healing |
| S59201D | Unspecified physeal fracture of lower end of radius, right arm, subsequent encounter for fracture with routine healing |
| S59202D | Unspecified physeal fracture of lower end of radius, left arm, subsequent encounter for fracture with routine healing |
| S59209D | Unspecified physeal fracture of lower end of radius, unspecified arm, subsequent encounter for fracture with routine healing |
| S59211D | Salter-Harris Type I physeal fracture of lower end of radius, right arm, subsequent encounter for fracture with routine healing |
| S59212D | Salter-Harris Type I physeal fracture of lower end of radius, left arm, subsequent encounter for fracture with routine healing |
| S59219D | Salter-Harris Type I physeal fracture of lower end of radius, unspecified arm, subsequent encounter for fracture with routine healing |
| S59221D | Salter-Harris Type II physeal fracture of lower end of radius, right arm, subsequent encounter for fracture with routine healing |
| S59222D | Salter-Harris Type II physeal fracture of lower end of radius, left arm, subsequent encounter for fracture with routine healing |
| S59229D | Salter-Harris Type II physeal fracture of lower end of radius, unspecified arm, subsequent encounter for fracture with routine healing |
| S59231D | Salter-Harris Type III physeal fracture of lower end of radius, right arm, subsequent encounter for fracture with routine healing |
| S59232D | Salter-Harris Type III physeal fracture of lower end of radius, left arm, subsequent encounter for fracture with routine healing |
| S59239D | Salter-Harris Type III physeal fracture of lower end of radius, unspecified arm, subsequent encounter for fracture with routine healing |
| S59241D | Salter-Harris Type IV physeal fracture of lower end of radius, right arm, subsequent encounter for fracture with routine healing |

| | |
|---|---|
| S59242D | Salter-Harris Type IV physeal fracture of lower end of radius, left arm, subsequent encounter for fracture with routine healing |
| S59249D | Salter-Harris Type IV physeal fracture of lower end of radius, unspecified arm, subsequent encounter for fracture with routine healing |
| S59291D | Other physeal fracture of lower end of radius, right arm, subsequent encounter for fracture with routine healing |
| S59292D | Other physeal fracture of lower end of radius, left arm, subsequent encounter for fracture with routine healing |
| S59299D | Other physeal fracture of lower end of radius, unspecified arm, subsequent encounter for fracture with routine healing |
| S62001D | Unspecified fracture of navicular [scaphoid] bone of right wrist, subsequent encounter for fracture with routine healing |
| S62002D | Unspecified fracture of navicular [scaphoid] bone of left wrist, subsequent encounter for fracture with routine healing |
| S62009D | Unspecified fracture of navicular [scaphoid] bone of unspecified wrist, subsequent encounter for fracture with routine healing |
| S62011D | Displaced fracture of distal pole of navicular [scaphoid] bone of right wrist, subsequent encounter for fracture with routine healing |
| S62012D | Displaced fracture of distal pole of navicular [scaphoid] bone of left wrist, subsequent encounter for fracture with routine healing |
| S62013D | Displaced fracture of distal pole of navicular [scaphoid] bone of unspecified wrist, subsequent encounter for fracture with routine healing |
| S62014D | Nondisplaced fracture of distal pole of navicular [scaphoid] bone of right wrist, subsequent encounter for fracture with routine healing |
| S62015D | Nondisplaced fracture of distal pole of navicular [scaphoid] bone of left wrist, subsequent encounter for fracture with routine healing |
| S62016D | Nondisplaced fracture of distal pole of navicular [scaphoid] bone of unspecified wrist, subsequent encounter for fracture with routine healing |
| S62021D | Displaced fracture of middle third of navicular [scaphoid] bone of right wrist, subsequent encounter for fracture with routine healing |
| S62022D | Displaced fracture of middle third of navicular [scaphoid] bone of left wrist, subsequent encounter for fracture with routine healing |

| | |
|---|---|
| S62023D | Displaced fracture of middle third of navicular [scaphoid] bone of unspecified wrist, subsequent encounter for fracture with routine healing |
| S62024D | Nondisplaced fracture of middle third of navicular [scaphoid] bone of right wrist, subsequent encounter for fracture with routine healing |
| S62025D | Nondisplaced fracture of middle third of navicular [scaphoid] bone of left wrist, subsequent encounter for fracture with routine healing |
| S62026D | Nondisplaced fracture of middle third of navicular [scaphoid] bone of unspecified wrist, subsequent encounter for fracture with routine healing |
| S62031D | Displaced fracture of proximal third of navicular [scaphoid] bone of right wrist, subsequent encounter for fracture with routine healing |
| S62032D | Displaced fracture of proximal third of navicular [scaphoid] bone of left wrist, subsequent encounter for fracture with routine healing |
| S62033D | Displaced fracture of proximal third of navicular [scaphoid] bone of unspecified wrist, subsequent encounter for fracture with routine healing |
| S62034D | Nondisplaced fracture of proximal third of navicular [scaphoid] bone of right wrist, subsequent encounter for fracture with routine healing |
| S62035D | Nondisplaced fracture of proximal third of navicular [scaphoid] bone of left wrist, subsequent encounter for fracture with routine healing |
| S62036D | Nondisplaced fracture of proximal third of navicular [scaphoid] bone of unspecified wrist, subsequent encounter for fracture with routine healing |
| S62101D | Fracture of unspecified carpal bone, right wrist, subsequent encounter for fracture with routine healing |
| S62102D | Fracture of unspecified carpal bone, left wrist, subsequent encounter for fracture with routine healing |
| S62109D | Fracture of unspecified carpal bone, unspecified wrist, subsequent encounter for fracture with routine healing |
| S62111D | Displaced fracture of triquetrum [cuneiform] bone, right wrist, subsequent encounter for fracture with routine healing |
| S62112D | Displaced fracture of triquetrum [cuneiform] bone, left wrist, subsequent encounter for fracture with routine healing |
| S62113D | Displaced fracture of triquetrum [cuneiform] bone, unspecified wrist, subsequent encounter for fracture with routine healing |

| | |
|---|---|
| S62114D | Nondisplaced fracture of triquetrum [cuneiform] bone, right wrist, subsequent encounter for fracture with routine healing |
| S62115D | Nondisplaced fracture of triquetrum [cuneiform] bone, left wrist, subsequent encounter for fracture with routine healing |
| S62116D | Nondisplaced fracture of triquetrum [cuneiform] bone, unspecified wrist, subsequent encounter for fracture with routine healing |
| S62121D | Displaced fracture of lunate [semilunar], right wrist, subsequent encounter for fracture with routine healing |
| S62122D | Displaced fracture of lunate [semilunar], left wrist, subsequent encounter for fracture with routine healing |
| S62123D | Displaced fracture of lunate [semilunar], unspecified wrist, subsequent encounter for fracture with routine healing |
| S62124D | Nondisplaced fracture of lunate [semilunar], right wrist, subsequent encounter for fracture with routine healing |
| S62125D | Nondisplaced fracture of lunate [semilunar], left wrist, subsequent encounter for fracture with routine healing |
| S62126D | Nondisplaced fracture of lunate [semilunar], unspecified wrist, subsequent encounter for fracture with routine healing |
| S62131D | Displaced fracture of capitate [os magnum] bone, right wrist, subsequent encounter for fracture with routine healing |
| S62132D | Displaced fracture of capitate [os magnum] bone, left wrist, subsequent encounter for fracture with routine healing |
| S62133D | Displaced fracture of capitate [os magnum] bone, unspecified wrist, subsequent encounter for fracture with routine healing |
| S62134D | Nondisplaced fracture of capitate [os magnum] bone, right wrist, subsequent encounter for fracture with routine healing |
| S62135D | Nondisplaced fracture of capitate [os magnum] bone, left wrist, subsequent encounter for fracture with routine healing |
| S62136D | Nondisplaced fracture of capitate [os magnum] bone, unspecified wrist, subsequent encounter for fracture with routine healing |
| S62141D | Displaced fracture of body of hamate [unciform] bone, right wrist, subsequent encounter for fracture with routine healing |

| | |
|---|---|
| S62142D | Displaced fracture of body of hamate [unciform] bone, left wrist, subsequent encounter for fracture with routine healing |
| S62143D | Displaced fracture of body of hamate [unciform] bone, unspecified wrist, subsequent encounter for fracture with routine healing |
| S62144D | Nondisplaced fracture of body of hamate [unciform] bone, right wrist, subsequent encounter for fracture with routine healing |
| S62145D | Nondisplaced fracture of body of hamate [unciform] bone, left wrist, subsequent encounter for fracture with routine healing |
| S62146D | Nondisplaced fracture of body of hamate [unciform] bone, unspecified wrist, subsequent encounter for fracture with routine healing |
| S62151D | Displaced fracture of hook process of hamate [unciform] bone, right wrist, subsequent encounter for fracture with routine healing |
| S62152D | Displaced fracture of hook process of hamate [unciform] bone, left wrist, subsequent encounter for fracture with routine healing |
| S62153D | Displaced fracture of hook process of hamate [unciform] bone, unspecified wrist, subsequent encounter for fracture with routine healing |
| S62154D | Nondisplaced fracture of hook process of hamate [unciform] bone, right wrist, subsequent encounter for fracture with routine healing |
| S62155D | Nondisplaced fracture of hook process of hamate [unciform] bone, left wrist, subsequent encounter for fracture with routine healing |
| S62156D | Nondisplaced fracture of hook process of hamate [unciform] bone, unspecified wrist, subsequent encounter for fracture with routine healing |
| S62161D | Displaced fracture of pisiform, right wrist, subsequent encounter for fracture with routine healing |
| S62162D | Displaced fracture of pisiform, left wrist, subsequent encounter for fracture with routine healing |
| S62163D | Displaced fracture of pisiform, unspecified wrist, subsequent encounter for fracture with routine healing |
| S62164D | Nondisplaced fracture of pisiform, right wrist, subsequent encounter for fracture with routine healing |
| S62165D | Nondisplaced fracture of pisiform, left wrist, subsequent encounter for fracture with routine healing |

| | |
|---|---|
| S62166D | Nondisplaced fracture of pisiform, unspecified wrist, subsequent encounter for fracture with routine healing |
| S62171D | Displaced fracture of trapezium [larger multangular], right wrist, subsequent encounter for fracture with routine healing |
| S62172D | Displaced fracture of trapezium [larger multangular], left wrist, subsequent encounter for fracture with routine healing |
| S62173D | Displaced fracture of trapezium [larger multangular], unspecified wrist, subsequent encounter for fracture with routine healing |
| S62174D | Nondisplaced fracture of trapezium [larger multangular], right wrist, subsequent encounter for fracture with routine healing |
| S62175D | Nondisplaced fracture of trapezium [larger multangular], left wrist, subsequent encounter for fracture with routine healing |
| S62176D | Nondisplaced fracture of trapezium [larger multangular], unspecified wrist, subsequent encounter for fracture with routine healing |
| S62181D | Displaced fracture of trapezoid [smaller multangular], right wrist, subsequent encounter for fracture with routine healing |
| S62182D | Displaced fracture of trapezoid [smaller multangular], left wrist, subsequent encounter for fracture with routine healing |
| S62183D | Displaced fracture of trapezoid [smaller multangular], unspecified wrist, subsequent encounter for fracture with routine healing |
| S62184D | Nondisplaced fracture of trapezoid [smaller multangular], right wrist, subsequent encounter for fracture with routine healing |
| S62185D | Nondisplaced fracture of trapezoid [smaller multangular], left wrist, subsequent encounter for fracture with routine healing |
| S62186D | Nondisplaced fracture of trapezoid [smaller multangular], unspecified wrist, subsequent encounter for fracture with routine healing |
| S62201D | Unspecified fracture of first metacarpal bone, right hand, subsequent encounter for fracture with routine healing |
| S62202D | Unspecified fracture of first metacarpal bone, left hand, subsequent encounter for fracture with routine healing |
| S62209D | Unspecified fracture of first metacarpal bone, unspecified hand, subsequent encounter for fracture with routine healing |

| | |
|---|---|
| S62211D | Bennetts fracture, right hand, subsequent encounter for fracture with routine healing |
| S62212D | Bennetts fracture, left hand, subsequent encounter for fracture with routine healing |
| S62213D | Bennetts fracture, unspecified hand, subsequent encounter for fracture with routine healing |
| S62221D | Displaced Rolandos fracture, right hand, subsequent encounter for fracture with routine healing |
| S62222D | Displaced Rolandos fracture, left hand, subsequent encounter for fracture with routine healing |
| S62223D | Displaced Rolandos fracture, unspecified hand, subsequent encounter for fracture with routine healing |
| S62224D | Nondisplaced Rolandos fracture, right hand, subsequent encounter for fracture with routine healing |
| S62225D | Nondisplaced Rolandos fracture, left hand, subsequent encounter for fracture with routine healing |
| S62226D | Nondisplaced Rolandos fracture, unspecified hand, subsequent encounter for fracture with routine healing |
| S62231D | Other displaced fracture of base of first metacarpal bone, right hand, subsequent encounter for fracture with routine healing |
| S62232D | Other displaced fracture of base of first metacarpal bone, left hand, subsequent encounter for fracture with routine healing |
| S62233D | Other displaced fracture of base of first metacarpal bone, unspecified hand, subsequent encounter for fracture with routine healing |
| S62234D | Other nondisplaced fracture of base of first metacarpal bone, right hand, subsequent encounter for fracture with routine healing |
| S62235D | Other nondisplaced fracture of base of first metacarpal bone, left hand, subsequent encounter for fracture with routine healing |
| S62236D | Other nondisplaced fracture of base of first metacarpal bone, unspecified hand, subsequent encounter for fracture with routine healing |
| S62241D | Displaced fracture of shaft of first metacarpal bone, right hand, subsequent encounter for fracture with routine healing |

| | |
|---|---|
| S62242D | Displaced fracture of shaft of first metacarpal bone, left hand, subsequent encounter for fracture with routine healing |
| S62243D | Displaced fracture of shaft of first metacarpal bone, unspecified hand, subsequent encounter for fracture with routine healing |
| S62244D | Nondisplaced fracture of shaft of first metacarpal bone, right hand, subsequent encounter for fracture with routine healing |
| S62245D | Nondisplaced fracture of shaft of first metacarpal bone, left hand, subsequent encounter for fracture with routine healing |
| S62246D | Nondisplaced fracture of shaft of first metacarpal bone, unspecified hand, subsequent encounter for fracture with routine healing |
| S62251D | Displaced fracture of neck of first metacarpal bone, right hand, subsequent encounter for fracture with routine healing |
| S62252D | Displaced fracture of neck of first metacarpal bone, left hand, subsequent encounter for fracture with routine healing |
| S62253D | Displaced fracture of neck of first metacarpal bone, unspecified hand, subsequent encounter for fracture with routine healing |
| S62254D | Nondisplaced fracture of neck of first metacarpal bone, right hand, subsequent encounter for fracture with routine healing |
| S62255D | Nondisplaced fracture of neck of first metacarpal bone, left hand, subsequent encounter for fracture with routine healing |
| S62256D | Nondisplaced fracture of neck of first metacarpal bone, unspecified hand, subsequent encounter for fracture with routine healing |
| S62291D | Other fracture of first metacarpal bone, right hand, subsequent encounter for fracture with routine healing |
| S62292D | Other fracture of first metacarpal bone, left hand, subsequent encounter for fracture with routine healing |
| S62299D | Other fracture of first metacarpal bone, unspecified hand, subsequent encounter for fracture with routine healing |
| S62300D | Unspecified fracture of second metacarpal bone, right hand, subsequent encounter for fracture with routine healing |
| S62301D | Unspecified fracture of second metacarpal bone, left hand, subsequent encounter for fracture with routine healing |

| | |
|---|---|
| S62302D | Unspecified fracture of third metacarpal bone, right hand, subsequent encounter for fracture with routine healing |
| S62303D | Unspecified fracture of third metacarpal bone, left hand, subsequent encounter for fracture with routine healing |
| S62304D | Unspecified fracture of fourth metacarpal bone, right hand, subsequent encounter for fracture with routine healing |
| S62305D | Unspecified fracture of fourth metacarpal bone, left hand, subsequent encounter for fracture with routine healing |
| S62306D | Unspecified fracture of fifth metacarpal bone, right hand, subsequent encounter for fracture with routine healing |
| S62307D | Unspecified fracture of fifth metacarpal bone, left hand, subsequent encounter for fracture with routine healing |
| S62308D | Unspecified fracture of other metacarpal bone, subsequent encounter for fracture with routine healing |
| S62309D | Unspecified fracture of unspecified metacarpal bone, subsequent encounter for fracture with routine healing |
| S62310D | Displaced fracture of base of second metacarpal bone, right hand, subsequent encounter for fracture with routine healing |
| S62311D | Displaced fracture of base of second metacarpal bone, left hand, subsequent encounter for fracture with routine healing |
| S62312D | Displaced fracture of base of third metacarpal bone, right hand, subsequent encounter for fracture with routine healing |
| S62313D | Displaced fracture of base of third metacarpal bone, left hand, subsequent encounter for fracture with routine healing |
| S62314D | Displaced fracture of base of fourth metacarpal bone, right hand, subsequent encounter for fracture with routine healing |
| S62315D | Displaced fracture of base of fourth metacarpal bone, left hand, subsequent encounter for fracture with routine healing |
| S62316D | Displaced fracture of base of fifth metacarpal bone, right hand, subsequent encounter for fracture with routine healing |
| S62317D | Displaced fracture of base of fifth metacarpal bone, left hand, subsequent encounter for fracture with routine healing |

| | |
|---|---|
| S62318D | Displaced fracture of base of other metacarpal bone, subsequent encounter for fracture with routine healing |
| S62319D | Displaced fracture of base of unspecified metacarpal bone, subsequent encounter for fracture with routine healing |
| S62320D | Displaced fracture of shaft of second metacarpal bone, right hand, subsequent encounter for fracture with routine healing |
| S62321D | Displaced fracture of shaft of second metacarpal bone, left hand, subsequent encounter for fracture with routine healing |
| S62322D | Displaced fracture of shaft of third metacarpal bone, right hand, subsequent encounter for fracture with routine healing |
| S62323D | Displaced fracture of shaft of third metacarpal bone, left hand, subsequent encounter for fracture with routine healing |
| S62324D | Displaced fracture of shaft of fourth metacarpal bone, right hand, subsequent encounter for fracture with routine healing |
| S62325D | Displaced fracture of shaft of fourth metacarpal bone, left hand, subsequent encounter for fracture with routine healing |
| S62326D | Displaced fracture of shaft of fifth metacarpal bone, right hand, subsequent encounter for fracture with routine healing |
| S62327D | Displaced fracture of shaft of fifth metacarpal bone, left hand, subsequent encounter for fracture with routine healing |
| S62328D | Displaced fracture of shaft of other metacarpal bone, subsequent encounter for fracture with routine healing |
| S62329D | Displaced fracture of shaft of unspecified metacarpal bone, subsequent encounter for fracture with routine healing |
| S62330D | Displaced fracture of neck of second metacarpal bone, right hand, subsequent encounter for fracture with routine healing |
| S62331D | Displaced fracture of neck of second metacarpal bone, left hand, subsequent encounter for fracture with routine healing |
| S62332D | Displaced fracture of neck of third metacarpal bone, right hand, subsequent encounter for fracture with routine healing |
| S62333D | Displaced fracture of neck of third metacarpal bone, left hand, subsequent encounter for fracture with routine healing |

| | |
|---|---|
| S62334D | Displaced fracture of neck of fourth metacarpal bone, right hand, subsequent encounter for fracture with routine healing |
| S62335D | Displaced fracture of neck of fourth metacarpal bone, left hand, subsequent encounter for fracture with routine healing |
| S62336D | Displaced fracture of neck of fifth metacarpal bone, right hand, subsequent encounter for fracture with routine healing |
| S62337D | Displaced fracture of neck of fifth metacarpal bone, left hand, subsequent encounter for fracture with routine healing |
| S62338D | Displaced fracture of neck of other metacarpal bone, subsequent encounter for fracture with routine healing |
| S62339D | Displaced fracture of neck of unspecified metacarpal bone, subsequent encounter for fracture with routine healing |
| S62340D | Nondisplaced fracture of base of second metacarpal bone, right hand, subsequent encounter for fracture with routine healing |
| S62341D | Nondisplaced fracture of base of second metacarpal bone, left hand, subsequent encounter for fracture with routine healing |
| S62342D | Nondisplaced fracture of base of third metacarpal bone, right hand, subsequent encounter for fracture with routine healing |
| S62343D | Nondisplaced fracture of base of third metacarpal bone, left hand, subsequent encounter for fracture with routine healing |
| S62344D | Nondisplaced fracture of base of fourth metacarpal bone, right hand, subsequent encounter for fracture with routine healing |
| S62345D | Nondisplaced fracture of base of fourth metacarpal bone, left hand, subsequent encounter for fracture with routine healing |
| S62346D | Nondisplaced fracture of base of fifth metacarpal bone, right hand, subsequent encounter for fracture with routine healing |
| S62347D | Nondisplaced fracture of base of fifth metacarpal bone, left hand, subsequent encounter for fracture with routine healing |
| S62348D | Nondisplaced fracture of base of other metacarpal bone, subsequent encounter for fracture with routine healing |
| S62349D | Nondisplaced fracture of base of unspecified metacarpal bone, subsequent encounter for fracture with routine healing |

| | |
|---|---|
| S62350D | Nondisplaced fracture of shaft of second metacarpal bone, right hand, subsequent encounter for fracture with routine healing |
| S62351D | Nondisplaced fracture of shaft of second metacarpal bone, left hand, subsequent encounter for fracture with routine healing |
| S62352D | Nondisplaced fracture of shaft of third metacarpal bone, right hand, subsequent encounter for fracture with routine healing |
| S62353D | Nondisplaced fracture of shaft of third metacarpal bone, left hand, subsequent encounter for fracture with routine healing |
| S62354D | Nondisplaced fracture of shaft of fourth metacarpal bone, right hand, subsequent encounter for fracture with routine healing |
| S62355D | Nondisplaced fracture of shaft of fourth metacarpal bone, left hand, subsequent encounter for fracture with routine healing |
| S62356D | Nondisplaced fracture of shaft of fifth metacarpal bone, right hand, subsequent encounter for fracture with routine healing |
| S62357D | Nondisplaced fracture of shaft of fifth metacarpal bone, left hand, subsequent encounter for fracture with routine healing |
| S62358D | Nondisplaced fracture of shaft of other metacarpal bone, subsequent encounter for fracture with routine healing |
| S62359D | Nondisplaced fracture of shaft of unspecified metacarpal bone, subsequent encounter for fracture with routine healing |
| S62360D | Nondisplaced fracture of neck of second metacarpal bone, right hand, subsequent encounter for fracture with routine healing |
| S62361D | Nondisplaced fracture of neck of second metacarpal bone, left hand, subsequent encounter for fracture with routine healing |
| S62362D | Nondisplaced fracture of neck of third metacarpal bone, right hand, subsequent encounter for fracture with routine healing |
| S62363D | Nondisplaced fracture of neck of third metacarpal bone, left hand, subsequent encounter for fracture with routine healing |
| S62364D | Nondisplaced fracture of neck of fourth metacarpal bone, right hand, subsequent encounter for fracture with routine healing |
| S62365D | Nondisplaced fracture of neck of fourth metacarpal bone, left hand, subsequent encounter for fracture with routine healing |

| | |
|---|---|
| S62366D | Nondisplaced fracture of neck of fifth metacarpal bone, right hand, subsequent encounter for fracture with routine healing |
| S62367D | Nondisplaced fracture of neck of fifth metacarpal bone, left hand, subsequent encounter for fracture with routine healing |
| S62368D | Nondisplaced fracture of neck of other metacarpal bone, subsequent encounter for fracture with routine healing |
| S62369D | Nondisplaced fracture of neck of unspecified metacarpal bone, subsequent encounter for fracture with routine healing |
| S62390D | Other fracture of second metacarpal bone, right hand, subsequent encounter for fracture with routine healing |
| S62391D | Other fracture of second metacarpal bone, left hand, subsequent encounter for fracture with routine healing |
| S62392D | Other fracture of third metacarpal bone, right hand, subsequent encounter for fracture with routine healing |
| S62393D | Other fracture of third metacarpal bone, left hand, subsequent encounter for fracture with routine healing |
| S62394D | Other fracture of fourth metacarpal bone, right hand, subsequent encounter for fracture with routine healing |
| S62395D | Other fracture of fourth metacarpal bone, left hand, subsequent encounter for fracture with routine healing |
| S62396D | Other fracture of fifth metacarpal bone, right hand, subsequent encounter for fracture with routine healing |
| S62397D | Other fracture of fifth metacarpal bone, left hand, subsequent encounter for fracture with routine healing |
| S62398D | Other fracture of other metacarpal bone, subsequent encounter for fracture with routine healing |
| S62399D | Other fracture of unspecified metacarpal bone, subsequent encounter for fracture with routine healing |

| | |
|---|---|
| S62501D | Fracture of unspecified phalanx of right thumb, subsequent encounter for fracture with routine healing |
| S62502D | Fracture of unspecified phalanx of left thumb, subsequent encounter for fracture with routine healing |

| | |
|---|---|
| S62509D | Fracture of unspecified phalanx of unspecified thumb, subsequent encounter for fracture with routine healing |
| S62511D | Displaced fracture of proximal phalanx of right thumb, subsequent encounter for fracture with routine healing |
| S62512D | Displaced fracture of proximal phalanx of left thumb, subsequent encounter for fracture with routine healing |
| S62513D | Displaced fracture of proximal phalanx of unspecified thumb, subsequent encounter for fracture with routine healing |
| S62514D | Nondisplaced fracture of proximal phalanx of right thumb, subsequent encounter for fracture with routine healing |
| S62515D | Nondisplaced fracture of proximal phalanx of left thumb, subsequent encounter for fracture with routine healing |
| S62516D | Nondisplaced fracture of proximal phalanx of unspecified thumb, subsequent encounter for fracture with routine healing |
| S62521D | Displaced fracture of distal phalanx of right thumb, subsequent encounter for fracture with routine healing |
| S62522D | Displaced fracture of distal phalanx of left thumb, subsequent encounter for fracture with routine healing |
| S62523D | Displaced fracture of distal phalanx of unspecified thumb, subsequent encounter for fracture with routine healing |
| S62524D | Nondisplaced fracture of distal phalanx of right thumb, subsequent encounter for fracture with routine healing |
| S62525D | Nondisplaced fracture of distal phalanx of left thumb, subsequent encounter for fracture with routine healing |
| S62526D | Nondisplaced fracture of distal phalanx of unspecified thumb, subsequent encounter for fracture with routine healing |
| S62600D | Fracture of unspecified phalanx of right index finger, subsequent encounter for fracture with routine healing |
| S62601D | Fracture of unspecified phalanx of left index finger, subsequent encounter for fracture with routine healing |
| S62602D | Fracture of unspecified phalanx of right middle finger, subsequent encounter for fracture with routine healing |

| | |
|---|---|
| S62603D | Fracture of unspecified phalanx of left middle finger, subsequent encounter for fracture with routine healing |
| S62604D | Fracture of unspecified phalanx of right ring finger, subsequent encounter for fracture with routine healing |
| S62605D | Fracture of unspecified phalanx of left ring finger, subsequent encounter for fracture with routine healing |
| S62606D | Fracture of unspecified phalanx of right little finger, subsequent encounter for fracture with routine healing |
| S62607D | Fracture of unspecified phalanx of left little finger, subsequent encounter for fracture with routine healing |
| S62608D | Fracture of unspecified phalanx of other finger, subsequent encounter for fracture with routine healing |
| S62609D | Fracture of unspecified phalanx of unspecified finger, subsequent encounter for fracture with routine healing |
| S62610D | Displaced fracture of proximal phalanx of right index finger, subsequent encounter for fracture with routine healing |
| S62611D | Displaced fracture of proximal phalanx of left index finger, subsequent encounter for fracture with routine healing |
| S62612D | Displaced fracture of proximal phalanx of right middle finger, subsequent encounter for fracture with routine healing |
| S62613D | Displaced fracture of proximal phalanx of left middle finger, subsequent encounter for fracture with routine healing |
| S62614D | Displaced fracture of proximal phalanx of right ring finger, subsequent encounter for fracture with routine healing |
| S62615D | Displaced fracture of proximal phalanx of left ring finger, subsequent encounter for fracture with routine healing |
| S62616D | Displaced fracture of proximal phalanx of right little finger, subsequent encounter for fracture with routine healing |
| S62617D | Displaced fracture of proximal phalanx of left little finger, subsequent encounter for fracture with routine healing |
| S62618D | Displaced fracture of proximal phalanx of other finger, subsequent encounter for fracture with routine healing |

| | |
|---|---|
| S62619D | Displaced fracture of proximal phalanx of unspecified finger, subsequent encounter for fracture with routine healing |
| S62620D | Displaced fracture of middle phalanx of right index finger, subsequent encounter for fracture with routine healing |
| S62621D | Displaced fracture of middle phalanx of left index finger, subsequent encounter for fracture with routine healing |
| S62622D | Displaced fracture of middle phalanx of right middle finger, subsequent encounter for fracture with routine healing |
| S62623D | Displaced fracture of middle phalanx of left middle finger, subsequent encounter for fracture with routine healing |
| S62624D | Displaced fracture of middle phalanx of right ring finger, subsequent encounter for fracture with routine healing |
| S62625D | Displaced fracture of middle phalanx of left ring finger, subsequent encounter for fracture with routine healing |
| S62626D | Displaced fracture of middle phalanx of right little finger, subsequent encounter for fracture with routine healing |
| S62627D | Displaced fracture of middle phalanx of left little finger, subsequent encounter for fracture with routine healing |
| S62628D | Displaced fracture of middle phalanx of other finger, subsequent encounter for fracture with routine healing |
| S62629D | Displaced fracture of middle phalanx of unspecified finger, subsequent encounter for fracture with routine healing |
| S62630D | Displaced fracture of distal phalanx of right index finger, subsequent encounter for fracture with routine healing |
| S62631D | Displaced fracture of distal phalanx of left index finger, subsequent encounter for fracture with routine healing |
| S62632D | Displaced fracture of distal phalanx of right middle finger, subsequent encounter for fracture with routine healing |
| S62633D | Displaced fracture of distal phalanx of left middle finger, subsequent encounter for fracture with routine healing |
| S62634D | Displaced fracture of distal phalanx of right ring finger, subsequent encounter for fracture with routine healing |

| | |
|---|---|
| S62635D | Displaced fracture of distal phalanx of left ring finger, subsequent encounter for fracture with routine healing |
| S62636D | Displaced fracture of distal phalanx of right little finger, subsequent encounter for fracture with routine healing |
| S62637D | Displaced fracture of distal phalanx of left little finger, subsequent encounter for fracture with routine healing |
| S62638D | Displaced fracture of distal phalanx of other finger, subsequent encounter for fracture with routine healing |
| S62639D | Displaced fracture of distal phalanx of unspecified finger, subsequent encounter for fracture with routine healing |
| S62640D | Nondisplaced fracture of proximal phalanx of right index finger, subsequent encounter for fracture with routine healing |
| S62641D | Nondisplaced fracture of proximal phalanx of left index finger, subsequent encounter for fracture with routine healing |
| S62642D | Nondisplaced fracture of proximal phalanx of right middle finger, subsequent encounter for fracture with routine healing |
| S62643D | Nondisplaced fracture of proximal phalanx of left middle finger, subsequent encounter for fracture with routine healing |
| S62644D | Nondisplaced fracture of proximal phalanx of right ring finger, subsequent encounter for fracture with routine healing |
| S62645D | Nondisplaced fracture of proximal phalanx of left ring finger, subsequent encounter for fracture with routine healing |
| S62646D | Nondisplaced fracture of proximal phalanx of right little finger, subsequent encounter for fracture with routine healing |
| S62647D | Nondisplaced fracture of proximal phalanx of left little finger, subsequent encounter for fracture with routine healing |
| S62648D | Nondisplaced fracture of proximal phalanx of other finger, subsequent encounter for fracture with routine healing |
| S62649D | Nondisplaced fracture of proximal phalanx of unspecified finger, subsequent encounter for fracture with routine healing |
| S62650D | Nondisplaced fracture of middle phalanx of right index finger, subsequent encounter for fracture with routine healing |

| | |
|---|---|
| S62651D | Nondisplaced fracture of middle phalanx of left index finger, subsequent encounter for fracture with routine healing |
| S62652D | Nondisplaced fracture of middle phalanx of right middle finger, subsequent encounter for fracture with routine healing |
| S62653D | Nondisplaced fracture of middle phalanx of left middle finger, subsequent encounter for fracture with routine healing |
| S62654D | Nondisplaced fracture of middle phalanx of right ring finger, subsequent encounter for fracture with routine healing |
| S62655D | Nondisplaced fracture of middle phalanx of left ring finger, subsequent encounter for fracture with routine healing |
| S62656D | Nondisplaced fracture of middle phalanx of right little finger, subsequent encounter for fracture with routine healing |
| S62657D | Nondisplaced fracture of middle phalanx of left little finger, subsequent encounter for fracture with routine healing |
| S62658D | Nondisplaced fracture of middle phalanx of other finger, subsequent encounter for fracture with routine healing |
| S62659D | Nondisplaced fracture of middle phalanx of unspecified finger, subsequent encounter for fracture with routine healing |
| S62660D | Nondisplaced fracture of distal phalanx of right index finger, subsequent encounter for fracture with routine healing |
| S62661D | Nondisplaced fracture of distal phalanx of left index finger, subsequent encounter for fracture with routine healing |
| S62662D | Nondisplaced fracture of distal phalanx of right middle finger, subsequent encounter for fracture with routine healing |
| S62663D | Nondisplaced fracture of distal phalanx of left middle finger, subsequent encounter for fracture with routine healing |
| S62664D | Nondisplaced fracture of distal phalanx of right ring finger, subsequent encounter for fracture with routine healing |
| S62665D | Nondisplaced fracture of distal phalanx of left ring finger, subsequent encounter for fracture with routine healing |
| S62666D | Nondisplaced fracture of distal phalanx of right little finger, subsequent encounter for fracture with routine healing |

| | |
|---|---|
| S62667D | Nondisplaced fracture of distal phalanx of left little finger, subsequent encounter for fracture with routine healing |
| S62668D | Nondisplaced fracture of distal phalanx of other finger, subsequent encounter for fracture with routine healing |
| S62669D | Nondisplaced fracture of distal phalanx of unspecified finger, subsequent encounter for fracture with routine healing |
| S6290XD | Unspecified fracture of unspecified wrist and hand, subsequent encounter for fracture with routine healing |
| S6291XD | Unspecified fracture of right wrist and hand, subsequent encounter for fracture with routine healing |
| S6292XD | Unspecified fracture of left wrist and hand, subsequent encounter for fracture with routine healing |
