# Supplementary material for: Exploring the relationship between frailty and nonunion fractures in upper extremity injuries: insights from the national inpatient sample
Source: Eur J Orthop Surg Traumatol. 2025 Mar 14;35(1):115. doi: 10.1007/s00590-025-04247-y (PMC11909083; doi:10.1007/s00590-025-04247-y)
Supplement: Supplementary file 2 — Supplementary file2 (PDF 311 kb) [file 590_2025_4247_MOESM2_ESM.pdf]

Appendix 2

Nonunion ICD-10 Codes

Clavicle	
S42001K	Fracture of unspecified part of right clavicle, subsequent encounter for fracture with nonunion
S42002K	Fracture of unspecified part of left clavicle, subsequent encounter for fracture with nonunion
S42009K	Fracture of unspecified part of unspecified clavicle, subsequent encounter for fracture with nonunion
S42011K	Anterior displaced fracture of sternal end of right clavicle, subsequent encounter for fracture with nonunion
S42012K	Anterior displaced fracture of sternal end of left clavicle, subsequent encounter for fracture with nonunion
S42013K	Anterior displaced fracture of sternal end of unspecified clavicle, subsequent encounter for fracture with nonunion
S42014K	Posterior displaced fracture of sternal end of right clavicle, subsequent encounter for fracture with nonunion
S42015K	Posterior displaced fracture of sternal end of left clavicle, subsequent encounter for fracture with nonunion
S42016K	Posterior displaced fracture of sternal end of unspecified clavicle, subsequent encounter for fracture with nonunion
S42017K	Nondisplaced fracture of sternal end of right clavicle, subsequent encounter for fracture with nonunion
S42018K	Nondisplaced fracture of sternal end of left clavicle, subsequent encounter for fracture with nonunion
S42019K	Nondisplaced fracture of sternal end of unspecified clavicle, subsequent encounter for fracture with nonunion
S42021K	Displaced fracture of shaft of right clavicle, subsequent encounter for fracture with nonunion
S42022K	Displaced fracture of shaft of left clavicle, subsequent encounter for fracture with nonunion
S42023K	Displaced fracture of shaft of unspecified clavicle, subsequent encounter for fracture with nonunion

S42024K	Nondisplaced fracture of shaft of right clavicle, subsequent encounter for fracture with nonunion
S42025K	Nondisplaced fracture of shaft of left clavicle, subsequent encounter for fracture with nonunion
S42026K	Nondisplaced fracture of shaft of unspecified clavicle, subsequent encounter for fracture with nonunion
S42031K	Displaced fracture of lateral end of right clavicle, subsequent encounter for fracture with nonunion
S42032K	Displaced fracture of lateral end of left clavicle, subsequent encounter for fracture with nonunion
S42033K	Displaced fracture of lateral end of unspecified clavicle, subsequent encounter for fracture with nonunion
S42034K	Nondisplaced fracture of lateral end of right clavicle, subsequent encounter for fracture with nonunion
S42035K	Nondisplaced fracture of lateral end of left clavicle, subsequent encounter for fracture with nonunion
S42036K	Nondisplaced fracture of lateral end of unspecified clavicle, subsequent encounter for fracture with nonunion
**Scapula**	
S42101K	Fracture of unspecified part of scapula, right shoulder, subsequent encounter for fracture with nonunion
S42102K	Fracture of unspecified part of scapula, left shoulder, subsequent encounter for fracture with nonunion
S42109K	Fracture of unspecified part of scapula, unspecified shoulder, subsequent encounter for fracture with nonunion
S42111K	Displaced fracture of body of scapula, right shoulder, subsequent encounter for fracture with nonunion
S42112K	Displaced fracture of body of scapula, left shoulder, subsequent encounter for fracture with nonunion
S42113K	Displaced fracture of body of scapula, unspecified shoulder, subsequent encounter for fracture with nonunion
S42114K	Nondisplaced fracture of body of scapula, right shoulder, subsequent encounter for fracture with nonunion

S42115K	Nondisplaced fracture of body of scapula, left shoulder, subsequent encounter for fracture with nonunion
S42116K	Nondisplaced fracture of body of scapula, unspecified shoulder, subsequent encounter for fracture with nonunion
S42121K	Displaced fracture of acromial process, right shoulder, subsequent encounter for fracture with nonunion
S42122K	Displaced fracture of acromial process, left shoulder, subsequent encounter for fracture with nonunion
S42123K	Displaced fracture of acromial process, unspecified shoulder, subsequent encounter for fracture with nonunion
S42124K	Nondisplaced fracture of acromial process, right shoulder, subsequent encounter for fracture with nonunion
S42125K	Nondisplaced fracture of acromial process, left shoulder, subsequent encounter for fracture with nonunion
S42126K	Nondisplaced fracture of acromial process, unspecified shoulder, subsequent encounter for fracture with nonunion
S42131K	Displaced fracture of coracoid process, right shoulder, subsequent encounter for fracture with nonunion
S42132K	Displaced fracture of coracoid process, left shoulder, subsequent encounter for fracture with nonunion
S42133K	Displaced fracture of coracoid process, unspecified shoulder, subsequent encounter for fracture with nonunion
S42134K	Nondisplaced fracture of coracoid process, right shoulder, subsequent encounter for fracture with nonunion
S42135K	Nondisplaced fracture of coracoid process, left shoulder, subsequent encounter for fracture with nonunion
S42136K	Nondisplaced fracture of coracoid process, unspecified shoulder, subsequent encounter for fracture with nonunion
S42141K	Displaced fracture of glenoid cavity of scapula, right shoulder, subsequent encounter for fracture with nonunion
S42142K	Displaced fracture of glenoid cavity of scapula, left shoulder, subsequent encounter for fracture with nonunion

S42143K	Displaced fracture of glenoid cavity of scapula, unspecified shoulder, subsequent encounter for fracture with nonunion
S42144K	Nondisplaced fracture of glenoid cavity of scapula, right shoulder, subsequent encounter for fracture with nonunion
S42145K	Nondisplaced fracture of glenoid cavity of scapula, left shoulder, subsequent encounter for fracture with nonunion
S42146K	Nondisplaced fracture of glenoid cavity of scapula, unspecified shoulder, subsequent encounter for fracture with nonunion
S42151K	Displaced fracture of neck of scapula, right shoulder, subsequent encounter for fracture with nonunion
S42152K	Displaced fracture of neck of scapula, left shoulder, subsequent encounter for fracture with nonunion
S42153K	Displaced fracture of neck of scapula, unspecified shoulder, subsequent encounter for fracture with nonunion
S42154K	Nondisplaced fracture of neck of scapula, right shoulder, subsequent encounter for fracture with nonunion
S42155K	Nondisplaced fracture of neck of scapula, left shoulder, subsequent encounter for fracture with nonunion
S42156K	Nondisplaced fracture of neck of scapula, unspecified shoulder, subsequent encounter for fracture with nonunion
S42191K	Fracture of other part of scapula, right shoulder, subsequent encounter for fracture with nonunion
S42192K	Fracture of other part of scapula, left shoulder, subsequent encounter for fracture with nonunion
S42199K	Fracture of other part of scapula, unspecified shoulder, subsequent encounter for fracture with nonunion
**Humerus**	
S42201K	Unspecified fracture of upper end of right humerus, subsequent encounter for fracture with nonunion
S42202K	Unspecified fracture of upper end of left humerus, subsequent encounter for fracture with nonunion
S42209K	Unspecified fracture of upper end of unspecified humerus, subsequent encounter for fracture with nonunion

S42211K	Unspecified displaced fracture of surgical neck of right humerus, subsequent encounter for fracture with nonunion
S42212K	Unspecified displaced fracture of surgical neck of left humerus, subsequent encounter for fracture with nonunion
S42213K	Unspecified displaced fracture of surgical neck of unspecified humerus, subsequent encounter for fracture with nonunion
S42214K	Unspecified nondisplaced fracture of surgical neck of right humerus, subsequent encounter for fracture with nonunion
S42215K	Unspecified nondisplaced fracture of surgical neck of left humerus, subsequent encounter for fracture with nonunion
S42216K	Unspecified nondisplaced fracture of surgical neck of unspecified humerus, subsequent encounter for fracture with nonunion
S42221K	2-part displaced fracture of surgical neck of right humerus, subsequent encounter for fracture with nonunion
S42222K	2-part displaced fracture of surgical neck of left humerus, subsequent encounter for fracture with nonunion
S42223K	2-part displaced fracture of surgical neck of unspecified humerus, subsequent encounter for fracture with nonunion
S42224K	2-part nondisplaced fracture of surgical neck of right humerus, subsequent encounter for fracture with nonunion
S42225K	2-part nondisplaced fracture of surgical neck of left humerus, subsequent encounter for fracture with nonunion
S42226K	2-part nondisplaced fracture of surgical neck of unspecified humerus, subsequent encounter for fracture with nonunion
S42231K	3-part fracture of surgical neck of right humerus, subsequent encounter for fracture with nonunion
S42232K	3-part fracture of surgical neck of left humerus, subsequent encounter for fracture with nonunion
S42239K	3-part fracture of surgical neck of unspecified humerus, subsequent encounter for fracture with nonunion
S42241K	4-part fracture of surgical neck of right humerus, subsequent encounter for fracture with nonunion

S42242K	4-part fracture of surgical neck of left humerus, subsequent encounter for fracture with nonunion
S42249K	4-part fracture of surgical neck of unspecified humerus, subsequent encounter for fracture with nonunion
S42251K	Displaced fracture of greater tuberosity of right humerus, subsequent encounter for fracture with nonunion
S42252K	Displaced fracture of greater tuberosity of left humerus, subsequent encounter for fracture with nonunion
S42253K	Displaced fracture of greater tuberosity of unspecified humerus, subsequent encounter for fracture with nonunion
S42254K	Nondisplaced fracture of greater tuberosity of right humerus, subsequent encounter for fracture with nonunion
S42255K	Nondisplaced fracture of greater tuberosity of left humerus, subsequent encounter for fracture with nonunion
S42256K	Nondisplaced fracture of greater tuberosity of unspecified humerus, subsequent encounter for fracture with nonunion
S42261K	Displaced fracture of lesser tuberosity of right humerus, subsequent encounter for fracture with nonunion
S42262K	Displaced fracture of lesser tuberosity of left humerus, subsequent encounter for fracture with nonunion
S42263K	Displaced fracture of lesser tuberosity of unspecified humerus, subsequent encounter for fracture with nonunion
S42264K	Nondisplaced fracture of lesser tuberosity of right humerus, subsequent encounter for fracture with nonunion
S42265K	Nondisplaced fracture of lesser tuberosity of left humerus, subsequent encounter for fracture with nonunion
S42266K	Nondisplaced fracture of lesser tuberosity of unspecified humerus, subsequent encounter for fracture with nonunion
S42271K	Torus fracture of upper end of right humerus, subsequent encounter for fracture with nonunion
S42272K	Torus fracture of upper end of left humerus, subsequent encounter for fracture with nonunion

S42279K	Torus fracture of upper end of unspecified humerus, subsequent encounter for fracture with nonunion
S42291K	Other displaced fracture of upper end of right humerus, subsequent encounter for fracture with nonunion
S42292K	Other displaced fracture of upper end of left humerus, subsequent encounter for fracture with nonunion
S42293K	Other displaced fracture of upper end of unspecified humerus, subsequent encounter for fracture with nonunion
S42294K	Other nondisplaced fracture of upper end of right humerus, subsequent encounter for fracture with nonunion
S42295K	Other nondisplaced fracture of upper end of left humerus, subsequent encounter for fracture with nonunion
S42296K	Other nondisplaced fracture of upper end of unspecified humerus, subsequent encounter for fracture with nonunion
S42301K	Unspecified fracture of shaft of humerus, right arm, subsequent encounter for fracture with nonunion
S42302K	Unspecified fracture of shaft of humerus, left arm, subsequent encounter for fracture with nonunion
S42309K	Unspecified fracture of shaft of humerus, unspecified arm, subsequent encounter for fracture with nonunion
S42311K	Greenstick fracture of shaft of humerus, right arm, subsequent encounter for fracture with nonunion
S42312K	Greenstick fracture of shaft of humerus, left arm, subsequent encounter for fracture with nonunion
S42319K	Greenstick fracture of shaft of humerus, unspecified arm, subsequent encounter for fracture with nonunion
S42321K	Displaced transverse fracture of shaft of humerus, right arm, subsequent encounter for fracture with nonunion
S42322K	Displaced transverse fracture of shaft of humerus, left arm, subsequent encounter for fracture with nonunion
S42323K	Displaced transverse fracture of shaft of humerus, unspecified arm, subsequent encounter for fracture with nonunion

S42324K	Nondisplaced transverse fracture of shaft of humerus, right arm, subsequent encounter for fracture with nonunion
S42325K	Nondisplaced transverse fracture of shaft of humerus, left arm, subsequent encounter for fracture with nonunion
S42326K	Nondisplaced transverse fracture of shaft of humerus, unspecified arm, subsequent encounter for fracture with nonunion
S42331K	Displaced oblique fracture of shaft of humerus, right arm, subsequent encounter for fracture with nonunion
S42332K	Displaced oblique fracture of shaft of humerus, left arm, subsequent encounter for fracture with nonunion
S42333K	Displaced oblique fracture of shaft of humerus, unspecified arm, subsequent encounter for fracture with nonunion
S42334K	Nondisplaced oblique fracture of shaft of humerus, right arm, subsequent encounter for fracture with nonunion
S42335K	Nondisplaced oblique fracture of shaft of humerus, left arm, subsequent encounter for fracture with nonunion
S42336K	Nondisplaced oblique fracture of shaft of humerus, unspecified arm, subsequent encounter for fracture with nonunion
S42341K	Displaced spiral fracture of shaft of humerus, right arm, subsequent encounter for fracture with nonunion
S42342K	Displaced spiral fracture of shaft of humerus, left arm, subsequent encounter for fracture with nonunion
S42343K	Displaced spiral fracture of shaft of humerus, unspecified arm, subsequent encounter for fracture with nonunion
S42344K	Nondisplaced spiral fracture of shaft of humerus, right arm, subsequent encounter for fracture with nonunion
S42345K	Nondisplaced spiral fracture of shaft of humerus, left arm, subsequent encounter for fracture with nonunion
S42346K	Nondisplaced spiral fracture of shaft of humerus, unspecified arm, subsequent encounter for fracture with nonunion
S42351K	Displaced comminuted fracture of shaft of humerus, right arm, subsequent encounter for fracture with nonunion

S42352K	Displaced comminuted fracture of shaft of humerus, left arm, subsequent encounter for fracture with nonunion
S42353K	Displaced comminuted fracture of shaft of humerus, unspecified arm, subsequent encounter for fracture with nonunion
S42354K	Nondisplaced comminuted fracture of shaft of humerus, right arm, subsequent encounter for fracture with nonunion
S42355K	Nondisplaced comminuted fracture of shaft of humerus, left arm, subsequent encounter for fracture with nonunion
S42356K	Nondisplaced comminuted fracture of shaft of humerus, unspecified arm, subsequent encounter for fracture with nonunion
S42361K	Displaced segmental fracture of shaft of humerus, right arm, subsequent encounter for fracture with nonunion
S42362K	Displaced segmental fracture of shaft of humerus, left arm, subsequent encounter for fracture with nonunion
S42363K	Displaced segmental fracture of shaft of humerus, unspecified arm, subsequent encounter for fracture with nonunion
S42364K	Nondisplaced segmental fracture of shaft of humerus, right arm, subsequent encounter for fracture with nonunion
S42365K	Nondisplaced segmental fracture of shaft of humerus, left arm, subsequent encounter for fracture with nonunion
S42366K	Nondisplaced segmental fracture of shaft of humerus, unspecified arm, subsequent encounter for fracture with nonunion
S42391K	Other fracture of shaft of right humerus, subsequent encounter for fracture with nonunion
S42392K	Other fracture of shaft of left humerus, subsequent encounter for fracture with nonunion
S42399K	Other fracture of shaft of unspecified humerus, subsequent encounter for fracture with nonunion
S42401K	Unspecified fracture of lower end of right humerus, subsequent encounter for fracture with nonunion
S42402K	Unspecified fracture of lower end of left humerus, subsequent encounter for fracture with nonunion

S42409K	Unspecified fracture of lower end of unspecified humerus, subsequent encounter for fracture with nonunion
S42411K	Displaced simple supracondylar fracture without intercondylar fracture of right humerus, subsequent encounter for fracture with nonunion
S42412K	Displaced simple supracondylar fracture without intercondylar fracture of left humerus, subsequent encounter for fracture with nonunion
S42413K	Displaced simple supracondylar fracture without intercondylar fracture of unspecified humerus, subsequent encounter for fracture with nonunion
S42414K	Nondisplaced simple supracondylar fracture without intercondylar fracture of right humerus, subsequent encounter for fracture with nonunion
S42415K	Nondisplaced simple supracondylar fracture without intercondylar fracture of left humerus, subsequent encounter for fracture with nonunion
S42416K	Nondisplaced simple supracondylar fracture without intercondylar fracture of unspecified humerus, subsequent encounter for fracture with nonunion
S42421K	Displaced comminuted supracondylar fracture without intercondylar fracture of right humerus, subsequent encounter for fracture with nonunion
S42422K	Displaced comminuted supracondylar fracture without intercondylar fracture of left humerus, subsequent encounter for fracture with nonunion
S42423K	Displaced comminuted supracondylar fracture without intercondylar fracture of unspecified humerus, subsequent encounter for fracture with nonunion
S42424K	Nondisplaced comminuted supracondylar fracture without intercondylar fracture of right humerus, subsequent encounter for fracture with nonunion
S42425K	Nondisplaced comminuted supracondylar fracture without intercondylar fracture of left humerus, subsequent encounter for fracture with nonunion
S42426K	Nondisplaced comminuted supracondylar fracture without intercondylar fracture of unspecified humerus, subsequent encounter for fracture with nonunion
S42431K	Displaced fracture (avulsion) of lateral epicondyle of right humerus, subsequent encounter for fracture with nonunion
S42432K	Displaced fracture (avulsion) of lateral epicondyle of left humerus, subsequent encounter for fracture with nonunion
S42433K	Displaced fracture (avulsion) of lateral epicondyle of unspecified humerus, subsequent encounter for fracture with nonunion

S42434K	Nondisplaced fracture (avulsion) of lateral epicondyle of right humerus, subsequent encounter for fracture with nonunion
S42435K	Nondisplaced fracture (avulsion) of lateral epicondyle of left humerus, subsequent encounter for fracture with nonunion
S42436K	Nondisplaced fracture (avulsion) of lateral epicondyle of unspecified humerus, subsequent encounter for fracture with nonunion
S42441K	Displaced fracture (avulsion) of medial epicondyle of right humerus, subsequent encounter for fracture with nonunion
S42442K	Displaced fracture (avulsion) of medial epicondyle of left humerus, subsequent encounter for fracture with nonunion
S42443K	Displaced fracture (avulsion) of medial epicondyle of unspecified humerus, subsequent encounter for fracture with nonunion
S42444K	Nondisplaced fracture (avulsion) of medial epicondyle of right humerus, subsequent encounter for fracture with nonunion
S42445K	Nondisplaced fracture (avulsion) of medial epicondyle of left humerus, subsequent encounter for fracture with nonunion
S42446K	Nondisplaced fracture (avulsion) of medial epicondyle of unspecified humerus, subsequent encounter for fracture with nonunion
S42447K	Incarcerated fracture (avulsion) of medial epicondyle of right humerus, subsequent encounter for fracture with nonunion
S42448K	Incarcerated fracture (avulsion) of medial epicondyle of left humerus, subsequent encounter for fracture with nonunion
S42449K	Incarcerated fracture (avulsion) of medial epicondyle of unspecified humerus, subsequent encounter for fracture with nonunion
S42451K	Displaced fracture of lateral condyle of right humerus, subsequent encounter for fracture with nonunion
S42452K	Displaced fracture of lateral condyle of left humerus, subsequent encounter for fracture with nonunion
S42453K	Displaced fracture of lateral condyle of unspecified humerus, subsequent encounter for fracture with nonunion
S42454K	Nondisplaced fracture of lateral condyle of right humerus, subsequent encounter for fracture with nonunion

S42455K	Nondisplaced fracture of lateral condyle of left humerus, subsequent encounter for fracture with nonunion
S42456K	Nondisplaced fracture of lateral condyle of unspecified humerus, subsequent encounter for fracture with nonunion
S42461K	Displaced fracture of medial condyle of right humerus, subsequent encounter for fracture with nonunion
S42462K	Displaced fracture of medial condyle of left humerus, subsequent encounter for fracture with nonunion
S42463K	Displaced fracture of medial condyle of unspecified humerus, subsequent encounter for fracture with nonunion
S42464K	Nondisplaced fracture of medial condyle of right humerus, subsequent encounter for fracture with nonunion
S42465K	Nondisplaced fracture of medial condyle of left humerus, subsequent encounter for fracture with nonunion
S42466K	Nondisplaced fracture of medial condyle of unspecified humerus, subsequent encounter for fracture with nonunion
S42471K	Displaced transcondylar fracture of right humerus, subsequent encounter for fracture with nonunion
S42472K	Displaced transcondylar fracture of left humerus, subsequent encounter for fracture with nonunion
S42473K	Displaced transcondylar fracture of unspecified humerus, subsequent encounter for fracture with nonunion
S42474K	Nondisplaced transcondylar fracture of right humerus, subsequent encounter for fracture with nonunion
S42475K	Nondisplaced transcondylar fracture of left humerus, subsequent encounter for fracture with nonunion
S42476K	Nondisplaced transcondylar fracture of unspecified humerus, subsequent encounter for fracture with nonunion
S42481K	Torus fracture of lower end of right humerus, subsequent encounter for fracture with nonunion
S42482K	Torus fracture of lower end of left humerus, subsequent encounter for fracture with nonunion

S42489K	Torus fracture of lower end of unspecified humerus, subsequent encounter for fracture with nonunion
S42491K	Other displaced fracture of lower end of right humerus, subsequent encounter for fracture with nonunion
S42492K	Other displaced fracture of lower end of left humerus, subsequent encounter for fracture with nonunion
S42493K	Other displaced fracture of lower end of unspecified humerus, subsequent encounter for fracture with nonunion
S42494K	Other nondisplaced fracture of lower end of right humerus, subsequent encounter for fracture with nonunion
S42495K	Other nondisplaced fracture of lower end of left humerus, subsequent encounter for fracture with nonunion
S42496K	Other nondisplaced fracture of lower end of unspecified humerus, subsequent encounter for fracture with nonunion
S4290XK	Fracture of unspecified shoulder girdle, part unspecified, subsequent encounter for fracture with nonunion
S4291XK	Fracture of right shoulder girdle, part unspecified, subsequent encounter for fracture with nonunion
S4292XK	Fracture of left shoulder girdle, part unspecified, subsequent encounter for fracture with nonunion
S49001K	Unspecified physeal fracture of upper end of humerus, right arm, subsequent encounter for fracture with nonunion
S49002K	Unspecified physeal fracture of upper end of humerus, left arm, subsequent encounter for fracture with nonunion
S49009K	Unspecified physeal fracture of upper end of humerus, unspecified arm, subsequent encounter for fracture with nonunion
S49011K	Salter-Harris Type I physeal fracture of upper end of humerus, right arm, subsequent encounter for fracture with nonunion
S49012K	Salter-Harris Type I physeal fracture of upper end of humerus, left arm, subsequent encounter for fracture with nonunion
S49019K	Salter-Harris Type I physeal fracture of upper end of humerus, unspecified arm, subsequent encounter for fracture with nonunion

S49021K	Salter-Harris Type II physeal fracture of upper end of humerus, right arm, subsequent encounter for fracture with nonunion
S49022K	Salter-Harris Type II physeal fracture of upper end of humerus, left arm, subsequent encounter for fracture with nonunion
S49029K	Salter-Harris Type II physeal fracture of upper end of humerus, unspecified arm, subsequent encounter for fracture with nonunion
S49031K	Salter-Harris Type III physeal fracture of upper end of humerus, right arm, subsequent encounter for fracture with nonunion
S49032K	Salter-Harris Type III physeal fracture of upper end of humerus, left arm, subsequent encounter for fracture with nonunion
S49039K	Salter-Harris Type III physeal fracture of upper end of humerus, unspecified arm, subsequent encounter for fracture with nonunion
S49041K	Salter-Harris Type IV physeal fracture of upper end of humerus, right arm, subsequent encounter for fracture with nonunion
S49042K	Salter-Harris Type IV physeal fracture of upper end of humerus, left arm, subsequent encounter for fracture with nonunion
S49049K	Salter-Harris Type IV physeal fracture of upper end of humerus, unspecified arm, subsequent encounter for fracture with nonunion
S49091K	Other physeal fracture of upper end of humerus, right arm, subsequent encounter for fracture with nonunion
S49092K	Other physeal fracture of upper end of humerus, left arm, subsequent encounter for fracture with nonunion
S49099K	Other physeal fracture of upper end of humerus, unspecified arm, subsequent encounter for fracture with nonunion
S49101K	Unspecified physeal fracture of lower end of humerus, right arm, subsequent encounter for fracture with nonunion
S49102K	Unspecified physeal fracture of lower end of humerus, left arm, subsequent encounter for fracture with nonunion
S49109K	Unspecified physeal fracture of lower end of humerus, unspecified arm, subsequent encounter for fracture with nonunion
S49111K	Salter-Harris Type I physeal fracture of lower end of humerus, right arm, subsequent encounter for fracture with nonunion

S49112K	Salter-Harris Type I physeal fracture of lower end of humerus, left arm, subsequent encounter for fracture with nonunion
S49119K	Salter-Harris Type I physeal fracture of lower end of humerus, unspecified arm, subsequent encounter for fracture with nonunion
S49121K	Salter-Harris Type II physeal fracture of lower end of humerus, right arm, subsequent encounter for fracture with nonunion
S49122K	Salter-Harris Type II physeal fracture of lower end of humerus, left arm, subsequent encounter for fracture with nonunion
S49129K	Salter-Harris Type II physeal fracture of lower end of humerus, unspecified arm, subsequent encounter for fracture with nonunion
S49131K	Salter-Harris Type III physeal fracture of lower end of humerus, right arm, subsequent encounter for fracture with nonunion
S49132K	Salter-Harris Type III physeal fracture of lower end of humerus, left arm, subsequent encounter for fracture with nonunion
S49139K	Salter-Harris Type III physeal fracture of lower end of humerus, unspecified arm, subsequent encounter for fracture with nonunion
S49141K	Salter-Harris Type IV physeal fracture of lower end of humerus, right arm, subsequent encounter for fracture with nonunion
S49142K	Salter-Harris Type IV physeal fracture of lower end of humerus, left arm, subsequent encounter for fracture with nonunion
S49149K	Salter-Harris Type IV physeal fracture of lower end of humerus, unspecified arm, subsequent encounter for fracture with nonunion
S49191K	Other physeal fracture of lower end of humerus, right arm, subsequent encounter for fracture with nonunion
S49192K	Other physeal fracture of lower end of humerus, left arm, subsequent encounter for fracture with nonunion
S49199K	Other physeal fracture of lower end of humerus, unspecified arm, subsequent encounter for fracture with nonunion
**Ulna**	
S52001K	Unspecified fracture of upper end of right ulna, subsequent encounter for closed fracture with nonunion
S52001M	Unspecified fracture of upper end of right ulna, subsequent encounter for open fracture type I or II with nonunion

S52001N	Unspecified fracture of upper end of right ulna, subsequent encounter for open fracture type IIIA, IIIB, or IIIC with nonunion
S52002K	Unspecified fracture of upper end of left ulna, subsequent encounter for closed fracture with nonunion
S52002M	Unspecified fracture of upper end of left ulna, subsequent encounter for open fracture type I or II with nonunion
S52002N	Unspecified fracture of upper end of left ulna, subsequent encounter for open fracture type IIIA, IIIB, or IIIC with nonunion
S52009K	Unspecified fracture of upper end of unspecified ulna, subsequent encounter for closed fracture with nonunion
S52009M	Unspecified fracture of upper end of unspecified ulna, subsequent encounter for open fracture type I or II with nonunion
S52009N	Unspecified fracture of upper end of unspecified ulna, subsequent encounter for open fracture type IIIA, IIIB, or IIIC with nonunion
S52011K	Torus fracture of upper end of right ulna, subsequent encounter for fracture with nonunion
S52012K	Torus fracture of upper end of left ulna, subsequent encounter for fracture with nonunion
S52019K	Torus fracture of upper end of unspecified ulna, subsequent encounter for fracture with nonunion
S52021K	Displaced fracture of olecranon process without intraarticular extension of right ulna, subsequent encounter for closed fracture with nonunion
S52021M	Displaced fracture of olecranon process without intraarticular extension of right ulna, subsequent encounter for open fracture type I or II with nonunion
S52021N	Displaced fracture of olecranon process without intraarticular extension of right ulna, subsequent encounter for open fracture type IIIA, IIIB, or IIIC with nonunion
S52022K	Displaced fracture of olecranon process without intraarticular extension of left ulna, subsequent encounter for closed fracture with nonunion
S52022M	Displaced fracture of olecranon process without intraarticular extension of left ulna, subsequent encounter for open fracture type I or II with nonunion

S52022N	Displaced fracture of olecranon process without intraarticular extension of left ulna, subsequent encounter for open fracture type IIIA, IIIB, or IIIC with nonunion
S52023K	Displaced fracture of olecranon process without intraarticular extension of unspecified ulna, subsequent encounter for closed fracture with nonunion
S52023M	Displaced fracture of olecranon process without intraarticular extension of unspecified ulna, subsequent encounter for open fracture type I or II with nonunion
S52023N	Displaced fracture of olecranon process without intraarticular extension of unspecified ulna, subsequent encounter for open fracture type IIIA, IIIB, or IIIC with nonunion
S52024K	Nondisplaced fracture of olecranon process without intraarticular extension of right ulna, subsequent encounter for closed fracture with nonunion
S52024M	Nondisplaced fracture of olecranon process without intraarticular extension of right ulna, subsequent encounter for open fracture type I or II with nonunion
S52024N	Nondisplaced fracture of olecranon process without intraarticular extension of right ulna, subsequent encounter for open fracture type IIIA, IIIB, or IIIC with nonunion
S52025K	Nondisplaced fracture of olecranon process without intraarticular extension of left ulna, subsequent encounter for closed fracture with nonunion
S52025M	Nondisplaced fracture of olecranon process without intraarticular extension of left ulna, subsequent encounter for open fracture type I or II with nonunion
S52025N	Nondisplaced fracture of olecranon process without intraarticular extension of left ulna, subsequent encounter for open fracture type IIIA, IIIB, or IIIC with nonunion
S52026K	Nondisplaced fracture of olecranon process without intraarticular extension of unspecified ulna, subsequent encounter for closed fracture with nonunion
S52026M	Nondisplaced fracture of olecranon process without intraarticular extension of unspecified ulna, subsequent encounter for open fracture type I or II with nonunion
S52026N	Nondisplaced fracture of olecranon process without intraarticular extension of unspecified ulna, subsequent encounter for open fracture type IIIA, IIIB, or IIIC with nonunion

S52031K	Displaced fracture of olecranon process with intraarticular extension of right ulna, subsequent encounter for closed fracture with nonunion
S52031M	Displaced fracture of olecranon process with intraarticular extension of right ulna, subsequent encounter for open fracture type I or II with nonunion
S52031N	Displaced fracture of olecranon process with intraarticular extension of right ulna, subsequent encounter for open fracture type IIIA, IIIB, or IIIC with nonunion
S52032K	Displaced fracture of olecranon process with intraarticular extension of left ulna, subsequent encounter for closed fracture with nonunion
S52032M	Displaced fracture of olecranon process with intraarticular extension of left ulna, subsequent encounter for open fracture type I or II with nonunion
S52032N	Displaced fracture of olecranon process with intraarticular extension of left ulna, subsequent encounter for open fracture type IIIA, IIIB, or IIIC with nonunion
S52033K	Displaced fracture of olecranon process with intraarticular extension of unspecified ulna, subsequent encounter for closed fracture with nonunion
S52033M	Displaced fracture of olecranon process with intraarticular extension of unspecified ulna, subsequent encounter for open fracture type I or II with nonunion
S52033N	Displaced fracture of olecranon process with intraarticular extension of unspecified ulna, subsequent encounter for open fracture type IIIA, IIIB, or IIIC with nonunion
S52034K	Nondisplaced fracture of olecranon process with intraarticular extension of right ulna, subsequent encounter for closed fracture with nonunion
S52034M	Nondisplaced fracture of olecranon process with intraarticular extension of right ulna, subsequent encounter for open fracture type I or II with nonunion
S52034N	Nondisplaced fracture of olecranon process with intraarticular extension of right ulna, subsequent encounter for open fracture type IIIA, IIIB, or IIIC with nonunion
S52035K	Nondisplaced fracture of olecranon process with intraarticular extension of left ulna, subsequent encounter for closed fracture with nonunion
S52035M	Nondisplaced fracture of olecranon process with intraarticular extension of left ulna, subsequent encounter for open fracture type I or II with nonunion

S52035N	Nondisplaced fracture of olecranon process with intraarticular extension of left ulna, subsequent encounter for open fracture type IIIA, IIIB, or IIIC with nonunion
S52036K	Nondisplaced fracture of olecranon process with intraarticular extension of unspecified ulna, subsequent encounter for closed fracture with nonunion
S52036M	Nondisplaced fracture of olecranon process with intraarticular extension of unspecified ulna, subsequent encounter for open fracture type I or II with nonunion
S52036N	Nondisplaced fracture of olecranon process with intraarticular extension of unspecified ulna, subsequent encounter for open fracture type IIIA, IIIB, or IIIC with nonunion
S52041K	Displaced fracture of coronoid process of right ulna, subsequent encounter for closed fracture with nonunion
S52041M	Displaced fracture of coronoid process of right ulna, subsequent encounter for open fracture type I or II with nonunion
S52041N	Displaced fracture of coronoid process of right ulna, subsequent encounter for open fracture type IIIA, IIIB, or IIIC with nonunion
S52042K	Displaced fracture of coronoid process of left ulna, subsequent encounter for closed fracture with nonunion
S52042M	Displaced fracture of coronoid process of left ulna, subsequent encounter for open fracture type I or II with nonunion
S52042N	Displaced fracture of coronoid process of left ulna, subsequent encounter for open fracture type IIIA, IIIB, or IIIC with nonunion
S52043K	Displaced fracture of coronoid process of unspecified ulna, subsequent encounter for closed fracture with nonunion
S52043M	Displaced fracture of coronoid process of unspecified ulna, subsequent encounter for open fracture type I or II with nonunion
S52043N	Displaced fracture of coronoid process of unspecified ulna, subsequent encounter for open fracture type IIIA, IIIB, or IIIC with nonunion
S52044K	Nondisplaced fracture of coronoid process of right ulna, subsequent encounter for closed fracture with nonunion
S52044M	Nondisplaced fracture of coronoid process of right ulna, subsequent encounter for open fracture type I or II with nonunion

S52044N	Nondisplaced fracture of coronoid process of right ulna, subsequent encounter for open fracture type IIIA, IIIB, or IIIC with nonunion
S52045K	Nondisplaced fracture of coronoid process of left ulna, subsequent encounter for closed fracture with nonunion
S52045M	Nondisplaced fracture of coronoid process of left ulna, subsequent encounter for open fracture type I or II with nonunion
S52045N	Nondisplaced fracture of coronoid process of left ulna, subsequent encounter for open fracture type IIIA, IIIB, or IIIC with nonunion
S52046K	Nondisplaced fracture of coronoid process of unspecified ulna, subsequent encounter for closed fracture with nonunion
S52046M	Nondisplaced fracture of coronoid process of unspecified ulna, subsequent encounter for open fracture type I or II with nonunion
S52046N	Nondisplaced fracture of coronoid process of unspecified ulna, subsequent encounter for open fracture type IIIA, IIIB, or IIIC with nonunion
S52091K	Other fracture of upper end of right ulna, subsequent encounter for closed fracture with nonunion
S52091M	Other fracture of upper end of right ulna, subsequent encounter for open fracture type I or II with nonunion
S52091N	Other fracture of upper end of right ulna, subsequent encounter for open fracture type IIIA, IIIB, or IIIC with nonunion
S52092K	Other fracture of upper end of left ulna, subsequent encounter for closed fracture with nonunion
S52092M	Other fracture of upper end of left ulna, subsequent encounter for open fracture type I or II with nonunion
S52092N	Other fracture of upper end of left ulna, subsequent encounter for open fracture type IIIA, IIIB, or IIIC with nonunion
S52099K	Other fracture of upper end of unspecified ulna, subsequent encounter for closed fracture with nonunion
S52099M	Other fracture of upper end of unspecified ulna, subsequent encounter for open fracture type I or II with nonunion
S52099N	Other fracture of upper end of unspecified ulna, subsequent encounter for open fracture type IIIA, IIIB, or IIIC with nonunion
**Radius**	

S52101K	Unspecified fracture of upper end of right radius, subsequent encounter for closed fracture with nonunion
S52101M	Unspecified fracture of upper end of right radius, subsequent encounter for open fracture type I or II with nonunion
S52101N	Unspecified fracture of upper end of right radius, subsequent encounter for open fracture type IIIA, IIIB, or IIIC with nonunion
S52102K	Unspecified fracture of upper end of left radius, subsequent encounter for closed fracture with nonunion
S52102M	Unspecified fracture of upper end of left radius, subsequent encounter for open fracture type I or II with nonunion
S52102N	Unspecified fracture of upper end of left radius, subsequent encounter for open fracture type IIIA, IIIB, or IIIC with nonunion
S52109K	Unspecified fracture of upper end of unspecified radius, subsequent encounter for closed fracture with nonunion
S52109M	Unspecified fracture of upper end of unspecified radius, subsequent encounter for open fracture type I or II with nonunion
S52109N	Unspecified fracture of upper end of unspecified radius, subsequent encounter for open fracture type IIIA, IIIB, or IIIC with nonunion
S52111K	Torus fracture of upper end of right radius, subsequent encounter for fracture with nonunion
S52112K	Torus fracture of upper end of left radius, subsequent encounter for fracture with nonunion
S52119K	Torus fracture of upper end of unspecified radius, subsequent encounter for fracture with nonunion
S52121K	Displaced fracture of head of right radius, subsequent encounter for closed fracture with nonunion
S52121M	Displaced fracture of head of right radius, subsequent encounter for open fracture type I or II with nonunion
S52121N	Displaced fracture of head of right radius, subsequent encounter for open fracture type IIIA, IIIB, or IIIC with nonunion
S52122K	Displaced fracture of head of left radius, subsequent encounter for closed fracture with nonunion

S52122M	Displaced fracture of head of left radius, subsequent encounter for open fracture type I or II with nonunion
S52122N	Displaced fracture of head of left radius, subsequent encounter for open fracture type IIIA, IIIB, or IIIC with nonunion
S52123K	Displaced fracture of head of unspecified radius, subsequent encounter for closed fracture with nonunion
S52123M	Displaced fracture of head of unspecified radius, subsequent encounter for open fracture type I or II with nonunion
S52123N	Displaced fracture of head of unspecified radius, subsequent encounter for open fracture type IIIA, IIIB, or IIIC with nonunion
S52124K	Nondisplaced fracture of head of right radius, subsequent encounter for closed fracture with nonunion
S52124M	Nondisplaced fracture of head of right radius, subsequent encounter for open fracture type I or II with nonunion
S52124N	Nondisplaced fracture of head of right radius, subsequent encounter for open fracture type IIIA, IIIB, or IIIC with nonunion
S52125K	Nondisplaced fracture of head of left radius, subsequent encounter for closed fracture with nonunion
S52125M	Nondisplaced fracture of head of left radius, subsequent encounter for open fracture type I or II with nonunion
S52125N	Nondisplaced fracture of head of left radius, subsequent encounter for open fracture type IIIA, IIIB, or IIIC with nonunion
S52126K	Nondisplaced fracture of head of unspecified radius, subsequent encounter for closed fracture with nonunion
S52126M	Nondisplaced fracture of head of unspecified radius, subsequent encounter for open fracture type I or II with nonunion
S52126N	Nondisplaced fracture of head of unspecified radius, subsequent encounter for open fracture type IIIA, IIIB, or IIIC with nonunion
S52131K	Displaced fracture of neck of right radius, subsequent encounter for closed fracture with nonunion
S52131M	Displaced fracture of neck of right radius, subsequent encounter for open fracture type I or II with nonunion

S52131N	Displaced fracture of neck of right radius, subsequent encounter for open fracture type IIIA, IIIB, or IIIC with nonunion
S52132K	Displaced fracture of neck of left radius, subsequent encounter for closed fracture with nonunion
S52132M	Displaced fracture of neck of left radius, subsequent encounter for open fracture type I or II with nonunion
S52132N	Displaced fracture of neck of left radius, subsequent encounter for open fracture type IIIA, IIIB, or IIIC with nonunion
S52133K	Displaced fracture of neck of unspecified radius, subsequent encounter for closed fracture with nonunion
S52133M	Displaced fracture of neck of unspecified radius, subsequent encounter for open fracture type I or II with nonunion
S52133N	Displaced fracture of neck of unspecified radius, subsequent encounter for open fracture type IIIA, IIIB, or IIIC with nonunion
S52134K	Nondisplaced fracture of neck of right radius, subsequent encounter for closed fracture with nonunion
S52134M	Nondisplaced fracture of neck of right radius, subsequent encounter for open fracture type I or II with nonunion
S52134N	Nondisplaced fracture of neck of right radius, subsequent encounter for open fracture type IIIA, IIIB, or IIIC with nonunion
S52135K	Nondisplaced fracture of neck of left radius, subsequent encounter for closed fracture with nonunion
S52135M	Nondisplaced fracture of neck of left radius, subsequent encounter for open fracture type I or II with nonunion
S52135N	Nondisplaced fracture of neck of left radius, subsequent encounter for open fracture type IIIA, IIIB, or IIIC with nonunion
S52136K	Nondisplaced fracture of neck of unspecified radius, subsequent encounter for closed fracture with nonunion
S52136M	Nondisplaced fracture of neck of unspecified radius, subsequent encounter for open fracture type I or II with nonunion
S52136N	Nondisplaced fracture of neck of unspecified radius, subsequent encounter for open fracture type IIIA, IIIB, or IIIC with nonunion

S52181K	Other fracture of upper end of right radius, subsequent encounter for closed fracture with nonunion
S52181M	Other fracture of upper end of right radius, subsequent encounter for open fracture type I or II with nonunion
S52181N	Other fracture of upper end of right radius, subsequent encounter for open fracture type IIIA, IIIB, or IIIC with nonunion
S52182K	Other fracture of upper end of left radius, subsequent encounter for closed fracture with nonunion
S52182M	Other fracture of upper end of left radius, subsequent encounter for open fracture type I or II with nonunion
S52182N	Other fracture of upper end of left radius, subsequent encounter for open fracture type IIIA, IIIB, or IIIC with nonunion
S52189K	Other fracture of upper end of unspecified radius, subsequent encounter for closed fracture with nonunion
S52189M	Other fracture of upper end of unspecified radius, subsequent encounter for open fracture type I or II with nonunion
S52189N	Other fracture of upper end of unspecified radius, subsequent encounter for open fracture type IIIA, IIIB, or IIIC with nonunion
**Ulna**	
S52201K	Unspecified fracture of shaft of right ulna, subsequent encounter for closed fracture with nonunion
S52201M	Unspecified fracture of shaft of right ulna, subsequent encounter for open fracture type I or II with nonunion
S52201N	Unspecified fracture of shaft of right ulna, subsequent encounter for open fracture type IIIA, IIIB, or IIIC with nonunion
S52202K	Unspecified fracture of shaft of left ulna, subsequent encounter for closed fracture with nonunion
S52202M	Unspecified fracture of shaft of left ulna, subsequent encounter for open fracture type I or II with nonunion
S52202N	Unspecified fracture of shaft of left ulna, subsequent encounter for open fracture type IIIA, IIIB, or IIIC with nonunion
S52209K	Unspecified fracture of shaft of unspecified ulna, subsequent encounter for closed fracture with nonunion

S52209M	Unspecified fracture of shaft of unspecified ulna, subsequent encounter for open fracture type I or II with nonunion
S52209N	Unspecified fracture of shaft of unspecified ulna, subsequent encounter for open fracture type IIIA, IIIB, or IIIC with nonunion
S52211K	Greenstick fracture of shaft of right ulna, subsequent encounter for fracture with nonunion
S52212K	Greenstick fracture of shaft of left ulna, subsequent encounter for fracture with nonunion
S52219K	Greenstick fracture of shaft of unspecified ulna, subsequent encounter for fracture with nonunion
S52221K	Displaced transverse fracture of shaft of right ulna, subsequent encounter for closed fracture with nonunion
S52221M	Displaced transverse fracture of shaft of right ulna, subsequent encounter for open fracture type I or II with nonunion
S52221N	Displaced transverse fracture of shaft of right ulna, subsequent encounter for open fracture type IIIA, IIIB, or IIIC with nonunion
S52222K	Displaced transverse fracture of shaft of left ulna, subsequent encounter for closed fracture with nonunion
S52222M	Displaced transverse fracture of shaft of left ulna, subsequent encounter for open fracture type I or II with nonunion
S52222N	Displaced transverse fracture of shaft of left ulna, subsequent encounter for open fracture type IIIA, IIIB, or IIIC with nonunion
S52223K	Displaced transverse fracture of shaft of unspecified ulna, subsequent encounter for closed fracture with nonunion
S52223M	Displaced transverse fracture of shaft of unspecified ulna, subsequent encounter for open fracture type I or II with nonunion
S52223N	Displaced transverse fracture of shaft of unspecified ulna, subsequent encounter for open fracture type IIIA, IIIB, or IIIC with nonunion
S52224K	Nondisplaced transverse fracture of shaft of right ulna, subsequent encounter for closed fracture with nonunion
S52224M	Nondisplaced transverse fracture of shaft of right ulna, subsequent encounter for open fracture type I or II with nonunion

S52224N	Nondisplaced transverse fracture of shaft of right ulna, subsequent encounter for open fracture type IIIA, IIIB, or IIIC with nonunion
S52225K	Nondisplaced transverse fracture of shaft of left ulna, subsequent encounter for closed fracture with nonunion
S52225M	Nondisplaced transverse fracture of shaft of left ulna, subsequent encounter for open fracture type I or II with nonunion
S52225N	Nondisplaced transverse fracture of shaft of left ulna, subsequent encounter for open fracture type IIIA, IIIB, or IIIC with nonunion
S52226K	Nondisplaced transverse fracture of shaft of unspecified ulna, subsequent encounter for closed fracture with nonunion
S52226M	Nondisplaced transverse fracture of shaft of unspecified ulna, subsequent encounter for open fracture type I or II with nonunion
S52226N	Nondisplaced transverse fracture of shaft of unspecified ulna, subsequent encounter for open fracture type IIIA, IIIB, or IIIC with nonunion
S52231K	Displaced oblique fracture of shaft of right ulna, subsequent encounter for closed fracture with nonunion
S52231M	Displaced oblique fracture of shaft of right ulna, subsequent encounter for open fracture type I or II with nonunion
S52231N	Displaced oblique fracture of shaft of right ulna, subsequent encounter for open fracture type IIIA, IIIB, or IIIC with nonunion
S52232K	Displaced oblique fracture of shaft of left ulna, subsequent encounter for closed fracture with nonunion
S52232M	Displaced oblique fracture of shaft of left ulna, subsequent encounter for open fracture type I or II with nonunion
S52232N	Displaced oblique fracture of shaft of left ulna, subsequent encounter for open fracture type IIIA, IIIB, or IIIC with nonunion
S52233K	Displaced oblique fracture of shaft of unspecified ulna, subsequent encounter for closed fracture with nonunion
S52233M	Displaced oblique fracture of shaft of unspecified ulna, subsequent encounter for open fracture type I or II with nonunion
S52233N	Displaced oblique fracture of shaft of unspecified ulna, subsequent encounter for open fracture type IIIA, IIIB, or IIIC with nonunion

S52234K	Nondisplaced oblique fracture of shaft of right ulna, subsequent encounter for closed fracture with nonunion
S52234M	Nondisplaced oblique fracture of shaft of right ulna, subsequent encounter for open fracture type I or II with nonunion
S52234N	Nondisplaced oblique fracture of shaft of right ulna, subsequent encounter for open fracture type IIIA, IIIB, or IIIC with nonunion
S52235K	Nondisplaced oblique fracture of shaft of left ulna, subsequent encounter for closed fracture with nonunion
S52235M	Nondisplaced oblique fracture of shaft of left ulna, subsequent encounter for open fracture type I or II with nonunion
S52235N	Nondisplaced oblique fracture of shaft of left ulna, subsequent encounter for open fracture type IIIA, IIIB, or IIIC with nonunion
S52236K	Nondisplaced oblique fracture of shaft of unspecified ulna, subsequent encounter for closed fracture with nonunion
S52236M	Nondisplaced oblique fracture of shaft of unspecified ulna, subsequent encounter for open fracture type I or II with nonunion
S52236N	Nondisplaced oblique fracture of shaft of unspecified ulna, subsequent encounter for open fracture type IIIA, IIIB, or IIIC with nonunion
S52241K	Displaced spiral fracture of shaft of ulna, right arm, subsequent encounter for closed fracture with nonunion
S52241M	Displaced spiral fracture of shaft of ulna, right arm, subsequent encounter for open fracture type I or II with nonunion
S52241N	Displaced spiral fracture of shaft of ulna, right arm, subsequent encounter for open fracture type IIIA, IIIB, or IIIC with nonunion
S52242K	Displaced spiral fracture of shaft of ulna, left arm, subsequent encounter for closed fracture with nonunion
S52242M	Displaced spiral fracture of shaft of ulna, left arm, subsequent encounter for open fracture type I or II with nonunion
S52242N	Displaced spiral fracture of shaft of ulna, left arm, subsequent encounter for open fracture type IIIA, IIIB, or IIIC with nonunion
S52243K	Displaced spiral fracture of shaft of ulna, unspecified arm, subsequent encounter for closed fracture with nonunion

S52243M	Displaced spiral fracture of shaft of ulna, unspecified arm, subsequent encounter for open fracture type I or II with nonunion
S52243N	Displaced spiral fracture of shaft of ulna, unspecified arm, subsequent encounter for open fracture type IIIA, IIIB, or IIIC with nonunion
S52244K	Nondisplaced spiral fracture of shaft of ulna, right arm, subsequent encounter for closed fracture with nonunion
S52244M	Nondisplaced spiral fracture of shaft of ulna, right arm, subsequent encounter for open fracture type I or II with nonunion
S52244N	Nondisplaced spiral fracture of shaft of ulna, right arm, subsequent encounter for open fracture type IIIA, IIIB, or IIIC with nonunion
S52245K	Nondisplaced spiral fracture of shaft of ulna, left arm, subsequent encounter for closed fracture with nonunion
S52245M	Nondisplaced spiral fracture of shaft of ulna, left arm, subsequent encounter for open fracture type I or II with nonunion
S52245N	Nondisplaced spiral fracture of shaft of ulna, left arm, subsequent encounter for open fracture type IIIA, IIIB, or IIIC with nonunion
S52246K	Nondisplaced spiral fracture of shaft of ulna, unspecified arm, subsequent encounter for closed fracture with nonunion
S52246M	Nondisplaced spiral fracture of shaft of ulna, unspecified arm, subsequent encounter for open fracture type I or II with nonunion
S52246N	Nondisplaced spiral fracture of shaft of ulna, unspecified arm, subsequent encounter for open fracture type IIIA, IIIB, or IIIC with nonunion
S52251K	Displaced comminuted fracture of shaft of ulna, right arm, subsequent encounter for closed fracture with nonunion
S52251M	Displaced comminuted fracture of shaft of ulna, right arm, subsequent encounter for open fracture type I or II with nonunion
S52251N	Displaced comminuted fracture of shaft of ulna, right arm, subsequent encounter for open fracture type IIIA, IIIB, or IIIC with nonunion
S52252K	Displaced comminuted fracture of shaft of ulna, left arm, subsequent encounter for closed fracture with nonunion
S52252M	Displaced comminuted fracture of shaft of ulna, left arm, subsequent encounter for open fracture type I or II with nonunion

S52252N	Displaced comminuted fracture of shaft of ulna, left arm, subsequent encounter for open fracture type IIIA, IIIB, or IIIC with nonunion
S52253K	Displaced comminuted fracture of shaft of ulna, unspecified arm, subsequent encounter for closed fracture with nonunion
S52253M	Displaced comminuted fracture of shaft of ulna, unspecified arm, subsequent encounter for open fracture type I or II with nonunion
S52253N	Displaced comminuted fracture of shaft of ulna, unspecified arm, subsequent encounter for open fracture type IIIA, IIIB, or IIIC with nonunion
S52254K	Nondisplaced comminuted fracture of shaft of ulna, right arm, subsequent encounter for closed fracture with nonunion
S52254M	Nondisplaced comminuted fracture of shaft of ulna, right arm, subsequent encounter for open fracture type I or II with nonunion
S52254N	Nondisplaced comminuted fracture of shaft of ulna, right arm, subsequent encounter for open fracture type IIIA, IIIB, or IIIC with nonunion
S52255K	Nondisplaced comminuted fracture of shaft of ulna, left arm, subsequent encounter for closed fracture with nonunion
S52255M	Nondisplaced comminuted fracture of shaft of ulna, left arm, subsequent encounter for open fracture type I or II with nonunion
S52255N	Nondisplaced comminuted fracture of shaft of ulna, left arm, subsequent encounter for open fracture type IIIA, IIIB, or IIIC with nonunion
S52256K	Nondisplaced comminuted fracture of shaft of ulna, unspecified arm, subsequent encounter for closed fracture with nonunion
S52256M	Nondisplaced comminuted fracture of shaft of ulna, unspecified arm, subsequent encounter for open fracture type I or II with nonunion
S52256N	Nondisplaced comminuted fracture of shaft of ulna, unspecified arm, subsequent encounter for open fracture type IIIA, IIIB, or IIIC with nonunion
S52261K	Displaced segmental fracture of shaft of ulna, right arm, subsequent encounter for closed fracture with nonunion
S52261M	Displaced segmental fracture of shaft of ulna, right arm, subsequent encounter for open fracture type I or II with nonunion
S52261N	Displaced segmental fracture of shaft of ulna, right arm, subsequent encounter for open fracture type IIIA, IIIB, or IIIC with nonunion

S52262K	Displaced segmental fracture of shaft of ulna, left arm, subsequent encounter for closed fracture with nonunion
S52262M	Displaced segmental fracture of shaft of ulna, left arm, subsequent encounter for open fracture type I or II with nonunion
S52262N	Displaced segmental fracture of shaft of ulna, left arm, subsequent encounter for open fracture type IIIA, IIIB, or IIIC with nonunion
S52263K	Displaced segmental fracture of shaft of ulna, unspecified arm, subsequent encounter for closed fracture with nonunion
S52263M	Displaced segmental fracture of shaft of ulna, unspecified arm, subsequent encounter for open fracture type I or II with nonunion
S52263N	Displaced segmental fracture of shaft of ulna, unspecified arm, subsequent encounter for open fracture type IIIA, IIIB, or IIIC with nonunion
S52264K	Nondisplaced segmental fracture of shaft of ulna, right arm, subsequent encounter for closed fracture with nonunion
S52264M	Nondisplaced segmental fracture of shaft of ulna, right arm, subsequent encounter for open fracture type I or II with nonunion
S52264N	Nondisplaced segmental fracture of shaft of ulna, right arm, subsequent encounter for open fracture type IIIA, IIIB, or IIIC with nonunion
S52265K	Nondisplaced segmental fracture of shaft of ulna, left arm, subsequent encounter for closed fracture with nonunion
S52265M	Nondisplaced segmental fracture of shaft of ulna, left arm, subsequent encounter for open fracture type I or II with nonunion
S52265N	Nondisplaced segmental fracture of shaft of ulna, left arm, subsequent encounter for open fracture type IIIA, IIIB, or IIIC with nonunion
S52266K	Nondisplaced segmental fracture of shaft of ulna, unspecified arm, subsequent encounter for closed fracture with nonunion
S52266M	Nondisplaced segmental fracture of shaft of ulna, unspecified arm, subsequent encounter for open fracture type I or II with nonunion
S52266N	Nondisplaced segmental fracture of shaft of ulna, unspecified arm, subsequent encounter for open fracture type IIIA, IIIB, or IIIC with nonunion
S52271K	Monteggias fracture of right ulna, subsequent encounter for closed fracture with nonunion

S52271M	Monteggias fracture of right ulna, subsequent encounter for open fracture type I or II with nonunion
S52271N	Monteggias fracture of right ulna, subsequent encounter for open fracture type IIIA, IIIB, or IIIC with nonunion
S52272K	Monteggias fracture of left ulna, subsequent encounter for closed fracture with nonunion
S52272M	Monteggias fracture of left ulna, subsequent encounter for open fracture type I or II with nonunion
S52272N	Monteggias fracture of left ulna, subsequent encounter for open fracture type IIIA, IIIB, or IIIC with nonunion
S52279K	Monteggias fracture of unspecified ulna, subsequent encounter for closed fracture with nonunion
S52279M	Monteggias fracture of unspecified ulna, subsequent encounter for open fracture type I or II with nonunion
S52279N	Monteggias fracture of unspecified ulna, subsequent encounter for open fracture type IIIA, IIIB, or IIIC with nonunion
S52281K	Bent bone of right ulna, subsequent encounter for closed fracture with nonunion
S52281M	Bent bone of right ulna, subsequent encounter for open fracture type I or II with nonunion
S52281N	Bent bone of right ulna, subsequent encounter for open fracture type IIIA, IIIB, or IIIC with nonunion
S52282K	Bent bone of left ulna, subsequent encounter for closed fracture with nonunion
S52282M	Bent bone of left ulna, subsequent encounter for open fracture type I or II with nonunion
S52282N	Bent bone of left ulna, subsequent encounter for open fracture type IIIA, IIIB, or IIIC with nonunion
S52283K	Bent bone of unspecified ulna, subsequent encounter for closed fracture with nonunion
S52283M	Bent bone of unspecified ulna, subsequent encounter for open fracture type I or II with nonunion
S52283N	Bent bone of unspecified ulna, subsequent encounter for open fracture type IIIA, IIIB, or IIIC with nonunion

S52291K	Other fracture of shaft of right ulna, subsequent encounter for closed fracture with nonunion
S52291M	Other fracture of shaft of right ulna, subsequent encounter for open fracture type I or II with nonunion
S52291N	Other fracture of shaft of right ulna, subsequent encounter for open fracture type IIIA, IIIB, or IIIC with nonunion
S52292K	Other fracture of shaft of left ulna, subsequent encounter for closed fracture with nonunion
S52292M	Other fracture of shaft of left ulna, subsequent encounter for open fracture type I or II with nonunion
S52292N	Other fracture of shaft of left ulna, subsequent encounter for open fracture type IIIA, IIIB, or IIIC with nonunion
S52299K	Other fracture of shaft of unspecified ulna, subsequent encounter for closed fracture with nonunion
S52299M	Other fracture of shaft of unspecified ulna, subsequent encounter for open fracture type I or II with nonunion
S52299N	Other fracture of shaft of unspecified ulna, subsequent encounter for open fracture type IIIA, IIIB, or IIIC with nonunion
**Radius**	
S52301K	Unspecified fracture of shaft of right radius, subsequent encounter for closed fracture with nonunion
S52301M	Unspecified fracture of shaft of right radius, subsequent encounter for open fracture type I or II with nonunion
S52301N	Unspecified fracture of shaft of right radius, subsequent encounter for open fracture type IIIA, IIIB, or IIIC with nonunion
S52302K	Unspecified fracture of shaft of left radius, subsequent encounter for closed fracture with nonunion
S52302M	Unspecified fracture of shaft of left radius, subsequent encounter for open fracture type I or II with nonunion
S52302N	Unspecified fracture of shaft of left radius, subsequent encounter for open fracture type IIIA, IIIB, or IIIC with nonunion
S52309K	Unspecified fracture of shaft of unspecified radius, subsequent encounter for closed fracture with nonunion

S52309M	Unspecified fracture of shaft of unspecified radius, subsequent encounter for open fracture type I or II with nonunion
S52309N	Unspecified fracture of shaft of unspecified radius, subsequent encounter for open fracture type IIIA, IIIB, or IIIC with nonunion
S52311K	Greenstick fracture of shaft of radius, right arm, subsequent encounter for fracture with nonunion
S52312K	Greenstick fracture of shaft of radius, left arm, subsequent encounter for fracture with nonunion
S52319K	Greenstick fracture of shaft of radius, unspecified arm, subsequent encounter for fracture with nonunion
S52321K	Displaced transverse fracture of shaft of right radius, subsequent encounter for closed fracture with nonunion
S52321M	Displaced transverse fracture of shaft of right radius, subsequent encounter for open fracture type I or II with nonunion
S52321N	Displaced transverse fracture of shaft of right radius, subsequent encounter for open fracture type IIIA, IIIB, or IIIC with nonunion
S52322K	Displaced transverse fracture of shaft of left radius, subsequent encounter for closed fracture with nonunion
S52322M	Displaced transverse fracture of shaft of left radius, subsequent encounter for open fracture type I or II with nonunion
S52322N	Displaced transverse fracture of shaft of left radius, subsequent encounter for open fracture type IIIA, IIIB, or IIIC with nonunion
S52323K	Displaced transverse fracture of shaft of unspecified radius, subsequent encounter for closed fracture with nonunion
S52323M	Displaced transverse fracture of shaft of unspecified radius, subsequent encounter for open fracture type I or II with nonunion
S52323N	Displaced transverse fracture of shaft of unspecified radius, subsequent encounter for open fracture type IIIA, IIIB, or IIIC with nonunion
S52324K	Nondisplaced transverse fracture of shaft of right radius, subsequent encounter for closed fracture with nonunion
S52324M	Nondisplaced transverse fracture of shaft of right radius, subsequent encounter for open fracture type I or II with nonunion

S52324N	Nondisplaced transverse fracture of shaft of right radius, subsequent encounter for open fracture type IIIA, IIIB, or IIIC with nonunion
S52325K	Nondisplaced transverse fracture of shaft of left radius, subsequent encounter for closed fracture with nonunion
S52325M	Nondisplaced transverse fracture of shaft of left radius, subsequent encounter for open fracture type I or II with nonunion
S52325N	Nondisplaced transverse fracture of shaft of left radius, subsequent encounter for open fracture type IIIA, IIIB, or IIIC with nonunion
S52326K	Nondisplaced transverse fracture of shaft of unspecified radius, subsequent encounter for closed fracture with nonunion
S52326M	Nondisplaced transverse fracture of shaft of unspecified radius, subsequent encounter for open fracture type I or II with nonunion
S52326N	Nondisplaced transverse fracture of shaft of unspecified radius, subsequent encounter for open fracture type IIIA, IIIB, or IIIC with nonunion
S52331K	Displaced oblique fracture of shaft of right radius, subsequent encounter for closed fracture with nonunion
S52331M	Displaced oblique fracture of shaft of right radius, subsequent encounter for open fracture type I or II with nonunion
S52331N	Displaced oblique fracture of shaft of right radius, subsequent encounter for open fracture type IIIA, IIIB, or IIIC with nonunion
S52332K	Displaced oblique fracture of shaft of left radius, subsequent encounter for closed fracture with nonunion
S52332M	Displaced oblique fracture of shaft of left radius, subsequent encounter for open fracture type I or II with nonunion
S52332N	Displaced oblique fracture of shaft of left radius, subsequent encounter for open fracture type IIIA, IIIB, or IIIC with nonunion
S52333K	Displaced oblique fracture of shaft of unspecified radius, subsequent encounter for closed fracture with nonunion
S52333M	Displaced oblique fracture of shaft of unspecified radius, subsequent encounter for open fracture type I or II with nonunion
S52333N	Displaced oblique fracture of shaft of unspecified radius, subsequent encounter for open fracture type IIIA, IIIB, or IIIC with nonunion

S52334K	Nondisplaced oblique fracture of shaft of right radius, subsequent encounter for closed fracture with nonunion
S52334M	Nondisplaced oblique fracture of shaft of right radius, subsequent encounter for open fracture type I or II with nonunion
S52334N	Nondisplaced oblique fracture of shaft of right radius, subsequent encounter for open fracture type IIIA, IIIB, or IIIC with nonunion
S52335K	Nondisplaced oblique fracture of shaft of left radius, subsequent encounter for closed fracture with nonunion
S52335M	Nondisplaced oblique fracture of shaft of left radius, subsequent encounter for open fracture type I or II with nonunion
S52335N	Nondisplaced oblique fracture of shaft of left radius, subsequent encounter for open fracture type IIIA, IIIB, or IIIC with nonunion
S52336K	Nondisplaced oblique fracture of shaft of unspecified radius, subsequent encounter for closed fracture with nonunion
S52336M	Nondisplaced oblique fracture of shaft of unspecified radius, subsequent encounter for open fracture type I or II with nonunion
S52336N	Nondisplaced oblique fracture of shaft of unspecified radius, subsequent encounter for open fracture type IIIA, IIIB, or IIIC with nonunion
S52341K	Displaced spiral fracture of shaft of radius, right arm, subsequent encounter for closed fracture with nonunion
S52341M	Displaced spiral fracture of shaft of radius, right arm, subsequent encounter for open fracture type I or II with nonunion
S52341N	Displaced spiral fracture of shaft of radius, right arm, subsequent encounter for open fracture type IIIA, IIIB, or IIIC with nonunion
S52342K	Displaced spiral fracture of shaft of radius, left arm, subsequent encounter for closed fracture with nonunion
S52342M	Displaced spiral fracture of shaft of radius, left arm, subsequent encounter for open fracture type I or II with nonunion
S52342N	Displaced spiral fracture of shaft of radius, left arm, subsequent encounter for open fracture type IIIA, IIIB, or IIIC with nonunion
S52343K	Displaced spiral fracture of shaft of radius, unspecified arm, subsequent encounter for closed fracture with nonunion

S52343M	Displaced spiral fracture of shaft of radius, unspecified arm, subsequent encounter for open fracture type I or II with nonunion
S52343N	Displaced spiral fracture of shaft of radius, unspecified arm, subsequent encounter for open fracture type IIIA, IIIB, or IIIC with nonunion
S52344K	Nondisplaced spiral fracture of shaft of radius, right arm, subsequent encounter for closed fracture with nonunion
S52344M	Nondisplaced spiral fracture of shaft of radius, right arm, subsequent encounter for open fracture type I or II with nonunion
S52344N	Nondisplaced spiral fracture of shaft of radius, right arm, subsequent encounter for open fracture type IIIA, IIIB, or IIIC with nonunion
S52345K	Nondisplaced spiral fracture of shaft of radius, left arm, subsequent encounter for closed fracture with nonunion
S52345M	Nondisplaced spiral fracture of shaft of radius, left arm, subsequent encounter for open fracture type I or II with nonunion
S52345N	Nondisplaced spiral fracture of shaft of radius, left arm, subsequent encounter for open fracture type IIIA, IIIB, or IIIC with nonunion
S52346K	Nondisplaced spiral fracture of shaft of radius, unspecified arm, subsequent encounter for closed fracture with nonunion
S52346M	Nondisplaced spiral fracture of shaft of radius, unspecified arm, subsequent encounter for open fracture type I or II with nonunion
S52346N	Nondisplaced spiral fracture of shaft of radius, unspecified arm, subsequent encounter for open fracture type IIIA, IIIB, or IIIC with nonunion
S52351K	Displaced comminuted fracture of shaft of radius, right arm, subsequent encounter for closed fracture with nonunion
S52351M	Displaced comminuted fracture of shaft of radius, right arm, subsequent encounter for open fracture type I or II with nonunion
S52351N	Displaced comminuted fracture of shaft of radius, right arm, subsequent encounter for open fracture type IIIA, IIIB, or IIIC with nonunion
S52352K	Displaced comminuted fracture of shaft of radius, left arm, subsequent encounter for closed fracture with nonunion
S52352M	Displaced comminuted fracture of shaft of radius, left arm, subsequent encounter for open fracture type I or II with nonunion

S52352N	Displaced comminuted fracture of shaft of radius, left arm, subsequent encounter for open fracture type IIIA, IIIB, or IIIC with nonunion
S52353K	Displaced comminuted fracture of shaft of radius, unspecified arm, subsequent encounter for closed fracture with nonunion
S52353M	Displaced comminuted fracture of shaft of radius, unspecified arm, subsequent encounter for open fracture type I or II with nonunion
S52353N	Displaced comminuted fracture of shaft of radius, unspecified arm, subsequent encounter for open fracture type IIIA, IIIB, or IIIC with nonunion
S52354K	Nondisplaced comminuted fracture of shaft of radius, right arm, subsequent encounter for closed fracture with nonunion
S52354M	Nondisplaced comminuted fracture of shaft of radius, right arm, subsequent encounter for open fracture type I or II with nonunion
S52354N	Nondisplaced comminuted fracture of shaft of radius, right arm, subsequent encounter for open fracture type IIIA, IIIB, or IIIC with nonunion
S52355K	Nondisplaced comminuted fracture of shaft of radius, left arm, subsequent encounter for closed fracture with nonunion
S52355M	Nondisplaced comminuted fracture of shaft of radius, left arm, subsequent encounter for open fracture type I or II with nonunion
S52355N	Nondisplaced comminuted fracture of shaft of radius, left arm, subsequent encounter for open fracture type IIIA, IIIB, or IIIC with nonunion
S52356K	Nondisplaced comminuted fracture of shaft of radius, unspecified arm, subsequent encounter for closed fracture with nonunion
S52356M	Nondisplaced comminuted fracture of shaft of radius, unspecified arm, subsequent encounter for open fracture type I or II with nonunion
S52356N	Nondisplaced comminuted fracture of shaft of radius, unspecified arm, subsequent encounter for open fracture type IIIA, IIIB, or IIIC with nonunion
S52361K	Displaced segmental fracture of shaft of radius, right arm, subsequent encounter for closed fracture with nonunion
S52361M	Displaced segmental fracture of shaft of radius, right arm, subsequent encounter for open fracture type I or II with nonunion
S52361N	Displaced segmental fracture of shaft of radius, right arm, subsequent encounter for open fracture type IIIA, IIIB, or IIIC with nonunion

S52362K	Displaced segmental fracture of shaft of radius, left arm, subsequent encounter for closed fracture with nonunion
S52362M	Displaced segmental fracture of shaft of radius, left arm, subsequent encounter for open fracture type I or II with nonunion
S52362N	Displaced segmental fracture of shaft of radius, left arm, subsequent encounter for open fracture type IIIA, IIIB, or IIIC with nonunion
S52363K	Displaced segmental fracture of shaft of radius, unspecified arm, subsequent encounter for closed fracture with nonunion
S52363M	Displaced segmental fracture of shaft of radius, unspecified arm, subsequent encounter for open fracture type I or II with nonunion
S52363N	Displaced segmental fracture of shaft of radius, unspecified arm, subsequent encounter for open fracture type IIIA, IIIB, or IIIC with nonunion
S52364K	Nondisplaced segmental fracture of shaft of radius, right arm, subsequent encounter for closed fracture with nonunion
S52364M	Nondisplaced segmental fracture of shaft of radius, right arm, subsequent encounter for open fracture type I or II with nonunion
S52364N	Nondisplaced segmental fracture of shaft of radius, right arm, subsequent encounter for open fracture type IIIA, IIIB, or IIIC with nonunion
S52365K	Nondisplaced segmental fracture of shaft of radius, left arm, subsequent encounter for closed fracture with nonunion
S52365M	Nondisplaced segmental fracture of shaft of radius, left arm, subsequent encounter for open fracture type I or II with nonunion
S52365N	Nondisplaced segmental fracture of shaft of radius, left arm, subsequent encounter for open fracture type IIIA, IIIB, or IIIC with nonunion
S52366K	Nondisplaced segmental fracture of shaft of radius, unspecified arm, subsequent encounter for closed fracture with nonunion
S52366M	Nondisplaced segmental fracture of shaft of radius, unspecified arm, subsequent encounter for open fracture type I or II with nonunion
S52366N	Nondisplaced segmental fracture of shaft of radius, unspecified arm, subsequent encounter for open fracture type IIIA, IIIB, or IIIC with nonunion
S52371K	Galeazzis fracture of right radius, subsequent encounter for closed fracture with nonunion

S52371M	Galeazzis fracture of right radius, subsequent encounter for open fracture type I or II with nonunion
S52371N	Galeazzis fracture of right radius, subsequent encounter for open fracture type IIIA, IIIB, or IIIC with nonunion
S52372K	Galeazzis fracture of left radius, subsequent encounter for closed fracture with nonunion
S52372M	Galeazzis fracture of left radius, subsequent encounter for open fracture type I or II with nonunion
S52372N	Galeazzis fracture of left radius, subsequent encounter for open fracture type IIIA, IIIB, or IIIC with nonunion
S52379K	Galeazzis fracture of unspecified radius, subsequent encounter for closed fracture with nonunion
S52379M	Galeazzis fracture of unspecified radius, subsequent encounter for open fracture type I or II with nonunion
S52379N	Galeazzis fracture of unspecified radius, subsequent encounter for open fracture type IIIA, IIIB, or IIIC with nonunion
S52381K	Bent bone of right radius, subsequent encounter for closed fracture with nonunion
S52381M	Bent bone of right radius, subsequent encounter for open fracture type I or II with nonunion
S52381N	Bent bone of right radius, subsequent encounter for open fracture type IIIA, IIIB, or IIIC with nonunion
S52382K	Bent bone of left radius, subsequent encounter for closed fracture with nonunion
S52382M	Bent bone of left radius, subsequent encounter for open fracture type I or II with nonunion
S52382N	Bent bone of left radius, subsequent encounter for open fracture type IIIA, IIIB, or IIIC with nonunion
S52389K	Bent bone of unspecified radius, subsequent encounter for closed fracture with nonunion
S52389M	Bent bone of unspecified radius, subsequent encounter for open fracture type I or II with nonunion

S52389N	Bent bone of unspecified radius, subsequent encounter for open fracture type IIIA, IIIB, or IIIC with nonunion
S52391K	Other fracture of shaft of radius, right arm, subsequent encounter for closed fracture with nonunion
S52391M	Other fracture of shaft of radius, right arm, subsequent encounter for open fracture type I or II with nonunion
S52391N	Other fracture of shaft of radius, right arm, subsequent encounter for open fracture type IIIA, IIIB, or IIIC with nonunion
S52392K	Other fracture of shaft of radius, left arm, subsequent encounter for closed fracture with nonunion
S52392M	Other fracture of shaft of radius, left arm, subsequent encounter for open fracture type I or II with nonunion
S52392N	Other fracture of shaft of radius, left arm, subsequent encounter for open fracture type IIIA, IIIB, or IIIC with nonunion
S52399K	Other fracture of shaft of radius, unspecified arm, subsequent encounter for closed fracture with nonunion
S52399M	Other fracture of shaft of radius, unspecified arm, subsequent encounter for open fracture type I or II with nonunion
S52399N	Other fracture of shaft of radius, unspecified arm, subsequent encounter for open fracture type IIIA, IIIB, or IIIC with nonunion
S52501K	Unspecified fracture of the lower end of right radius, subsequent encounter for closed fracture with nonunion
S52501M	Unspecified fracture of the lower end of right radius, subsequent encounter for open fracture type I or II with nonunion
S52501N	Unspecified fracture of the lower end of right radius, subsequent encounter for open fracture type IIIA, IIIB, or IIIC with nonunion
S52502K	Unspecified fracture of the lower end of left radius, subsequent encounter for closed fracture with nonunion
S52502M	Unspecified fracture of the lower end of left radius, subsequent encounter for open fracture type I or II with nonunion
S52502N	Unspecified fracture of the lower end of left radius, subsequent encounter for open fracture type IIIA, IIIB, or IIIC with nonunion

S52509K	Unspecified fracture of the lower end of unspecified radius, subsequent encounter for closed fracture with nonunion
S52509M	Unspecified fracture of the lower end of unspecified radius, subsequent encounter for open fracture type I or II with nonunion
S52509N	Unspecified fracture of the lower end of unspecified radius, subsequent encounter for open fracture type IIIA, IIIB, or IIIC with nonunion
S52511K	Displaced fracture of right radial styloid process, subsequent encounter for closed fracture with nonunion
S52511M	Displaced fracture of right radial styloid process, subsequent encounter for open fracture type I or II with nonunion
S52511N	Displaced fracture of right radial styloid process, subsequent encounter for open fracture type IIIA, IIIB, or IIIC with nonunion
S52512K	Displaced fracture of left radial styloid process, subsequent encounter for closed fracture with nonunion
S52512M	Displaced fracture of left radial styloid process, subsequent encounter for open fracture type I or II with nonunion
S52512N	Displaced fracture of left radial styloid process, subsequent encounter for open fracture type IIIA, IIIB, or IIIC with nonunion
S52513K	Displaced fracture of unspecified radial styloid process, subsequent encounter for closed fracture with nonunion
S52513M	Displaced fracture of unspecified radial styloid process, subsequent encounter for open fracture type I or II with nonunion
S52513N	Displaced fracture of unspecified radial styloid process, subsequent encounter for open fracture type IIIA, IIIB, or IIIC with nonunion
S52514K	Nondisplaced fracture of right radial styloid process, subsequent encounter for closed fracture with nonunion
S52514M	Nondisplaced fracture of right radial styloid process, subsequent encounter for open fracture type I or II with nonunion
S52514N	Nondisplaced fracture of right radial styloid process, subsequent encounter for open fracture type IIIA, IIIB, or IIIC with nonunion
S52515K	Nondisplaced fracture of left radial styloid process, subsequent encounter for closed fracture with nonunion

S52515M	Nondisplaced fracture of left radial styloid process, subsequent encounter for open fracture type I or II with nonunion
S52515N	Nondisplaced fracture of left radial styloid process, subsequent encounter for open fracture type IIIA, IIIB, or IIIC with nonunion
S52516K	Nondisplaced fracture of unspecified radial styloid process, subsequent encounter for closed fracture with nonunion
S52516M	Nondisplaced fracture of unspecified radial styloid process, subsequent encounter for open fracture type I or II with nonunion
S52516N	Nondisplaced fracture of unspecified radial styloid process, subsequent encounter for open fracture type IIIA, IIIB, or IIIC with nonunion
S52521K	Torus fracture of lower end of right radius, subsequent encounter for fracture with nonunion
S52522K	Torus fracture of lower end of left radius, subsequent encounter for fracture with nonunion
S52529K	Torus fracture of lower end of unspecified radius, subsequent encounter for fracture with nonunion
S52531K	Colles fracture of right radius, subsequent encounter for closed fracture with nonunion
S52531M	Colles fracture of right radius, subsequent encounter for open fracture type I or II with nonunion
S52531N	Colles fracture of right radius, subsequent encounter for open fracture type IIIA, IIIB, or IIIC with nonunion
S52532K	Colles fracture of left radius, subsequent encounter for closed fracture with nonunion
S52532M	Colles fracture of left radius, subsequent encounter for open fracture type I or II with nonunion
S52532N	Colles fracture of left radius, subsequent encounter for open fracture type IIIA, IIIB, or IIIC with nonunion
S52539K	Colles fracture of unspecified radius, subsequent encounter for closed fracture with nonunion
S52539M	Colles fracture of unspecified radius, subsequent encounter for open fracture type I or II with nonunion

S52539N	Colles fracture of unspecified radius, subsequent encounter for open fracture type IIIA, IIIB, or IIIC with nonunion
S52541K	Smiths fracture of right radius, subsequent encounter for closed fracture with nonunion
S52541M	Smiths fracture of right radius, subsequent encounter for open fracture type I or II with nonunion
S52541N	Smiths fracture of right radius, subsequent encounter for open fracture type IIIA, IIIB, or IIIC with nonunion
S52542K	Smiths fracture of left radius, subsequent encounter for closed fracture with nonunion
S52542M	Smiths fracture of left radius, subsequent encounter for open fracture type I or II with nonunion
S52542N	Smiths fracture of left radius, subsequent encounter for open fracture type IIIA, IIIB, or IIIC with nonunion
S52549K	Smiths fracture of unspecified radius, subsequent encounter for closed fracture with nonunion
S52549M	Smiths fracture of unspecified radius, subsequent encounter for open fracture type I or II with nonunion
S52549N	Smiths fracture of unspecified radius, subsequent encounter for open fracture type IIIA, IIIB, or IIIC with nonunion
S52551K	Other extraarticular fracture of lower end of right radius, subsequent encounter for closed fracture with nonunion
S52551M	Other extraarticular fracture of lower end of right radius, subsequent encounter for open fracture type I or II with nonunion
S52551N	Other extraarticular fracture of lower end of right radius, subsequent encounter for open fracture type IIIA, IIIB, or IIIC with nonunion
S52552K	Other extraarticular fracture of lower end of left radius, subsequent encounter for closed fracture with nonunion
S52552M	Other extraarticular fracture of lower end of left radius, subsequent encounter for open fracture type I or II with nonunion
S52552N	Other extraarticular fracture of lower end of left radius, subsequent encounter for open fracture type IIIA, IIIB, or IIIC with nonunion

S52559K	Other extraarticular fracture of lower end of unspecified radius, subsequent encounter for closed fracture with nonunion
S52559M	Other extraarticular fracture of lower end of unspecified radius, subsequent encounter for open fracture type I or II with nonunion
S52559N	Other extraarticular fracture of lower end of unspecified radius, subsequent encounter for open fracture type IIIA, IIIB, or IIIC with nonunion
S52561K	Bartons fracture of right radius, subsequent encounter for closed fracture with nonunion
S52561M	Bartons fracture of right radius, subsequent encounter for open fracture type I or II with nonunion
S52561N	Bartons fracture of right radius, subsequent encounter for open fracture type IIIA, IIIB, or IIIC with nonunion
S52562K	Bartons fracture of left radius, subsequent encounter for closed fracture with nonunion
S52562M	Bartons fracture of left radius, subsequent encounter for open fracture type I or II with nonunion
S52562N	Bartons fracture of left radius, subsequent encounter for open fracture type IIIA, IIIB, or IIIC with nonunion
S52569K	Bartons fracture of unspecified radius, subsequent encounter for closed fracture with nonunion
S52569M	Bartons fracture of unspecified radius, subsequent encounter for open fracture type I or II with nonunion
S52569N	Bartons fracture of unspecified radius, subsequent encounter for open fracture type IIIA, IIIB, or IIIC with nonunion
S52571K	Other intraarticular fracture of lower end of right radius, subsequent encounter for closed fracture with nonunion
S52571M	Other intraarticular fracture of lower end of right radius, subsequent encounter for open fracture type I or II with nonunion
S52571N	Other intraarticular fracture of lower end of right radius, subsequent encounter for open fracture type IIIA, IIIB, or IIIC with nonunion
S52572K	Other intraarticular fracture of lower end of left radius, subsequent encounter for closed fracture with nonunion

S52572M	Other intraarticular fracture of lower end of left radius, subsequent encounter for open fracture type I or II with nonunion
S52572N	Other intraarticular fracture of lower end of left radius, subsequent encounter for open fracture type IIIA, IIIB, or IIIC with nonunion
S52579K	Other intraarticular fracture of lower end of unspecified radius, subsequent encounter for closed fracture with nonunion
S52579M	Other intraarticular fracture of lower end of unspecified radius, subsequent encounter for open fracture type I or II with nonunion
S52579N	Other intraarticular fracture of lower end of unspecified radius, subsequent encounter for open fracture type IIIA, IIIB, or IIIC with nonunion
S52591K	Other fractures of lower end of right radius, subsequent encounter for closed fracture with nonunion
S52591M	Other fractures of lower end of right radius, subsequent encounter for open fracture type I or II with nonunion
S52591N	Other fractures of lower end of right radius, subsequent encounter for open fracture type IIIA, IIIB, or IIIC with nonunion
S52592K	Other fractures of lower end of left radius, subsequent encounter for closed fracture with nonunion
S52592M	Other fractures of lower end of left radius, subsequent encounter for open fracture type I or II with nonunion
S52592N	Other fractures of lower end of left radius, subsequent encounter for open fracture type IIIA, IIIB, or IIIC with nonunion
S52599K	Other fractures of lower end of unspecified radius, subsequent encounter for closed fracture with nonunion
S52599M	Other fractures of lower end of unspecified radius, subsequent encounter for open fracture type I or II with nonunion
S52599N	Other fractures of lower end of unspecified radius, subsequent encounter for open fracture type IIIA, IIIB, or IIIC with nonunion
S52601K	Unspecified fracture of lower end of right ulna, subsequent encounter for closed fracture with nonunion
S52601M	Unspecified fracture of lower end of right ulna, subsequent encounter for open fracture type I or II with nonunion

S52601N	Unspecified fracture of lower end of right ulna, subsequent encounter for open fracture type IIIA, IIIB, or IIIC with nonunion
S52602K	Unspecified fracture of lower end of left ulna, subsequent encounter for closed fracture with nonunion
S52602M	Unspecified fracture of lower end of left ulna, subsequent encounter for open fracture type I or II with nonunion
S52602N	Unspecified fracture of lower end of left ulna, subsequent encounter for open fracture type IIIA, IIIB, or IIIC with nonunion
S52609K	Unspecified fracture of lower end of unspecified ulna, subsequent encounter for closed fracture with nonunion
S52609M	Unspecified fracture of lower end of unspecified ulna, subsequent encounter for open fracture type I or II with nonunion
S52609N	Unspecified fracture of lower end of unspecified ulna, subsequent encounter for open fracture type IIIA, IIIB, or IIIC with nonunion
S52611K	Displaced fracture of right ulna styloid process, subsequent encounter for closed fracture with nonunion
S52611M	Displaced fracture of right ulna styloid process, subsequent encounter for open fracture type I or II with nonunion
S52611N	Displaced fracture of right ulna styloid process, subsequent encounter for open fracture type IIIA, IIIB, or IIIC with nonunion
S52612K	Displaced fracture of left ulna styloid process, subsequent encounter for closed fracture with nonunion
S52612M	Displaced fracture of left ulna styloid process, subsequent encounter for open fracture type I or II with nonunion
S52612N	Displaced fracture of left ulna styloid process, subsequent encounter for open fracture type IIIA, IIIB, or IIIC with nonunion
S52613K	Displaced fracture of unspecified ulna styloid process, subsequent encounter for closed fracture with nonunion
S52613M	Displaced fracture of unspecified ulna styloid process, subsequent encounter for open fracture type I or II with nonunion
S52613N	Displaced fracture of unspecified ulna styloid process, subsequent encounter for open fracture type IIIA, IIIB, or IIIC with nonunion

S52614K	Nondisplaced fracture of right ulna styloid process, subsequent encounter for closed fracture with nonunion
S52614M	Nondisplaced fracture of right ulna styloid process, subsequent encounter for open fracture type I or II with nonunion
S52614N	Nondisplaced fracture of right ulna styloid process, subsequent encounter for open fracture type IIIA, IIIB, or IIIC with nonunion
S52615K	Nondisplaced fracture of left ulna styloid process, subsequent encounter for closed fracture with nonunion
S52615M	Nondisplaced fracture of left ulna styloid process, subsequent encounter for open fracture type I or II with nonunion
S52615N	Nondisplaced fracture of left ulna styloid process, subsequent encounter for open fracture type IIIA, IIIB, or IIIC with nonunion
S52616K	Nondisplaced fracture of unspecified ulna styloid process, subsequent encounter for closed fracture with nonunion
S52616M	Nondisplaced fracture of unspecified ulna styloid process, subsequent encounter for open fracture type I or II with nonunion
S52616N	Nondisplaced fracture of unspecified ulna styloid process, subsequent encounter for open fracture type IIIA, IIIB, or IIIC with nonunion
S52621K	Torus fracture of lower end of right ulna, subsequent encounter for fracture with nonunion
S52622K	Torus fracture of lower end of left ulna, subsequent encounter for fracture with nonunion
S52629K	Torus fracture of lower end of unspecified ulna, subsequent encounter for fracture with nonunion
S52691K	Other fracture of lower end of right ulna, subsequent encounter for closed fracture with nonunion
S52691M	Other fracture of lower end of right ulna, subsequent encounter for open fracture type I or II with nonunion
S52691N	Other fracture of lower end of right ulna, subsequent encounter for open fracture type IIIA, IIIB, or IIIC with nonunion
S52692K	Other fracture of lower end of left ulna, subsequent encounter for closed fracture with nonunion

S52692M	Other fracture of lower end of left ulna, subsequent encounter for open fracture type I or II with nonunion
S52692N	Other fracture of lower end of left ulna, subsequent encounter for open fracture type IIIA, IIIB, or IIIC with nonunion
S52699K	Other fracture of lower end of unspecified ulna, subsequent encounter for closed fracture with nonunion
S52699M	Other fracture of lower end of unspecified ulna, subsequent encounter for open fracture type I or II with nonunion
S52699N	Other fracture of lower end of unspecified ulna, subsequent encounter for open fracture type IIIA, IIIB, or IIIC with nonunion
S5290XK	Unspecified fracture of unspecified forearm, subsequent encounter for closed fracture with nonunion
S5290XM	Unspecified fracture of unspecified forearm, subsequent encounter for open fracture type I or II with nonunion
S5290XN	Unspecified fracture of unspecified forearm, subsequent encounter for open fracture type IIIA, IIIB, or IIIC with nonunion
S5291XK	Unspecified fracture of right forearm, subsequent encounter for closed fracture with nonunion
S5291XM	Unspecified fracture of right forearm, subsequent encounter for open fracture type I or II with nonunion
S5291XN	Unspecified fracture of right forearm, subsequent encounter for open fracture type IIIA, IIIB, or IIIC with nonunion
S5292XK	Unspecified fracture of left forearm, subsequent encounter for closed fracture with nonunion
S5292XM	Unspecified fracture of left forearm, subsequent encounter for open fracture type I or II with nonunion
S5292XN	Unspecified fracture of left forearm, subsequent encounter for open fracture type IIIA, IIIB, or IIIC with nonunion
S59001K	Unspecified physeal fracture of lower end of ulna, right arm, subsequent encounter for fracture with nonunion
S59002K	Unspecified physeal fracture of lower end of ulna, left arm, subsequent encounter for fracture with nonunion

S59009K	Unspecified physeal fracture of lower end of ulna, unspecified arm, subsequent encounter for fracture with nonunion
S59011K	Salter-Harris Type I physeal fracture of lower end of ulna, right arm, subsequent encounter for fracture with nonunion
S59012K	Salter-Harris Type I physeal fracture of lower end of ulna, left arm, subsequent encounter for fracture with nonunion
S59019K	Salter-Harris Type I physeal fracture of lower end of ulna, unspecified arm, subsequent encounter for fracture with nonunion
S59021K	Salter-Harris Type II physeal fracture of lower end of ulna, right arm, subsequent encounter for fracture with nonunion
S59022K	Salter-Harris Type II physeal fracture of lower end of ulna, left arm, subsequent encounter for fracture with nonunion
S59029K	Salter-Harris Type II physeal fracture of lower end of ulna, unspecified arm, subsequent encounter for fracture with nonunion
S59031K	Salter-Harris Type III physeal fracture of lower end of ulna, right arm, subsequent encounter for fracture with nonunion
S59032K	Salter-Harris Type III physeal fracture of lower end of ulna, left arm, subsequent encounter for fracture with nonunion
S59039K	Salter-Harris Type III physeal fracture of lower end of ulna, unspecified arm, subsequent encounter for fracture with nonunion
S59041K	Salter-Harris Type IV physeal fracture of lower end of ulna, right arm, subsequent encounter for fracture with nonunion
S59042K	Salter-Harris Type IV physeal fracture of lower end of ulna, left arm, subsequent encounter for fracture with nonunion
S59049K	Salter-Harris Type IV physeal fracture of lower end of ulna, unspecified arm, subsequent encounter for fracture with nonunion
S59091K	Other physeal fracture of lower end of ulna, right arm, subsequent encounter for fracture with nonunion
S59092K	Other physeal fracture of lower end of ulna, left arm, subsequent encounter for fracture with nonunion
S59099K	Other physeal fracture of lower end of ulna, unspecified arm, subsequent encounter for fracture with nonunion

S59101K	Unspecified physeal fracture of upper end of radius, right arm, subsequent encounter for fracture with nonunion
S59102K	Unspecified physeal fracture of upper end of radius, left arm, subsequent encounter for fracture with nonunion
S59109K	Unspecified physeal fracture of upper end of radius, unspecified arm, subsequent encounter for fracture with nonunion
S59111K	Salter-Harris Type I physeal fracture of upper end of radius, right arm, subsequent encounter for fracture with nonunion
S59112K	Salter-Harris Type I physeal fracture of upper end of radius, left arm, subsequent encounter for fracture with nonunion
S59119K	Salter-Harris Type I physeal fracture of upper end of radius, unspecified arm, subsequent encounter for fracture with nonunion
S59121K	Salter-Harris Type II physeal fracture of upper end of radius, right arm, subsequent encounter for fracture with nonunion
S59122K	Salter-Harris Type II physeal fracture of upper end of radius, left arm, subsequent encounter for fracture with nonunion
S59129K	Salter-Harris Type II physeal fracture of upper end of radius, unspecified arm, subsequent encounter for fracture with nonunion
S59131K	Salter-Harris Type III physeal fracture of upper end of radius, right arm, subsequent encounter for fracture with nonunion
S59132K	Salter-Harris Type III physeal fracture of upper end of radius, left arm, subsequent encounter for fracture with nonunion
S59139K	Salter-Harris Type III physeal fracture of upper end of radius, unspecified arm, subsequent encounter for fracture with nonunion
S59141K	Salter-Harris Type IV physeal fracture of upper end of radius, right arm, subsequent encounter for fracture with nonunion
S59142K	Salter-Harris Type IV physeal fracture of upper end of radius, left arm, subsequent encounter for fracture with nonunion
S59149K	Salter-Harris Type IV physeal fracture of upper end of radius, unspecified arm, subsequent encounter for fracture with nonunion
S59191K	Other physeal fracture of upper end of radius, right arm, subsequent encounter for fracture with nonunion

S59192K	Other physeal fracture of upper end of radius, left arm, subsequent encounter for fracture with nonunion
S59199K	Other physeal fracture of upper end of radius, unspecified arm, subsequent encounter for fracture with nonunion
S59201K	Unspecified physeal fracture of lower end of radius, right arm, subsequent encounter for fracture with nonunion
S59202K	Unspecified physeal fracture of lower end of radius, left arm, subsequent encounter for fracture with nonunion
S59209K	Unspecified physeal fracture of lower end of radius, unspecified arm, subsequent encounter for fracture with nonunion
S59211K	Salter-Harris Type I physeal fracture of lower end of radius, right arm, subsequent encounter for fracture with nonunion
S59212K	Salter-Harris Type I physeal fracture of lower end of radius, left arm, subsequent encounter for fracture with nonunion
S59219K	Salter-Harris Type I physeal fracture of lower end of radius, unspecified arm, subsequent encounter for fracture with nonunion
S59221K	Salter-Harris Type II physeal fracture of lower end of radius, right arm, subsequent encounter for fracture with nonunion
S59222K	Salter-Harris Type II physeal fracture of lower end of radius, left arm, subsequent encounter for fracture with nonunion
S59229K	Salter-Harris Type II physeal fracture of lower end of radius, unspecified arm, subsequent encounter for fracture with nonunion
S59231K	Salter-Harris Type III physeal fracture of lower end of radius, right arm, subsequent encounter for fracture with nonunion
S59232K	Salter-Harris Type III physeal fracture of lower end of radius, left arm, subsequent encounter for fracture with nonunion
S59239K	Salter-Harris Type III physeal fracture of lower end of radius, unspecified arm, subsequent encounter for fracture with nonunion
S59241K	Salter-Harris Type IV physeal fracture of lower end of radius, right arm, subsequent encounter for fracture with nonunion
S59242K	Salter-Harris Type IV physeal fracture of lower end of radius, left arm, subsequent encounter for fracture with nonunion

S59249K	Salter-Harris Type IV physeal fracture of lower end of radius, unspecified arm, subsequent encounter for fracture with nonunion
S59291K	Other physeal fracture of lower end of radius, right arm, subsequent encounter for fracture with nonunion
S59292K	Other physeal fracture of lower end of radius, left arm, subsequent encounter for fracture with nonunion
S59299K	Other physeal fracture of lower end of radius, unspecified arm, subsequent encounter for fracture with nonunion
S62001K	Unspecified fracture of navicular [scaphoid] bone of right wrist, subsequent encounter for fracture with nonunion
S62002K	Unspecified fracture of navicular [scaphoid] bone of left wrist, subsequent encounter for fracture with nonunion
S62009K	Unspecified fracture of navicular [scaphoid] bone of unspecified wrist, subsequent encounter for fracture with nonunion
S62011K	Displaced fracture of distal pole of navicular [scaphoid] bone of right wrist, subsequent encounter for fracture with nonunion
S62012K	Displaced fracture of distal pole of navicular [scaphoid] bone of left wrist, subsequent encounter for fracture with nonunion
S62013K	Displaced fracture of distal pole of navicular [scaphoid] bone of unspecified wrist, subsequent encounter for fracture with nonunion
S62014K	Nondisplaced fracture of distal pole of navicular [scaphoid] bone of right wrist, subsequent encounter for fracture with nonunion
S62015K	Nondisplaced fracture of distal pole of navicular [scaphoid] bone of left wrist, subsequent encounter for fracture with nonunion
S62016K	Nondisplaced fracture of distal pole of navicular [scaphoid] bone of unspecified wrist, subsequent encounter for fracture with nonunion
S62021K	Displaced fracture of middle third of navicular [scaphoid] bone of right wrist, subsequent encounter for fracture with nonunion
S62022K	Displaced fracture of middle third of navicular [scaphoid] bone of left wrist, subsequent encounter for fracture with nonunion
S62023K	Displaced fracture of middle third of navicular [scaphoid] bone of unspecified wrist, subsequent encounter for fracture with nonunion

S62024K	Nondisplaced fracture of middle third of navicular [scaphoid] bone of right wrist, subsequent encounter for fracture with nonunion
S62025K	Nondisplaced fracture of middle third of navicular [scaphoid] bone of left wrist, subsequent encounter for fracture with nonunion
S62026K	Nondisplaced fracture of middle third of navicular [scaphoid] bone of unspecified wrist, subsequent encounter for fracture with nonunion
S62031K	Displaced fracture of proximal third of navicular [scaphoid] bone of right wrist, subsequent encounter for fracture with nonunion
S62032K	Displaced fracture of proximal third of navicular [scaphoid] bone of left wrist, subsequent encounter for fracture with nonunion
S62033K	Displaced fracture of proximal third of navicular [scaphoid] bone of unspecified wrist, subsequent encounter for fracture with nonunion
S62034K	Nondisplaced fracture of proximal third of navicular [scaphoid] bone of right wrist, subsequent encounter for fracture with nonunion
S62035K	Nondisplaced fracture of proximal third of navicular [scaphoid] bone of left wrist, subsequent encounter for fracture with nonunion
S62036K	Nondisplaced fracture of proximal third of navicular [scaphoid] bone of unspecified wrist, subsequent encounter for fracture with nonunion
S62101K	Fracture of unspecified carpal bone, right wrist, subsequent encounter for fracture with nonunion
S62102K	Fracture of unspecified carpal bone, left wrist, subsequent encounter for fracture with nonunion
S62109K	Fracture of unspecified carpal bone, unspecified wrist, subsequent encounter for fracture with nonunion
S62111K	Displaced fracture of triquetrum [cuneiform] bone, right wrist, subsequent encounter for fracture with nonunion
S62112K	Displaced fracture of triquetrum [cuneiform] bone, left wrist, subsequent encounter for fracture with nonunion
S62113K	Displaced fracture of triquetrum [cuneiform] bone, unspecified wrist, subsequent encounter for fracture with nonunion
S62114K	Nondisplaced fracture of triquetrum [cuneiform] bone, right wrist, subsequent encounter for fracture with nonunion

S62115K	Nondisplaced fracture of triquetrum [cuneiform] bone, left wrist, subsequent encounter for fracture with nonunion
S62116K	Nondisplaced fracture of triquetrum [cuneiform] bone, unspecified wrist, subsequent encounter for fracture with nonunion
S62121K	Displaced fracture of lunate [semilunar], right wrist, subsequent encounter for fracture with nonunion
S62122K	Displaced fracture of lunate [semilunar], left wrist, subsequent encounter for fracture with nonunion
S62123K	Displaced fracture of lunate [semilunar], unspecified wrist, subsequent encounter for fracture with nonunion
S62124K	Nondisplaced fracture of lunate [semilunar], right wrist, subsequent encounter for fracture with nonunion
S62125K	Nondisplaced fracture of lunate [semilunar], left wrist, subsequent encounter for fracture with nonunion
S62126K	Nondisplaced fracture of lunate [semilunar], unspecified wrist, subsequent encounter for fracture with nonunion
S62131K	Displaced fracture of capitate [os magnum] bone, right wrist, subsequent encounter for fracture with nonunion
S62132K	Displaced fracture of capitate [os magnum] bone, left wrist, subsequent encounter for fracture with nonunion
S62133K	Displaced fracture of capitate [os magnum] bone, unspecified wrist, subsequent encounter for fracture with nonunion
S62134K	Nondisplaced fracture of capitate [os magnum] bone, right wrist, subsequent encounter for fracture with nonunion
S62135K	Nondisplaced fracture of capitate [os magnum] bone, left wrist, subsequent encounter for fracture with nonunion
S62136K	Nondisplaced fracture of capitate [os magnum] bone, unspecified wrist, subsequent encounter for fracture with nonunion
S62141K	Displaced fracture of body of hamate [unciform] bone, right wrist, subsequent encounter for fracture with nonunion
S62142K	Displaced fracture of body of hamate [unciform] bone, left wrist, subsequent encounter for fracture with nonunion

S62143K	Displaced fracture of body of hamate [unciform] bone, unspecified wrist, subsequent encounter for fracture with nonunion
S62144K	Nondisplaced fracture of body of hamate [unciform] bone, right wrist, subsequent encounter for fracture with nonunion
S62145K	Nondisplaced fracture of body of hamate [unciform] bone, left wrist, subsequent encounter for fracture with nonunion
S62146K	Nondisplaced fracture of body of hamate [unciform] bone, unspecified wrist, subsequent encounter for fracture with nonunion
S62151K	Displaced fracture of hook process of hamate [unciform] bone, right wrist, subsequent encounter for fracture with nonunion
S62152K	Displaced fracture of hook process of hamate [unciform] bone, left wrist, subsequent encounter for fracture with nonunion
S62153K	Displaced fracture of hook process of hamate [unciform] bone, unspecified wrist, subsequent encounter for fracture with nonunion
S62154K	Nondisplaced fracture of hook process of hamate [unciform] bone, right wrist, subsequent encounter for fracture with nonunion
S62155K	Nondisplaced fracture of hook process of hamate [unciform] bone, left wrist, subsequent encounter for fracture with nonunion
S62156K	Nondisplaced fracture of hook process of hamate [unciform] bone, unspecified wrist, subsequent encounter for fracture with nonunion
S62161K	Displaced fracture of pisiform, right wrist, subsequent encounter for fracture with nonunion
S62162K	Displaced fracture of pisiform, left wrist, subsequent encounter for fracture with nonunion
S62163K	Displaced fracture of pisiform, unspecified wrist, subsequent encounter for fracture with nonunion
S62164K	Nondisplaced fracture of pisiform, right wrist, subsequent encounter for fracture with nonunion
S62165K	Nondisplaced fracture of pisiform, left wrist, subsequent encounter for fracture with nonunion
S62166K	Nondisplaced fracture of pisiform, unspecified wrist, subsequent encounter for fracture with nonunion

S62171K	Displaced fracture of trapezium [larger multangular], right wrist, subsequent encounter for fracture with nonunion
S62172K	Displaced fracture of trapezium [larger multangular], left wrist, subsequent encounter for fracture with nonunion
S62173K	Displaced fracture of trapezium [larger multangular], unspecified wrist, subsequent encounter for fracture with nonunion
S62174K	Nondisplaced fracture of trapezium [larger multangular], right wrist, subsequent encounter for fracture with nonunion
S62175K	Nondisplaced fracture of trapezium [larger multangular], left wrist, subsequent encounter for fracture with nonunion
S62176K	Nondisplaced fracture of trapezium [larger multangular], unspecified wrist, subsequent encounter for fracture with nonunion
S62181K	Displaced fracture of trapezoid [smaller multangular], right wrist, subsequent encounter for fracture with nonunion
S62182K	Displaced fracture of trapezoid [smaller multangular], left wrist, subsequent encounter for fracture with nonunion
S62183K	Displaced fracture of trapezoid [smaller multangular], unspecified wrist, subsequent encounter for fracture with nonunion
S62184K	Nondisplaced fracture of trapezoid [smaller multangular], right wrist, subsequent encounter for fracture with nonunion
S62185K	Nondisplaced fracture of trapezoid [smaller multangular], left wrist, subsequent encounter for fracture with nonunion
S62186K	Nondisplaced fracture of trapezoid [smaller multangular], unspecified wrist, subsequent encounter for fracture with nonunion
S62201K	Unspecified fracture of first metacarpal bone, right hand, subsequent encounter for fracture with nonunion
S62202K	Unspecified fracture of first metacarpal bone, left hand, subsequent encounter for fracture with nonunion
S62209K	Unspecified fracture of first metacarpal bone, unspecified hand, subsequent encounter for fracture with nonunion
S62211K	Bennetts fracture, right hand, subsequent encounter for fracture with nonunion
S62212K	Bennetts fracture, left hand, subsequent encounter for fracture with nonunion

S62213K	Bennetts fracture, unspecified hand, subsequent encounter for fracture with nonunion
S62221K	Displaced Rolandos fracture, right hand, subsequent encounter for fracture with nonunion
S62222K	Displaced Rolandos fracture, left hand, subsequent encounter for fracture with nonunion
S62223K	Displaced Rolandos fracture, unspecified hand, subsequent encounter for fracture with nonunion
S62224K	Nondisplaced Rolandos fracture, right hand, subsequent encounter for fracture with nonunion
S62225K	Nondisplaced Rolandos fracture, left hand, subsequent encounter for fracture with nonunion
S62226K	Nondisplaced Rolandos fracture, unspecified hand, subsequent encounter for fracture with nonunion
S62231K	Other displaced fracture of base of first metacarpal bone, right hand, subsequent encounter for fracture with nonunion
S62232K	Other displaced fracture of base of first metacarpal bone, left hand, subsequent encounter for fracture with nonunion
S62233K	Other displaced fracture of base of first metacarpal bone, unspecified hand, subsequent encounter for fracture with nonunion
S62234K	Other nondisplaced fracture of base of first metacarpal bone, right hand, subsequent encounter for fracture with nonunion
S62235K	Other nondisplaced fracture of base of first metacarpal bone, left hand, subsequent encounter for fracture with nonunion
S62236K	Other nondisplaced fracture of base of first metacarpal bone, unspecified hand, subsequent encounter for fracture with nonunion
S62241K	Displaced fracture of shaft of first metacarpal bone, right hand, subsequent encounter for fracture with nonunion
S62242K	Displaced fracture of shaft of first metacarpal bone, left hand, subsequent encounter for fracture with nonunion
S62243K	Displaced fracture of shaft of first metacarpal bone, unspecified hand, subsequent encounter for fracture with nonunion

S62244K	Nondisplaced fracture of shaft of first metacarpal bone, right hand, subsequent encounter for fracture with nonunion
S62245K	Nondisplaced fracture of shaft of first metacarpal bone, left hand, subsequent encounter for fracture with nonunion
S62246K	Nondisplaced fracture of shaft of first metacarpal bone, unspecified hand, subsequent encounter for fracture with nonunion
S62251K	Displaced fracture of neck of first metacarpal bone, right hand, subsequent encounter for fracture with nonunion
S62252K	Displaced fracture of neck of first metacarpal bone, left hand, subsequent encounter for fracture with nonunion
S62253K	Displaced fracture of neck of first metacarpal bone, unspecified hand, subsequent encounter for fracture with nonunion
S62254K	Nondisplaced fracture of neck of first metacarpal bone, right hand, subsequent encounter for fracture with nonunion
S62255K	Nondisplaced fracture of neck of first metacarpal bone, left hand, subsequent encounter for fracture with nonunion
S62256K	Nondisplaced fracture of neck of first metacarpal bone, unspecified hand, subsequent encounter for fracture with nonunion
S62291K	Other fracture of first metacarpal bone, right hand, subsequent encounter for fracture with nonunion
S62292K	Other fracture of first metacarpal bone, left hand, subsequent encounter for fracture with nonunion
S62299K	Other fracture of first metacarpal bone, unspecified hand, subsequent encounter for fracture with nonunion
S62300K	Unspecified fracture of second metacarpal bone, right hand, subsequent encounter for fracture with nonunion
S62301K	Unspecified fracture of second metacarpal bone, left hand, subsequent encounter for fracture with nonunion
S62302K	Unspecified fracture of third metacarpal bone, right hand, subsequent encounter for fracture with nonunion
S62303K	Unspecified fracture of third metacarpal bone, left hand, subsequent encounter for fracture with nonunion

S62304K	Unspecified fracture of fourth metacarpal bone, right hand, subsequent encounter for fracture with nonunion
S62305K	Unspecified fracture of fourth metacarpal bone, left hand, subsequent encounter for fracture with nonunion
S62306K	Unspecified fracture of fifth metacarpal bone, right hand, subsequent encounter for fracture with nonunion
S62307K	Unspecified fracture of fifth metacarpal bone, left hand, subsequent encounter for fracture with nonunion
S62308K	Unspecified fracture of other metacarpal bone, subsequent encounter for fracture with nonunion
S62309K	Unspecified fracture of unspecified metacarpal bone, subsequent encounter for fracture with nonunion
S62310K	Displaced fracture of base of second metacarpal bone, right hand, subsequent encounter for fracture with nonunion
S62311K	Displaced fracture of base of second metacarpal bone, left hand, subsequent encounter for fracture with nonunion
S62312K	Displaced fracture of base of third metacarpal bone, right hand, subsequent encounter for fracture with nonunion
S62313K	Displaced fracture of base of third metacarpal bone, left hand, subsequent encounter for fracture with nonunion
S62314K	Displaced fracture of base of fourth metacarpal bone, right hand, subsequent encounter for fracture with nonunion
S62315K	Displaced fracture of base of fourth metacarpal bone, left hand, subsequent encounter for fracture with nonunion
S62316K	Displaced fracture of base of fifth metacarpal bone, right hand, subsequent encounter for fracture with nonunion
S62317K	Displaced fracture of base of fifth metacarpal bone, left hand, subsequent encounter for fracture with nonunion
S62318K	Displaced fracture of base of other metacarpal bone, subsequent encounter for fracture with nonunion
S62319K	Displaced fracture of base of unspecified metacarpal bone, subsequent encounter for fracture with nonunion

S62320K	Displaced fracture of shaft of second metacarpal bone, right hand, subsequent encounter for fracture with nonunion
S62321K	Displaced fracture of shaft of second metacarpal bone, left hand, subsequent encounter for fracture with nonunion
S62322K	Displaced fracture of shaft of third metacarpal bone, right hand, subsequent encounter for fracture with nonunion
S62323K	Displaced fracture of shaft of third metacarpal bone, left hand, subsequent encounter for fracture with nonunion
S62324K	Displaced fracture of shaft of fourth metacarpal bone, right hand, subsequent encounter for fracture with nonunion
S62325K	Displaced fracture of shaft of fourth metacarpal bone, left hand, subsequent encounter for fracture with nonunion
S62326K	Displaced fracture of shaft of fifth metacarpal bone, right hand, subsequent encounter for fracture with nonunion
S62327K	Displaced fracture of shaft of fifth metacarpal bone, left hand, subsequent encounter for fracture with nonunion
S62328K	Displaced fracture of shaft of other metacarpal bone, subsequent encounter for fracture with nonunion
S62329K	Displaced fracture of shaft of unspecified metacarpal bone, subsequent encounter for fracture with nonunion
S62330K	Displaced fracture of neck of second metacarpal bone, right hand, subsequent encounter for fracture with nonunion
S62331K	Displaced fracture of neck of second metacarpal bone, left hand, subsequent encounter for fracture with nonunion
S62332K	Displaced fracture of neck of third metacarpal bone, right hand, subsequent encounter for fracture with nonunion
S62333K	Displaced fracture of neck of third metacarpal bone, left hand, subsequent encounter for fracture with nonunion
S62334K	Displaced fracture of neck of fourth metacarpal bone, right hand, subsequent encounter for fracture with nonunion
S62335K	Displaced fracture of neck of fourth metacarpal bone, left hand, subsequent encounter for fracture with nonunion

S62336K	Displaced fracture of neck of fifth metacarpal bone, right hand, subsequent encounter for fracture with nonunion
S62337K	Displaced fracture of neck of fifth metacarpal bone, left hand, subsequent encounter for fracture with nonunion
S62338K	Displaced fracture of neck of other metacarpal bone, subsequent encounter for fracture with nonunion
S62339K	Displaced fracture of neck of unspecified metacarpal bone, subsequent encounter for fracture with nonunion
S62340K	Nondisplaced fracture of base of second metacarpal bone, right hand, subsequent encounter for fracture with nonunion
S62341K	Nondisplaced fracture of base of second metacarpal bone, left hand, subsequent encounter for fracture with nonunion
S62342K	Nondisplaced fracture of base of third metacarpal bone, right hand, subsequent encounter for fracture with nonunion
S62343K	Nondisplaced fracture of base of third metacarpal bone, left hand, subsequent encounter for fracture with nonunion
S62344K	Nondisplaced fracture of base of fourth metacarpal bone, right hand, subsequent encounter for fracture with nonunion
S62345K	Nondisplaced fracture of base of fourth metacarpal bone, left hand, subsequent encounter for fracture with nonunion
S62346K	Nondisplaced fracture of base of fifth metacarpal bone, right hand, subsequent encounter for fracture with nonunion
S62347K	Nondisplaced fracture of base of fifth metacarpal bone, left hand, subsequent encounter for fracture with nonunion
S62348K	Nondisplaced fracture of base of other metacarpal bone, subsequent encounter for fracture with nonunion
S62349K	Nondisplaced fracture of base of unspecified metacarpal bone, subsequent encounter for fracture with nonunion
S62350K	Nondisplaced fracture of shaft of second metacarpal bone, right hand, subsequent encounter for fracture with nonunion
S62351K	Nondisplaced fracture of shaft of second metacarpal bone, left hand, subsequent encounter for fracture with nonunion

S62352K	Nondisplaced fracture of shaft of third metacarpal bone, right hand, subsequent encounter for fracture with nonunion
S62353K	Nondisplaced fracture of shaft of third metacarpal bone, left hand, subsequent encounter for fracture with nonunion
S62354K	Nondisplaced fracture of shaft of fourth metacarpal bone, right hand, subsequent encounter for fracture with nonunion
S62355K	Nondisplaced fracture of shaft of fourth metacarpal bone, left hand, subsequent encounter for fracture with nonunion
S62356K	Nondisplaced fracture of shaft of fifth metacarpal bone, right hand, subsequent encounter for fracture with nonunion
S62357K	Nondisplaced fracture of shaft of fifth metacarpal bone, left hand, subsequent encounter for fracture with nonunion
S62358K	Nondisplaced fracture of shaft of other metacarpal bone, subsequent encounter for fracture with nonunion
S62359K	Nondisplaced fracture of shaft of unspecified metacarpal bone, subsequent encounter for fracture with nonunion
S62360K	Nondisplaced fracture of neck of second metacarpal bone, right hand, subsequent encounter for fracture with nonunion
S62361K	Nondisplaced fracture of neck of second metacarpal bone, left hand, subsequent encounter for fracture with nonunion
S62362K	Nondisplaced fracture of neck of third metacarpal bone, right hand, subsequent encounter for fracture with nonunion
S62363K	Nondisplaced fracture of neck of third metacarpal bone, left hand, subsequent encounter for fracture with nonunion
S62364K	Nondisplaced fracture of neck of fourth metacarpal bone, right hand, subsequent encounter for fracture with nonunion
S62365K	Nondisplaced fracture of neck of fourth metacarpal bone, left hand, subsequent encounter for fracture with nonunion
S62366K	Nondisplaced fracture of neck of fifth metacarpal bone, right hand, subsequent encounter for fracture with nonunion
S62367K	Nondisplaced fracture of neck of fifth metacarpal bone, left hand, subsequent encounter for fracture with nonunion

S62368K	Nondisplaced fracture of neck of other metacarpal bone, subsequent encounter for fracture with nonunion
S62369K	Nondisplaced fracture of neck of unspecified metacarpal bone, subsequent encounter for fracture with nonunion
S62390K	Other fracture of second metacarpal bone, right hand, subsequent encounter for fracture with nonunion
S62391K	Other fracture of second metacarpal bone, left hand, subsequent encounter for fracture with nonunion
S62392K	Other fracture of third metacarpal bone, right hand, subsequent encounter for fracture with nonunion
S62393K	Other fracture of third metacarpal bone, left hand, subsequent encounter for fracture with nonunion
S62394K	Other fracture of fourth metacarpal bone, right hand, subsequent encounter for fracture with nonunion
S62395K	Other fracture of fourth metacarpal bone, left hand, subsequent encounter for fracture with nonunion
S62396K	Other fracture of fifth metacarpal bone, right hand, subsequent encounter for fracture with nonunion
S62397K	Other fracture of fifth metacarpal bone, left hand, subsequent encounter for fracture with nonunion
S62398K	Other fracture of other metacarpal bone, subsequent encounter for fracture with nonunion
S62399K	Other fracture of unspecified metacarpal bone, subsequent encounter for fracture with nonunion
S62501K	Fracture of unspecified phalanx of right thumb, subsequent encounter for fracture with nonunion
S62502K	Fracture of unspecified phalanx of left thumb, subsequent encounter for fracture with nonunion
S62509K	Fracture of unspecified phalanx of unspecified thumb, subsequent encounter for fracture with nonunion
S62511K	Displaced fracture of proximal phalanx of right thumb, subsequent encounter for fracture with nonunion

S62512K	Displaced fracture of proximal phalanx of left thumb, subsequent encounter for fracture with nonunion
S62513K	Displaced fracture of proximal phalanx of unspecified thumb, subsequent encounter for fracture with nonunion
S62514K	Nondisplaced fracture of proximal phalanx of right thumb, subsequent encounter for fracture with nonunion
S62515K	Nondisplaced fracture of proximal phalanx of left thumb, subsequent encounter for fracture with nonunion
S62516K	Nondisplaced fracture of proximal phalanx of unspecified thumb, subsequent encounter for fracture with nonunion
S62521K	Displaced fracture of distal phalanx of right thumb, subsequent encounter for fracture with nonunion
S62522K	Displaced fracture of distal phalanx of left thumb, subsequent encounter for fracture with nonunion
S62523K	Displaced fracture of distal phalanx of unspecified thumb, subsequent encounter for fracture with nonunion
S62524K	Nondisplaced fracture of distal phalanx of right thumb, subsequent encounter for fracture with nonunion
S62525K	Nondisplaced fracture of distal phalanx of left thumb, subsequent encounter for fracture with nonunion
S62526K	Nondisplaced fracture of distal phalanx of unspecified thumb, subsequent encounter for fracture with nonunion
S62600K	Fracture of unspecified phalanx of right index finger, subsequent encounter for fracture with nonunion
S62601K	Fracture of unspecified phalanx of left index finger, subsequent encounter for fracture with nonunion
S62602K	Fracture of unspecified phalanx of right middle finger, subsequent encounter for fracture with nonunion
S62603K	Fracture of unspecified phalanx of left middle finger, subsequent encounter for fracture with nonunion
S62604K	Fracture of unspecified phalanx of right ring finger, subsequent encounter for fracture with nonunion

S62605K	Fracture of unspecified phalanx of left ring finger, subsequent encounter for fracture with nonunion
S62606K	Fracture of unspecified phalanx of right little finger, subsequent encounter for fracture with nonunion
S62607K	Fracture of unspecified phalanx of left little finger, subsequent encounter for fracture with nonunion
S62608K	Fracture of unspecified phalanx of other finger, subsequent encounter for fracture with nonunion
S62609K	Fracture of unspecified phalanx of unspecified finger, subsequent encounter for fracture with nonunion
S62610K	Displaced fracture of proximal phalanx of right index finger, subsequent encounter for fracture with nonunion
S62611K	Displaced fracture of proximal phalanx of left index finger, subsequent encounter for fracture with nonunion
S62612K	Displaced fracture of proximal phalanx of right middle finger, subsequent encounter for fracture with nonunion
S62613K	Displaced fracture of proximal phalanx of left middle finger, subsequent encounter for fracture with nonunion
S62614K	Displaced fracture of proximal phalanx of right ring finger, subsequent encounter for fracture with nonunion
S62615K	Displaced fracture of proximal phalanx of left ring finger, subsequent encounter for fracture with nonunion
S62616K	Displaced fracture of proximal phalanx of right little finger, subsequent encounter for fracture with nonunion
S62617K	Displaced fracture of proximal phalanx of left little finger, subsequent encounter for fracture with nonunion
S62618K	Displaced fracture of proximal phalanx of other finger, subsequent encounter for fracture with nonunion
S62619K	Displaced fracture of proximal phalanx of unspecified finger, subsequent encounter for fracture with nonunion
S62620K	Displaced fracture of middle phalanx of right index finger, subsequent encounter for fracture with nonunion

S62621K	Displaced fracture of middle phalanx of left index finger, subsequent encounter for fracture with nonunion
S62622K	Displaced fracture of middle phalanx of right middle finger, subsequent encounter for fracture with nonunion
S62623K	Displaced fracture of middle phalanx of left middle finger, subsequent encounter for fracture with nonunion
S62624K	Displaced fracture of middle phalanx of right ring finger, subsequent encounter for fracture with nonunion
S62625K	Displaced fracture of middle phalanx of left ring finger, subsequent encounter for fracture with nonunion
S62626K	Displaced fracture of middle phalanx of right little finger, subsequent encounter for fracture with nonunion
S62627K	Displaced fracture of middle phalanx of left little finger, subsequent encounter for fracture with nonunion
S62628K	Displaced fracture of middle phalanx of other finger, subsequent encounter for fracture with nonunion
S62629K	Displaced fracture of middle phalanx of unspecified finger, subsequent encounter for fracture with nonunion
S62630K	Displaced fracture of distal phalanx of right index finger, subsequent encounter for fracture with nonunion
S62631K	Displaced fracture of distal phalanx of left index finger, subsequent encounter for fracture with nonunion
S62632K	Displaced fracture of distal phalanx of right middle finger, subsequent encounter for fracture with nonunion
S62633K	Displaced fracture of distal phalanx of left middle finger, subsequent encounter for fracture with nonunion
S62634K	Displaced fracture of distal phalanx of right ring finger, subsequent encounter for fracture with nonunion
S62635K	Displaced fracture of distal phalanx of left ring finger, subsequent encounter for fracture with nonunion
S62636K	Displaced fracture of distal phalanx of right little finger, subsequent encounter for fracture with nonunion

S62637K	Displaced fracture of distal phalanx of left little finger, subsequent encounter for fracture with nonunion
S62638K	Displaced fracture of distal phalanx of other finger, subsequent encounter for fracture with nonunion
S62639K	Displaced fracture of distal phalanx of unspecified finger, subsequent encounter for fracture with nonunion
S62640K	Nondisplaced fracture of proximal phalanx of right index finger, subsequent encounter for fracture with nonunion
S62641K	Nondisplaced fracture of proximal phalanx of left index finger, subsequent encounter for fracture with nonunion
S62642K	Nondisplaced fracture of proximal phalanx of right middle finger, subsequent encounter for fracture with nonunion
S62643K	Nondisplaced fracture of proximal phalanx of left middle finger, subsequent encounter for fracture with nonunion
S62644K	Nondisplaced fracture of proximal phalanx of right ring finger, subsequent encounter for fracture with nonunion
S62645K	Nondisplaced fracture of proximal phalanx of left ring finger, subsequent encounter for fracture with nonunion
S62646K	Nondisplaced fracture of proximal phalanx of right little finger, subsequent encounter for fracture with nonunion
S62647K	Nondisplaced fracture of proximal phalanx of left little finger, subsequent encounter for fracture with nonunion
S62648K	Nondisplaced fracture of proximal phalanx of other finger, subsequent encounter for fracture with nonunion
S62649K	Nondisplaced fracture of proximal phalanx of unspecified finger, subsequent encounter for fracture with nonunion
S62650K	Nondisplaced fracture of middle phalanx of right index finger, subsequent encounter for fracture with nonunion
S62651K	Nondisplaced fracture of middle phalanx of left index finger, subsequent encounter for fracture with nonunion
S62652K	Nondisplaced fracture of middle phalanx of right middle finger, subsequent encounter for fracture with nonunion

S62653K	Nondisplaced fracture of middle phalanx of left middle finger, subsequent encounter for fracture with nonunion
S62654K	Nondisplaced fracture of middle phalanx of right ring finger, subsequent encounter for fracture with nonunion
S62655K	Nondisplaced fracture of middle phalanx of left ring finger, subsequent encounter for fracture with nonunion
S62656K	Nondisplaced fracture of middle phalanx of right little finger, subsequent encounter for fracture with nonunion
S62657K	Nondisplaced fracture of middle phalanx of left little finger, subsequent encounter for fracture with nonunion
S62658K	Nondisplaced fracture of middle phalanx of other finger, subsequent encounter for fracture with nonunion
S62659K	Nondisplaced fracture of middle phalanx of unspecified finger, subsequent encounter for fracture with nonunion
S62660K	Nondisplaced fracture of distal phalanx of right index finger, subsequent encounter for fracture with nonunion
S62661K	Nondisplaced fracture of distal phalanx of left index finger, subsequent encounter for fracture with nonunion
S62662K	Nondisplaced fracture of distal phalanx of right middle finger, subsequent encounter for fracture with nonunion
S62663K	Nondisplaced fracture of distal phalanx of left middle finger, subsequent encounter for fracture with nonunion
S62664K	Nondisplaced fracture of distal phalanx of right ring finger, subsequent encounter for fracture with nonunion
S62665K	Nondisplaced fracture of distal phalanx of left ring finger, subsequent encounter for fracture with nonunion
S62666K	Nondisplaced fracture of distal phalanx of right little finger, subsequent encounter for fracture with nonunion
S62667K	Nondisplaced fracture of distal phalanx of left little finger, subsequent encounter for fracture with nonunion
S62668K	Nondisplaced fracture of distal phalanx of other finger, subsequent encounter for fracture with nonunion

S62669K	Nondisplaced fracture of distal phalanx of unspecified finger, subsequent encounter for fracture with nonunion
S6290XK	Unspecified fracture of unspecified wrist and hand, subsequent encounter for fracture with nonunion
S6291XK	Unspecified fracture of right wrist and hand, subsequent encounter for fracture with nonunion
S6292XK	Unspecified fracture of left wrist and hand, subsequent encounter for fracture with nonunion
